# Temperature mediates variation in avian sensitivity to forest cover

**DOI:** 10.1038/s41559-026-03160-9

**Published:** 2026-08-28

**Authors:** Natasha R. Granville, Jessica J. Williams, Vivienne P. Groner, Jos Barlow, Matthew G. Betts, Gergana N. Daskalova, Robert M. Ewers, Luiz dos Anjos, Victor Arroyo-Rodriguez, Luc Barbaro, Alexis Cerezo, Pedro F. Develey, Jack H. Hatfield, Hervé Jactel, Jordan Karubian, Urs G. Kormann, Jesse R. Lasky, Charles J. Marsh, Luiz A. M. Mestre, José Carlos Morante-Filho, Pieter Olivier, Benjamin T. Phalan, Anna M. Pidgeon, Vânia Proença, Julien Terraube, Alexandre Uezu, Eric M. Wood, Alex L. Pigot, Cristina Banks-Leite

**Affiliations:** 1 Georgina Mace Centre for the Living Planet, Department of Life Sciences, Imperial College London, Ascot, UK; 2 Lancaster Environment Centre, Lancaster University, Lancaster, UK; 3 Department of Forest Ecosystems and Society, Peavy Forest Sciences Center, Oregon State University, Corvallis, OR USA; 4 Department of Conservation Biology, University of Goettingen, Göttingen, Germany; 5 Laboratory of Biodiversity, State University of Londrina, Londrina, Brazil; 6 Instituto de Investigaciones en Ecosistemas y Sustentabilidad, Universidad Nacional Autónoma de México, Morelia, Mexico; 7 Escuela Nacional de Estudios Superiores, Universidad Nacional Autónoma de México, Yucatán, Mexico; 8 Dynafor, University of Toulouse, INRAE, Castanet-Tolosan, France; 9 Foundation for Ecodevelopment and Conservation (FUNDAECO), Ciudad de Guatemala, Guatemala; 10 SAVE Brasil, São Paulo, Brazil; 11 Leverhulme Centre for Anthropocene Biodiversity and Department of Biology, University of York, York, UK; 12 INRAE, University of Bordeaux, Cestas, France; 13 Department of Ecology and Evolutionary Biology, Tulane University, New Orleans, LA USA; 14 Swiss Ornithological Institute, Sempach, Switzerland; 15 Department of Biology, Pennsylvania State University, University Park, PA USA; 16 Department of Biological Sciences, National University of Singapore, Singapore, Singapore; 17 Center for Nature-based Climate Solutions, National University of Singapore, Singapore, Singapore; 18 Ciências Ambientais, Universidade Federal do Paraná, Setor Litoral, Matinhos, Brazil; 19 Departamento de Ciências Biológicas, Universidade Estadual de Santa Cruz, Bahia, Brazil; 20 M.A.P Scientific Services, Pretoria, South Africa; 21 Department of Zoology and Entomology, University of Pretoria, Pretoria, South Africa; 22 Department of Forest and Wildlife Ecology, University of Wisconsin-Madison, Madison, WI USA; 23 MARETEC/LARSyS, Instituto Superior Técnico, Universidade de Lisboa, Lisbon, Portugal; 24 OFB - Office français de la biodiversité - Direction de la recherche et de l’appui scientifique - Service conservation et gestion durable des espèces exploitées, Pérols, France; 25 Faculty for Environmental Conservation and Sustainability (ESCAS), IPÊ – Institute for Ecological Research, Nazaré Paulista, Brazil; 26 Department of Biological Sciences, California State University, Los Angeles, CA USA; 27 Centre for Biodiversity and Environment Research, Department of Genetics, Evolution and Environment, University College London, London, UK; 28 Departamento de Ecologia, Instituto de Biociências, Universidade de São Paulo, São Paulo, Brazil

**Keywords:** Macroecology, Conservation biology, Biodiversity, Forest ecology, Biogeography

## Abstract

Emerging evidence shows that responses to deforestation can differ both across species and among populations of the same species. The reasons underlying these complex patterns are unclear, and this lack of clarity is a barrier to accurate monitoring and prediction of biodiversity change. Using data from 2,262 bird species across 7,326 sites from all forested continents, we show that between-species and within-species variations in responses to forest cover are mediated by temperature. Populations in warmer macroclimates tend to decline in incidence in deforested landscapes because hotter microclimates push them closer to their species’ realized upper thermal tolerance limits. By contrast, populations in cooler macroclimates tend to be less affected by or even benefit from deforestation, as microclimatic temperatures are pushed closer to more optimal temperatures. Our findings offer an empirically grounded framework for biodiversity models that move beyond fixed species responses and incorporate interactions between temperature and land-use change.

## Abstract (web summary)

Land-use and climate change may have synergistic impacts on biodiversity. This global analysis shows that the sensitivity of bird species and populations to deforestation is modulated by their thermal ecology and forest cover effects on microclimate.

## Main

Global biodiversity change, driven primarily by land-use change^1,2^, is among the most complex challenges facing humanity today. Nearly one-third of forests have already been lost^3^, and three-quarters of remaining forests lie within 1 km of non-forested areas^4^. At the 30th United Nations Climate Change Conference (COP30), nations committed to protecting natural habitats and minimizing species extinction risks^5^. Achieving these goals requires understanding of how land-use change influences loss or persistence of species across their geographic ranges. Past research has primarily focused on interspecific variability in the effects of land-use change, some of which is related to species’ traits^6–8^, climatic niches^9^ and disturbance histories^10^. By contrast, intraspecific variability, which is a cornerstone of species evolution and adaptation to new environments^11–15^, is often overlooked in global biodiversity conservation strategies^16^. Although intraspecific variability in responses to climate change is relatively well documented^17^, studies addressing habitat loss typically assume uniform responses across all populations within a species^18,19^. Emerging evidence of intraspecific variability in the impact of habitat loss^20–25^ raises the questions of how ubiquitous this phenomenon is, what causes it and what its implications are for biodiversity conservation.

The few studies that have explored the causes of intraspecific variability in the effects of land use suggest that macroecological factors play a key role^18^, but the underlying reasons have remained elusive. For instance, some studies have shown that bird populations are more sensitive to changes in forest cover when located near their species’ geographic range limits^20,21,24^, a trend that could be influenced by the climatic conditions associated with species’ range limits^25,26^. Indeed, vertebrate populations situated closer to their species’ realized climatic tolerance limits are more sensitive to land use^27^, and mammal populations in North America have been shown to be more dependent on forests when located in hotter macroclimates^22^. Heightened sensitivity to forest cover near species’ warm range limits could be attributed to exposure of species to hotter, drier and more variable microclimates as a result of land-use change^28,29^. Macroecological variability in ectotherm responses to climate change has been shown to be mediated by species-specific proximity to thermal limits^30^, but these concepts have seldom been used to understand responses to land-use change. Theoretical studies have suggested that integrating thermal biology concepts could advance our understanding of how land-use change affects biodiversity^31,32^, but there have been few empirical studies^33^, especially on endotherms.

**Fig. 1:**
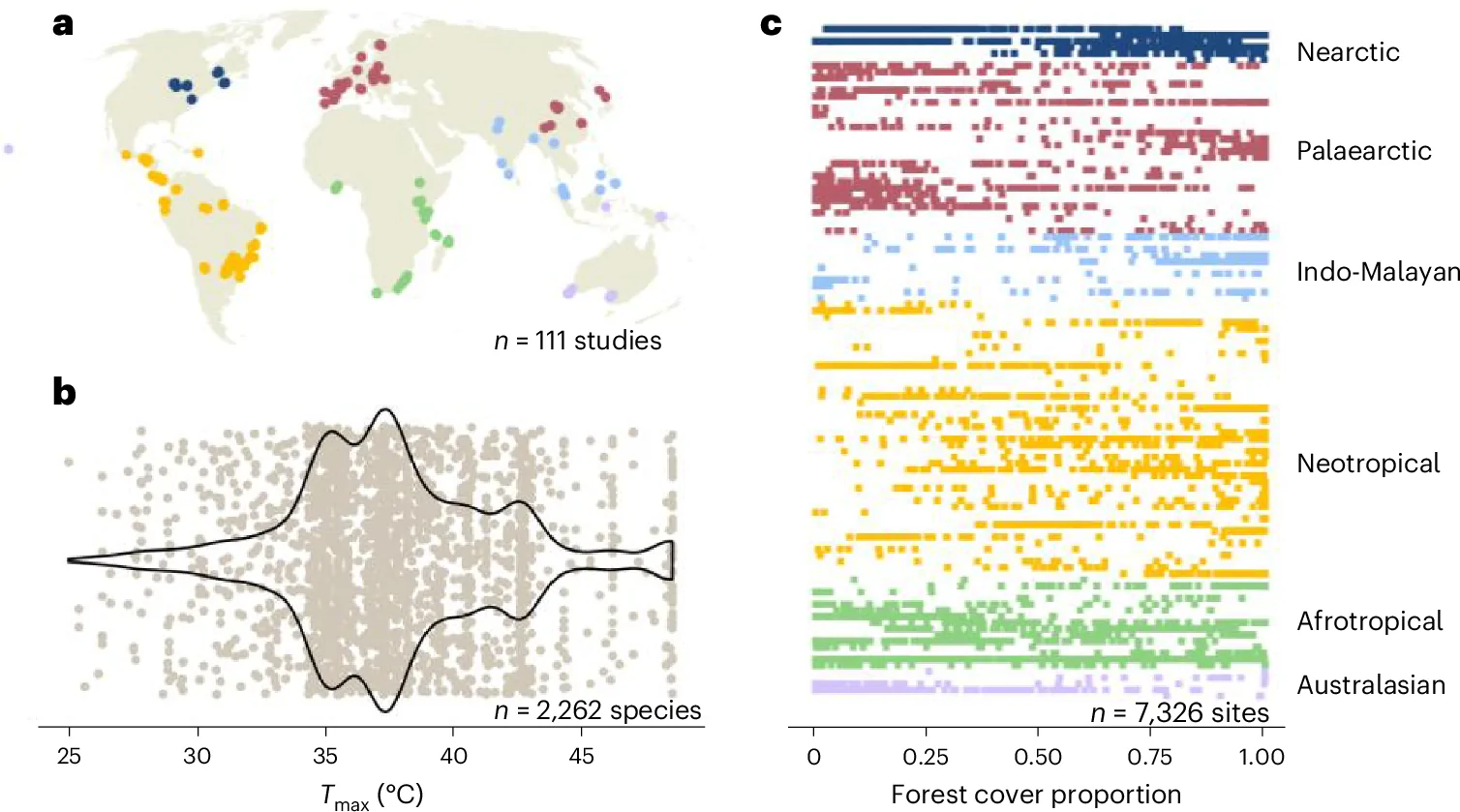
Global dataset spanning the full range of biogeographic realms, warm limits and forest cover proportions. **a**, Spatial distribution of bird studies. **b**, Violin plot showing distribution of species’ realized warm tolerance limit (*T*_max_) values. A species’ realized warm tolerance limit was defined as the hottest macroclimatic temperature within the species’ geographic range. Each point represents a species, with points jittered randomly along the *y* axis to reduce overlap and improve visibility. **c**, Forest cover proportions within and across studies. Each row represents one study, with individual points indicating the forest cover proportion at each site within that study. Basemap in **a** from Natural Earth (https://www.naturalearthdata.com/), obtained via R package ggplot2.

Here we investigated how temperature is associated with inter- and intraspecific variability in how forest birds respond to forest cover. We first analysed how macroclimate temperature affected responses to forest cover within and across species. Then, we used mechanistic microclimate modelling to propose an explanation for this macroecological variation. Assessment of inter- and intraspecific variability at macroecological scales requires both broad geographic data and detailed regional sampling to capture populations at different positions within their species’ range, which is why it has rarely been attempted before. Therefore, we compiled presence–absence (incidence) data across landscapes of varying forest cover from several databases^34–36^ (Methods). The data within these databases come from studies designed to understand the responses of ecological communities to land-use change. We restricted our analysis to studies conducted within forest ecoregions^37^ and species for which forest was listed as one of their habitat affiliations according to the International Union for Conservation of Nature (IUCN) habitat classifications^38^. In total, our global dataset included incidence data from 2,262 bird species at 7,326 sample sites, collected from 111 studies conducted in forests between 1996 and 2019 (Fig. 1 and Supplementary Table 1). More than half (60%) of the species were surveyed in more than one study, which allowed us to assess intraspecific variability.

Our global analysis employed a space-for-time substitution, comparing bird incidence probabilities across sites spanning gradients in forest cover, located within forest ecoregions, to infer how populations might respond to deforestation. To examine the degree to which species incidence was affected by landscape-scale forest cover, we used a 30-m-resolution global dataset of tree cover in the year 2000^39^ to calculate the forest cover proportion (hereafter referred to as ‘forest cover’) within a 600-m-radius circular buffer around each sample site (Methods). This buffer size reflected the sampling design of many of the original studies in the dataset and has been shown to be optimal for assessment of bird responses to forest cover^40–44^. As the effects of environmental changes persist over time, and species often show lagged responses to these changes^45–48^, we expected that species surveyed during or after 2000 would have been influenced by the conditions in 2000.

To investigate how temperature affected bird sensitivity to forest cover, we estimated species’ realized warm tolerance limits from their geographic ranges^9,27,49^. As these ranges reflect the conditions under which species currently persist, they provide a powerful opportunity to estimate realized thermal limits at global scales at which obtaining experimental data on physiological tolerance limits would not be feasible^27,49^. Using ERA5 hourly macroclimate temperature data from 2000 at 0.25° spatial resolution^50^, we calculated the mean daily maximum temperature of the warmest month and identified the hottest temperature within each species’ geographic range. This represented the upper envelope of regional macroclimatic conditions associated with the mapped range, which provided an estimate of each species’ realized warm tolerance limit^27^; this is hereafter referred to as the species’ warm limit and abbreviated as *T*_max_.

We also extracted the mean daily maximum temperature of the warmest month at each sample site. For each combination of site and species, we calculated the difference between the temperature at the site and the species’ warm limit, hereafter referred to as the *T*_max_ distance. A population with a *T*_max_ distance of zero would be located in an environment with a macroclimatic temperature equivalent to the species’ warm limit. Higher *T*_max_ distance values indicate populations in relatively cooler temperatures, located further from the species’ warm limit. For each population, we investigated how *T*_max_ distance moderated the effect of forest cover on the species’ incidence probability.

## Results and discussion

### Inter- and intraspecific variation in sensitivity to forest cover

**Fig. 2:**
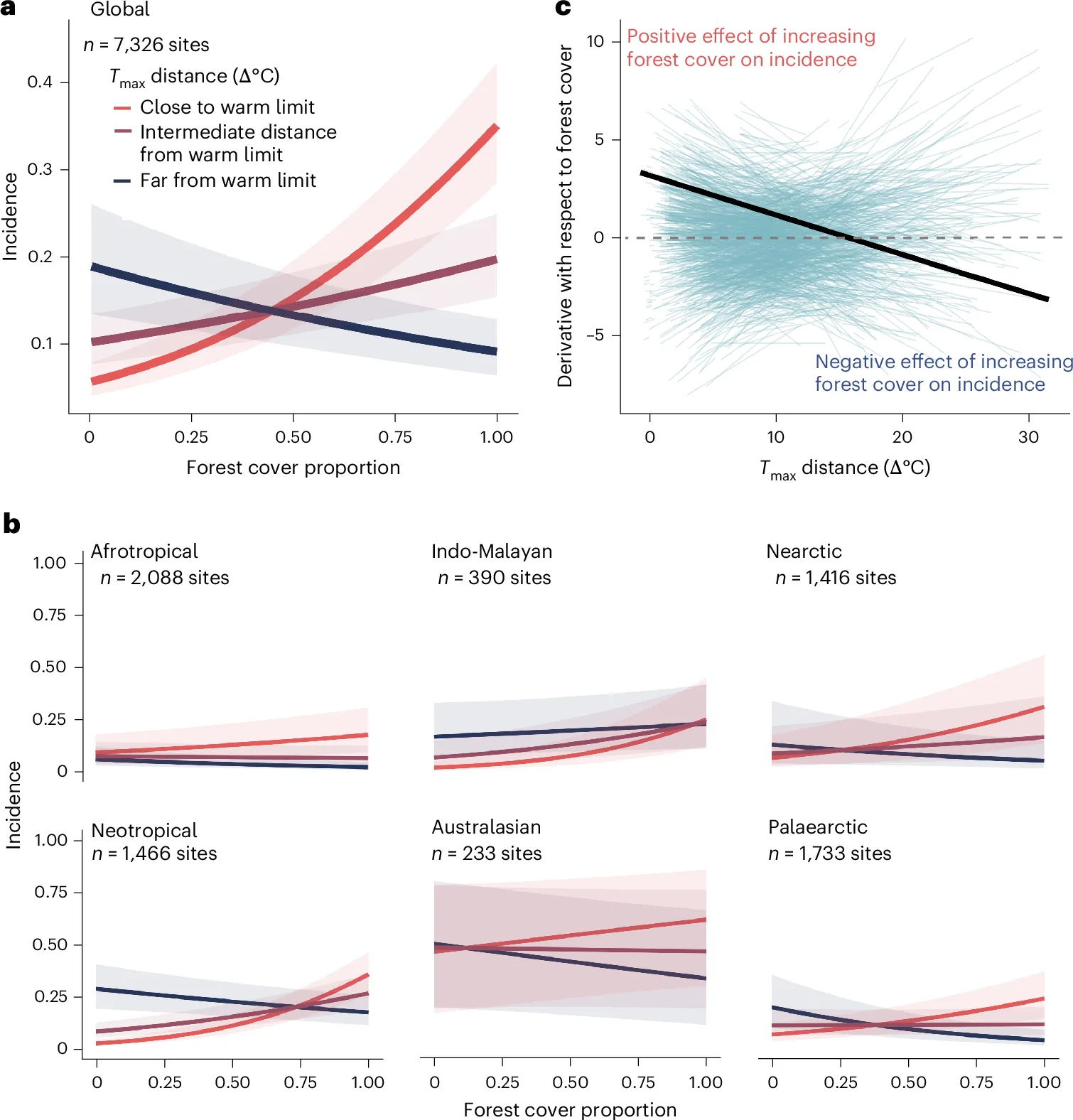
The location of a population relative to its species’ realized warm tolerance limit (*T*_max_ distance) can mediate variation in how forest cover affects bird incidence. **a**,**b**, Influence of forest cover proportion within a 600-m radius on bird incidence across populations at different distances from their species’ realized warm tolerance limits (*T*_max_ distance): the global pattern (**a**) and results by biogeographic realm (**b**) are shown. Lines represent mean responses for populations near to (pink line, 10th percentile), an intermediate distance from (purple, 50th percentile) and far from (blue, 90th percentile) these limits. Shading indicates 95% confidence intervals. For the results in **a**, the 10th percentile of *T*_max_ distance was 5 °C from the warm limit, the 50th percentile was 12 °C and the 90th percentile was 20 °C from the warm limit. The *T*_max_ distances represented by each of the percentiles within each biogeographic realm in **b** can be found in Extended Data Fig. 1. **c**, Responses for each species, with each thin line representing one species. The *y* axis indicates the direction and strength of the response to forest cover, and the grey dashed line marks the point at which the response to increasing forest cover changed from negative to positive. The thick black line shows the overall mean relationship across all species: populations closer to their species’ realized warm tolerance limits generally showed more positive responses to increases in forest cover when all species were analysed collectively.

**Extended Data Fig. 1:**
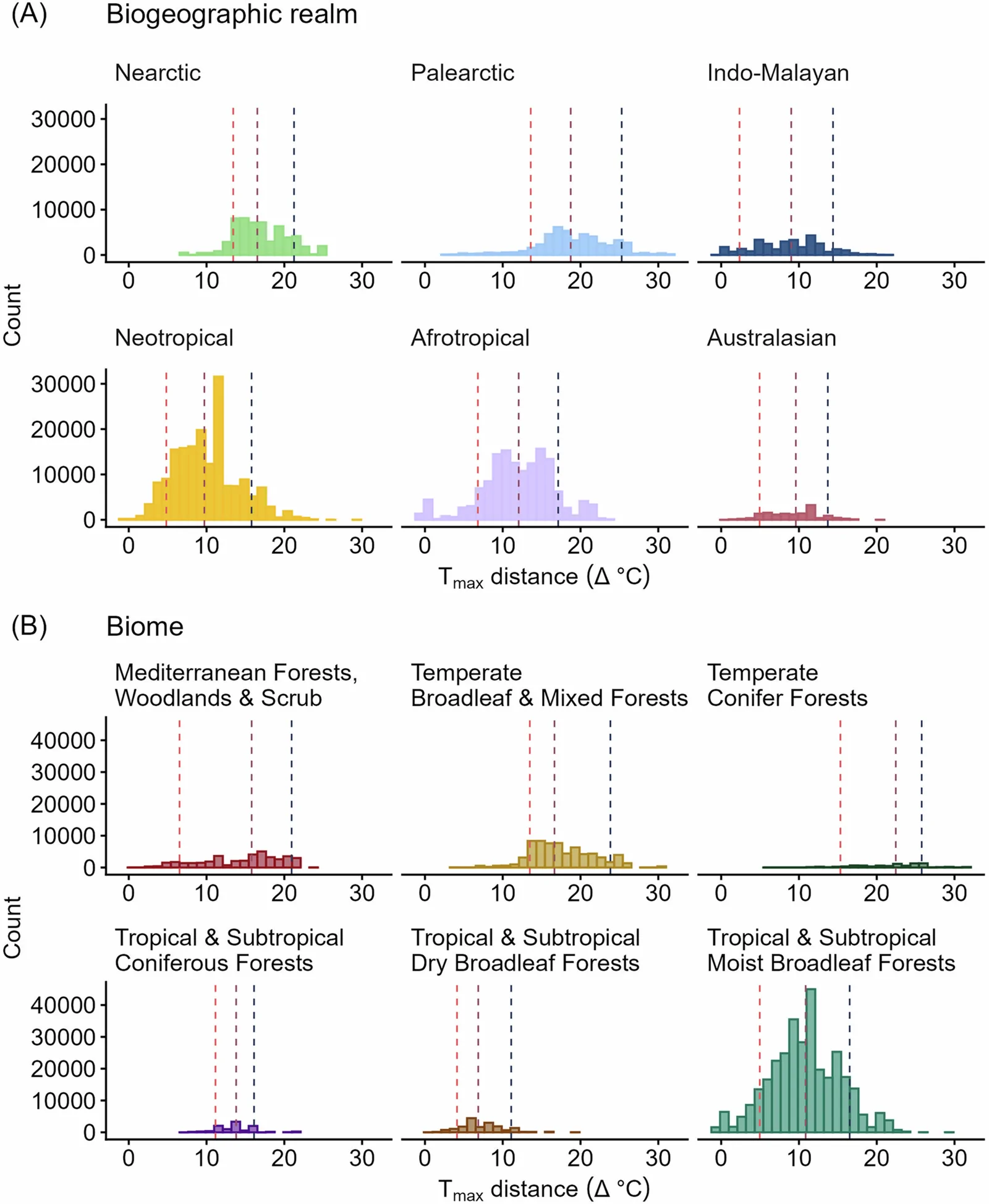
Distribution of *T*_max_ distance within each realm (A) and biome (B). The y-axes show counts of species-site combinations. The vertical dashed lines show the 10^th^ (red), 50^th^ (purple) and 90^th^ (blue) percentiles of *T*_max_ distance within each realm or biome. These percentiles correspond to those plotted in Fig. 2b (realms) and Extended Data Fig. 2 (biomes).

**Extended Data Fig. 2:**
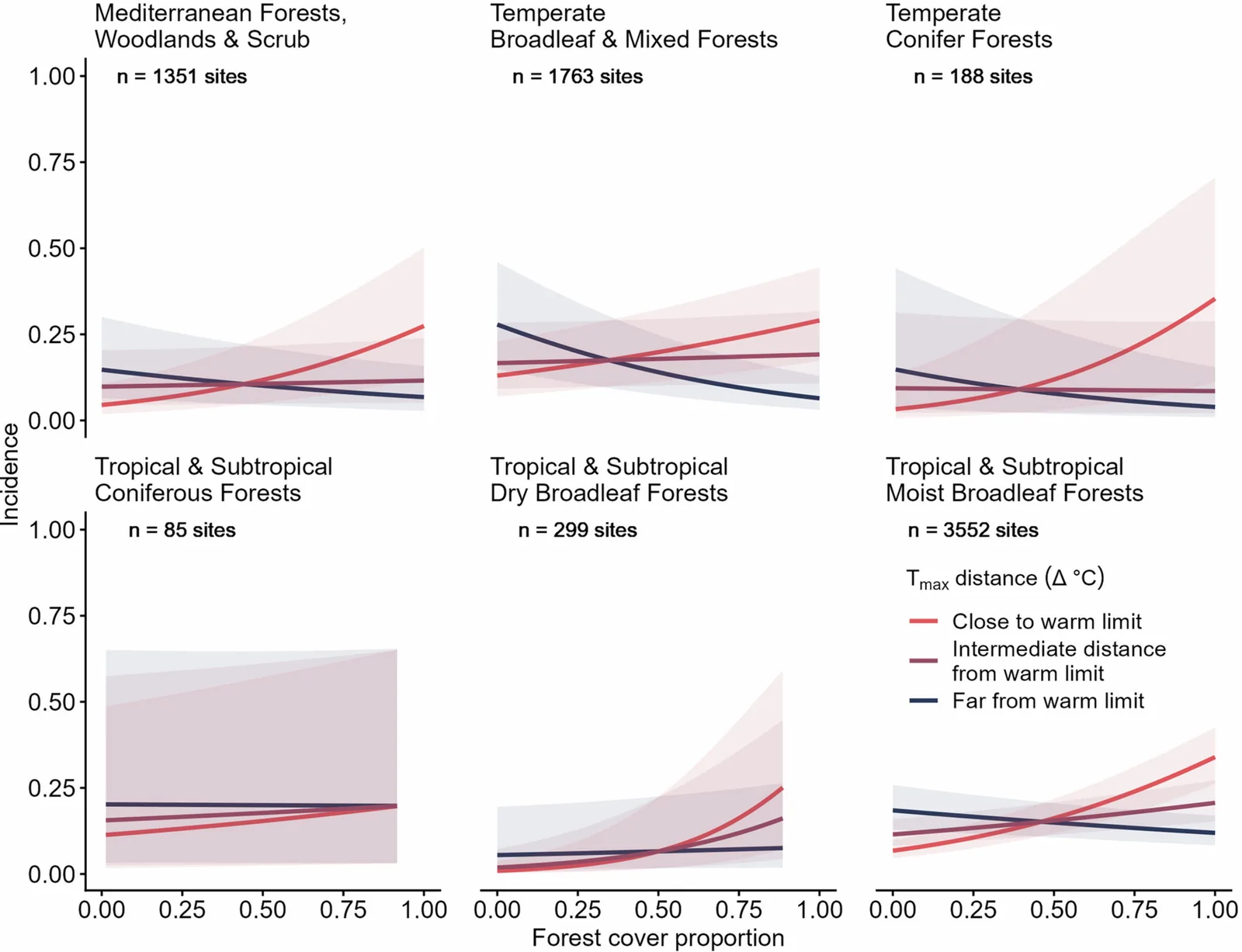
Within biomes, the location of a population relative to its species’ realised warm tolerance limit (*T*_max_ distance) mediates variation in how forest cover (x-axis) affects the probability of bird incidence (y-axis). The results are split into panels according to the biome in which the study was located and, within each panel, the lines represent the mean responses for populations near (pink line, 10^th^ percentile), intermediate (purple, 50^th^ percentile) and far (blue, 90^th^ percentile) from their species’ realised warm tolerance limits. The *T*_max_ distance values represented by each of these percentiles within each biome can be found in Extended Data Fig. 1. The shading around the lines shows the 95% confidence intervals.

We investigated how *T*_max_ distance and forest cover affected the probability of species’ incidence using a binomial generalized linear mixed model (GLMM). This model included forest cover proportion, *T*_max_ distance and the interaction between these two variables as fixed-effect predictors of incidence. Both variables had positive effects on incidence overall (effect sizes on log-odds scale: 3.2 for forest cover, s.e. = 0.2, *P* < 0.001; 0.09 for *T*_max_ distance, s.e. = 0.01, *P* < 0.001). However, there was a negative interaction between these two predictors (−0.2, s.e. = 0.02, *P* < 0.001), indicating that responses to forest cover varied with *T*_max_ distance. Increasing forest cover had a stronger positive effect on incidence for populations located in macroclimates closer to their species’ warm limits (lower *T*_max_ distance). This positive effect of increasing forest cover on incidence was weaker and even switched to a negative effect for populations located in macroclimates further from their species’ warm limits (higher *T*_max_ distance). Equally, bird incidence was highest at macroclimate temperatures closer to the species’ warm limit at high levels of forest cover, whereas at low levels of forest cover, incidence was higher at macroclimate temperatures further from the species’ warm limit (Fig. 2a and Supplementary Table 2). We found the same pattern across all biomes and realms, although the interaction between *T*_max_ distance and forest cover was strongest in the Nearctic and Neotropical realms and weakest in the Australasian realm (Fig. 2b, Extended Data Fig. 2 and Supplementary Tables 3 and 4). However, sample sizes and spans of *T*_max_ distance differed among realms and biomes (Extended Data Fig. 1).

**Extended Data Fig. 3:**
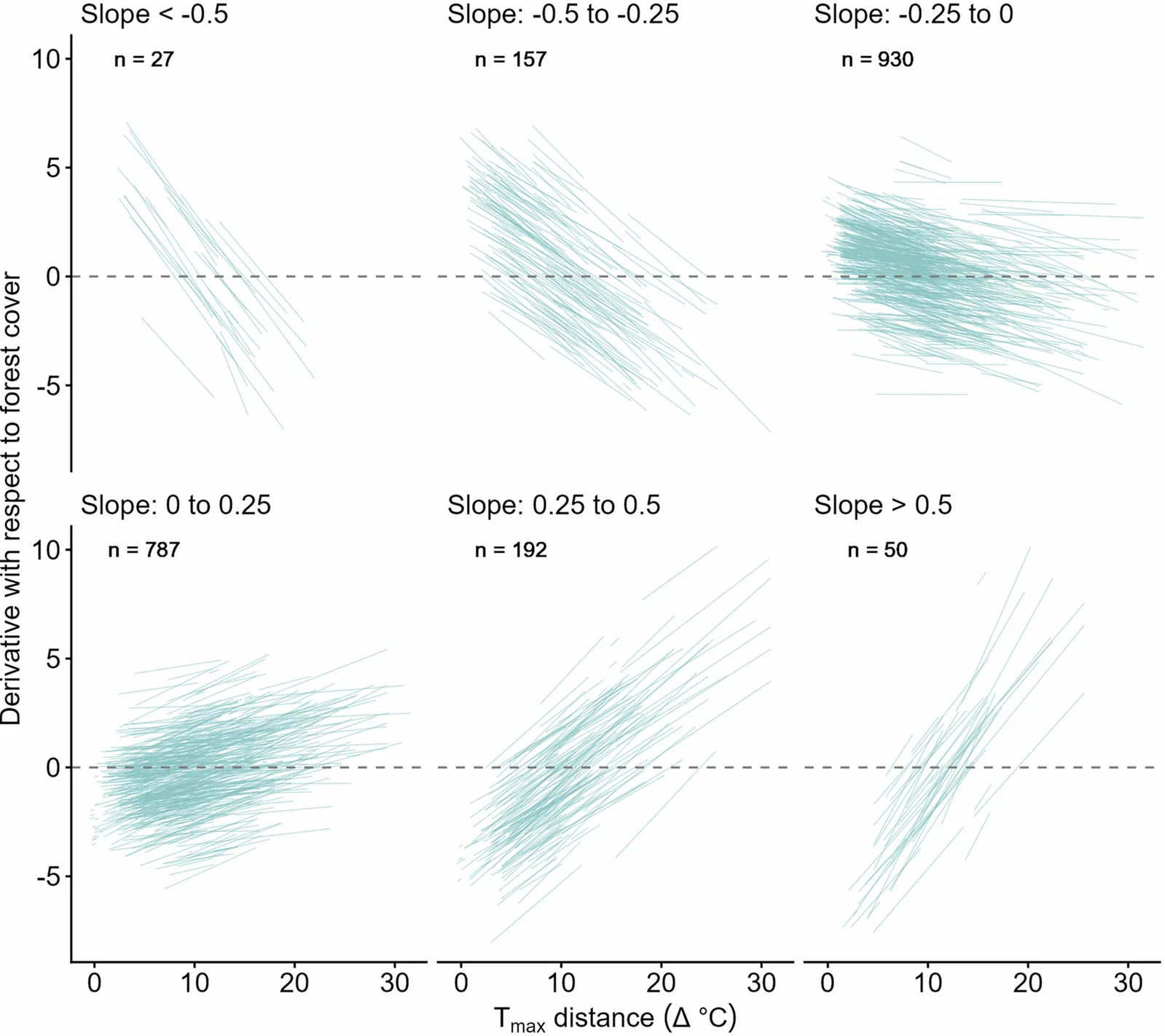
Within species, population responses to forest cover vary with *T*_max_ distance. Each thin blue line shows the response of one species. The y-axis shows the derivative with respect forest cover, which provides a metric of how incidence varies with forest cover – a positive value is an increase in incidence with increasing forest cover and a negative value is a decrease in incidence with increasing forest cover. The grey dotted line shows the point where the response to forest cover changes from negative to positive. The x-axis shows *T*_max_ distance, where lower *T*_max_ distance corresponds to macroclimatic temperatures closer to the species’ warm limit. Species are split into panels according to the slope of the relationship between *T*_max_ distance and the derivative with respect to forest cover.

*T*_max_ distance is an important modulator of the variation in how forest cover affects incidence both among species (Fig. 2) and among populations of the same species (Extended Data Fig. 3). To study this relationship within each species, we calculated the derivative of the species-level random effects^20^ to provide a metric of response to forest cover at any given *T*_max_ distance for each species. A positive value of this metric indicated an increase in population incidence with increasing forest cover, and a negative value indicated a decrease in incidence with increasing forest cover. Of the 2,262 species considered in our study, 2,143 could be used to assess how *T*_max_ distance modulated within-species responses to forest cover, as these species showed some variation in *T*_max_ distance either within or across studies. Of these 2,143 species, 1,114 (52%) showed increasingly positive effects of forest cover on incidence when located closer to *T*_max_ (Extended Data Fig. 3), indicating substantial species-level heterogeneity. We also calculated the partial effect of forest cover for the fixed-effects component of the model. This showed an overall mean trend in which increasing forest cover had a positive effect on incidence at temperatures close to *T*_max_ and a shift in the average response to a negative effect of increasing forest cover on incidence at temperatures >15.8 °C cooler than *T*_max_, when all species were analysed together (Fig. 2c).

**Extended Data Fig. 4:**
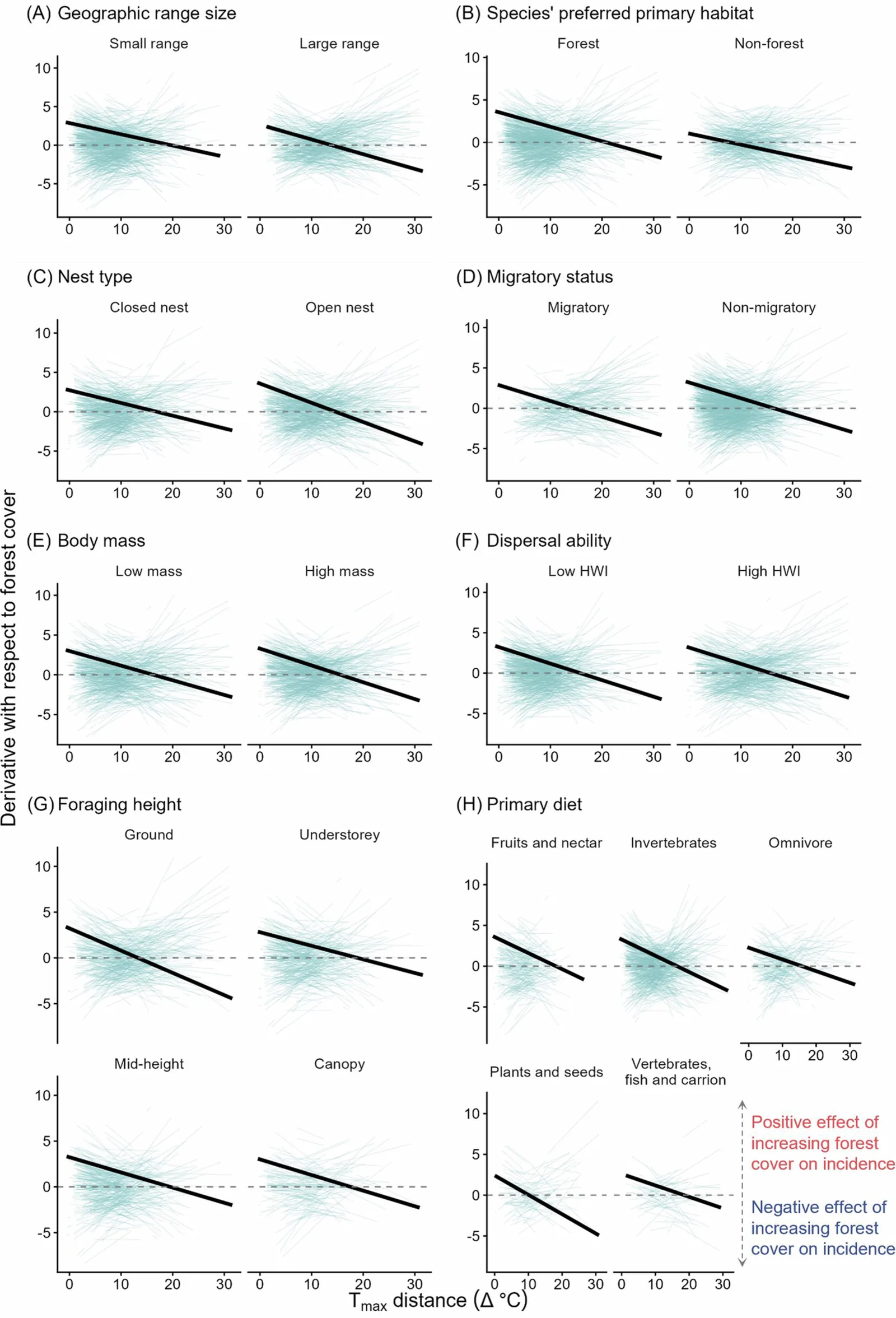
Across species with different traits, populations located closer to their species’ warm limits (lower *T*_max_ distances) generally show more positive effects of increasing forest cover on incidence. In each panel, the y-axis shows how incidence varies with forest cover, where a positive value is an increase in incidence with increasing forest cover and a negative value is a decrease in incidence with increasing forest cover. The grey dotted line shows the point where the response to forest cover changes from negative to positive. The x-axis shows *T*_max_ distance, where lower *T*_max_ distances correspond to macroclimatic temperatures closer to the species’ warm limit. The thin blue lines show the individual species responses, and the thick black lines show the average response across all species. Species are split into panels based on their traits. In (**a**), species are split into two panels according to whether their geographic range size was lower or higher than the median geographic range size of all species in the dataset. In (**b**) species are split into two groups: those whose primary habitat preference, as listed in the AVONET database^74^, is forest, and those whose primary habitat preference is a habitat other than forest. In (**c**), species are split into two groups: those that build open (for example cup-shaped or platform-shaped) nests and those that build closed (for example dome-shaped or cavity) nests. In (**d**), species are split into those that migrate (including both fully and partially migratory species) and those that do not migrate. In (**e**), species are split into those with a body mass lower than the median body mass of species in the dataset and those with a body mass higher than the median. In (**f**), species are split into those with a hand-wing index (HWI, proxy for dispersal ability) lower than the median HWI of species in the dataset and those with a HWI higher than the median. In (**g**), species are split into four groups based on their primary foraging height. In (**h**), species are split into five groups based on their primary diet.

We investigated whether the effect of *T*_max_ distance on response to forest cover was a general trend or whether it was context dependent, varying among species with different traits and ecological niches. We tested a suite of traits: geographic range size, primary habitat preference, nest type, migratory status, body mass, hand-wing index, primary diet and primary foraging height. We chose these traits because they have been shown to affect species’ sensitivity to forest cover or temperature^7,8,51–54^. Species’ geographic range size was the strongest predictor of variation in how *T*_max_ distance affected response to forest cover (Extended Data Fig. 4 and Supplementary Table 7). Species with larger ranges were more likely to show more positive effects of forest cover on incidence close to *T*_max_ and more negative effects of forest cover on incidence further from *T*_max_. By contrast, species with small ranges were likely to always be located close to their *T*_max_ and so were likely to be highly sensitive to forest cover throughout their range.

Primary habitat also mediated variation in how *T*_max_ distance affected response to forest cover. Species with forest as their primary habitat preference were more likely to show positive responses to forest cover close to *T*_max_ and more negative responses to forest cover further from *T*_max_ (Extended Data Fig. 4 and Supplementary Table 8). Open-nest species were slightly more likely to show this pattern, although the difference between open- and closed-nest species was relatively small (Extended Data Fig. 4 and Supplementary Table 9). The relationship between *T*_max_ distance and response to forest cover was consistent regardless of all other traits that we tested (Extended Data Fig. 4 and Supplementary Tables 10–14), indicating that the dependence of bird responses to forest cover on macroclimate is a general pattern that does not depend strongly on species’ morphological or dietary traits.

Our results provide robust evidence that some of the variability in response to forest cover within and among species can be explained by the proximity of a population to its species’ warm limit (Fig. 2). Understanding this variation could provide insight into how populations respond to changes in forest cover and could therefore help to identify places where populations are likely to be most vulnerable to deforestation and where protection and restoration of forests are likely to be more beneficial. Our results challenge the prevailing notion that sensitivity to forest cover is a trait fixed at the species level, particularly for species with large ranges that showed strong variation in response to forest cover. This has important implications for current approaches to conservation, which tend to rely on species-level classifications of threat responses. Although these results identify general trends in sensitivity within and across species, they do not directly reveal the processes underlying these patterns.

### Thermal exposure can explain variation in sensitivity to forest cover

**Extended Data Fig. 5:**
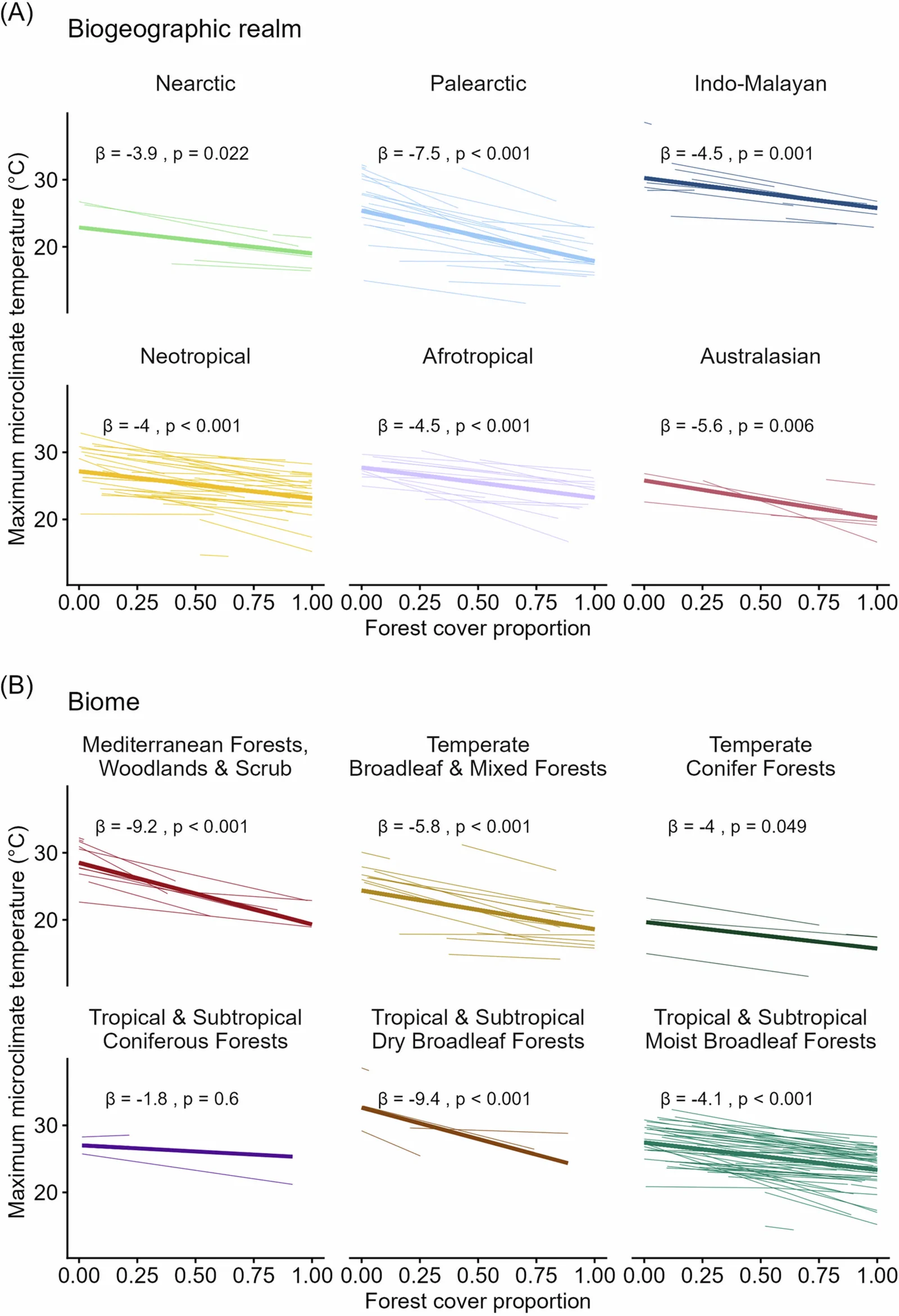
Forests buffer warm microclimate temperatures. (**a**) Studies grouped by realm and (**b**) by biome. The y-axis shows mean daily maximum temperature of the warmest month from microclimate modelling, Thin lines represent individual studies, extracted from the random effects of a linear mixed effects model. Thick lines, slope coefficients and p-values show overall mean relationships from the fixed effects of a linear mixed effects model. We fitted one linear mixed effects model to test for differences across realms (**a**) and another for biomes (**b**).

To better understand the potential processes driving variability in population responses, we examined how forest cover affected the local microclimatic temperatures that species experienced and how these microclimatic temperatures in turn affected species incidence. Given that forest loss raises local microclimatic maximum temperatures, we hypothesized that biotic responses to microclimatic temperatures could be key to understanding inter- and intraspecific variability in responses to forest cover. Maximum temperatures beneath forest canopies tend to be lower than surrounding non-forested areas because forest canopies intercept direct sunlight and promote increased evaporative cooling^29,55^. Thus, *T*_max_ distance based on the macroclimate of the sample site may not represent the temperatures that birds experience inside forests^56^. For this reason, we estimated the local microclimatic temperature of the hottest month, using a mechanistic microclimate model^57^ that integrated high-resolution vegetation data including canopy height and leaf-area index (LAI) (Methods). This model did not include the forest cover data that was used as a predictor in our statistical models to prevent circularity in our approach. Across all biogeographic realms and biomes, microclimatic temperatures were cooler at higher levels of forest cover (Extended Data Fig. 5 and Supplementary Tables 15 and 16). For each combination of site and species, we calculated the difference between the microclimatic temperature at the sample site and the species’ macroclimatic warm limit to produce the micro*T*_max_ distance of each species at each site.

**Fig. 3:**
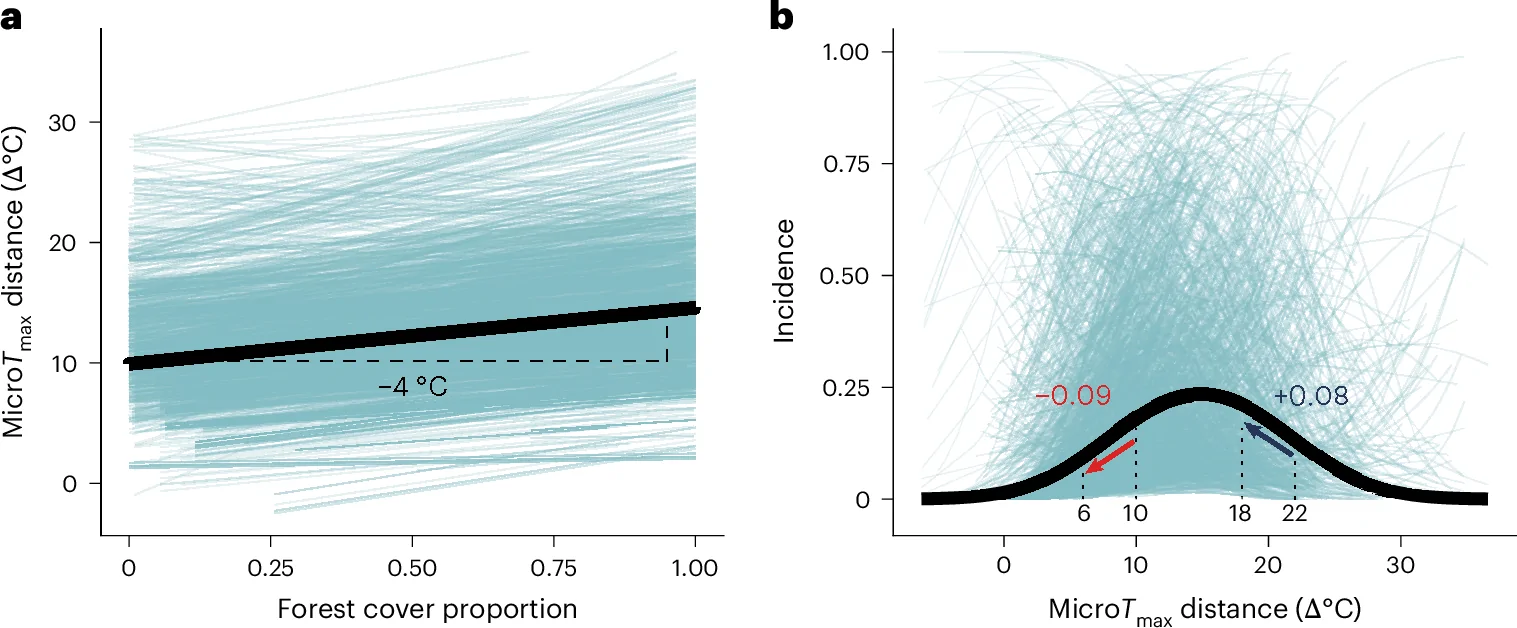
On average, sites with low forest cover have microclimatic temperatures 4 °C warmer than sites with high forest cover, which can have contrasting effects on species’ incidence probability. **a**, Lower forest cover was associated with a reduction in micro*T*_max_ distance. Lower micro*T*_max_ distances indicate microclimatic temperatures closer to a species’ warm limit. The thick black line shows the average relationship across all species, and thin blue lines show the relationships for each species. For a change in forest cover from 95% to 5%, there was a reduction of 4 °C in the average micro*T*_max_ distance. **b**, Effect of micro*T*_max_ distance on incidence. The thick black line shows the overall relationship, and thin blue lines show the relationships for each species. An example of how a decrease of 4 °C in micro*T*_max_ distance can have contrasting effects on species’ incidence depending on the starting micro*T*_max_ distance is shown. The microclimatic shift associated with lower forest cover tended to negatively affect populations in relatively warm regions of their species’ ranges but could benefit populations in regions that are cool relative to species’ optimal temperatures.

By modelling the microclimate, we were able to quantify the extent to which declining forest cover pushed populations closer to their species’ warm limit. For every 10% increase in forest cover, we found an average decrease of 0.47 °C (95% confidence interval (CI): 0.45, 0.48) in micro*T*_max_ distance, such that sites with 95% forest cover were on average 4.2 °C (95% CI: 4.0, 4.3) further from the species’ warm limits than sites with 5% forest cover (Fig. 3a and Supplementary Table 5).

Unimodal responses to temperature are well characterized in ectotherms; performance reaches its peak at the species’ optimal body temperature and declines at thermal extremes^58^. This typically translates to corresponding changes in population size^59,60^. Comparable unimodal effects of external environmental temperature on population size have been hypothesized for endotherms^31,61,62^, because prolonged exposure to environmental temperatures near species’ thermal limits can compromise survival, foraging and reproduction. Empirical evidence has shown that extreme heat is associated with reductions in bird abundance^63^. These changes could occur through direct physiological effects such as heat-induced mortality^64^ and reduced nesting success^52^, as well as indirect effects of temperature, for instance, effects on resource availability or biotic interactions^65^.

We investigated how probability of incidence varied with micro*T*_max_ distance and found a unimodal relationship between micro*T*_max_ distance and incidence when averaging across all species. On average, species reached their peak probability of incidence at 14.9 °C (95% CI: 11.5, 19.3) from their warm limit (Fig. 3b). The micro*T*_max_ distance values that support peak probabilities of incidence are hereafter referred to as ‘optimal’ temperatures. In contrast to the direct physiological optima that are typical of ectotherms, the optimal temperatures that we describe here could have a variety of direct and indirect causes that enable environmental conditions conducive to peaks in incidence probability for birds^66,67^.

Understanding bird responses to microclimatic temperature could enable us to explain some of the variation among populations in responses to forest cover. Populations located in regions where they experience warmer-than-optimal environmental temperatures are pushed too close to their species’ warm limit at low levels of forest cover and therefore would tend to decrease in incidence with reductions in forest cover. For instance, a reduction in micro*T*_max_ distance from 10 °C to 6 °C from the warm limit was associated with an average decrease in probability of incidence of −8.6% (95% CI: −14, −4). By contrast, populations located in regions with cooler-than-optimal temperatures are brought closer to their optimal temperatures at low levels of forest cover, and therefore incidence would be likely to increase with reductions in forest cover. For instance, a reduction in micro*T*_max_ distance from 22 °C to 18 °C from the warm limit was associated with an average increase in probability of incidence of +7.8% (95% CI: 1.5, 14) (Fig. 3b and Supplementary Table 6).

By analysing bird responses in landscapes of varying forest cover proportions in forest ecoregions that are assumed to have been almost entirely forested before anthropogenic disturbance, our study provides insight into the effects of deforestation on bird assemblages and how these effects vary with temperature. The use of a space-for-time substitution enabled us to investigate global variation in responses across a wide, comprehensive gradient of forest cover. However, we emphasize the need for future studies using temporal data to directly analyse the temperature mediation of how deforestation affects bird assemblages.

Our results show that maximum temperature is an important mediator of variation in responses to forest cover, which can be explained by local microclimatic changes. It is important to consider that this thermal effect could be confounded because temperature gradients can covary with latitude, and other environmental (for example, seasonality), ecological (for example, dispersal ability) and historical (for example, disturbance) factors that have previously been linked to bird responses to forest cover ^8,10,68^. However, the effect of *T*_max_ distance on response to forest cover remained the same even when we included latitude as an additional predictor in our model. Whereas latitude was significant in isolation, it was no longer a significant predictor of responses to forest cover when we accounted for *T*_max_ distance (Supplementary Fig. 2, Supplementary Table 20 and Supplementary Appendix 4). Although further studies should investigate the relative contributions of different drivers of the inter- and intraspecific variation in responses to forest cover, these results suggest that proximity to species’ warm limits may be an important driver of latitudinal variation in responses to forest cover.

The impact of proximity to species’ warm limits on terrestrial endotherm responses to changes in forest cover, such as those that occur through land-use change, aligns with global efforts to understand terrestrial ectotherm responses to climatic warming^30^. Deutsch et al. (2008) showed that populations located at low latitudes are likely to be most negatively affected by warming, as they are currently located in environments near their warm limits, whereas populations at higher latitudes may be positively affected, as they are currently located in environments cooler than their warm limits and potentially cooler than their optimal temperatures^30^. This suggests that proximity to species’ warm limits mediates variation in how they respond to two major global change drivers.

### Integrating inter- and intraspecific variation into biodiversity models for a changing world

**Fig. 4:**
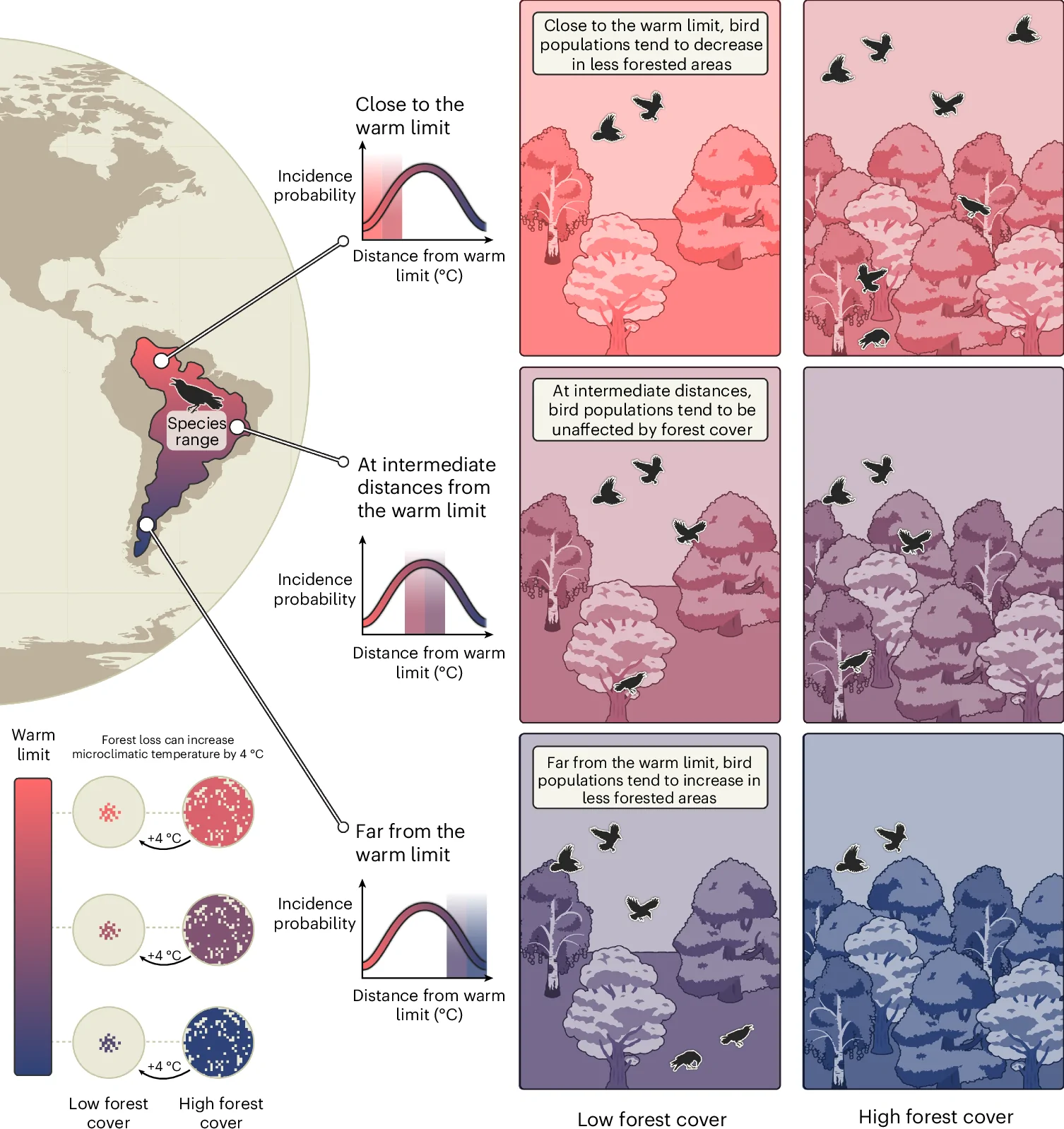
Proposed explanation of variation in bird responses to forest cover across a climatic gradient in a hypothetical species’ range. The colour hue shows the environmental temperature relative to the species’ realized warm tolerance limit; pink represents temperatures closer to the warm limit and blue represents temperatures further from the warm limit. Landscapes with low forest cover are on average 4 °C warmer than those with high forest cover (Fig. 3). The effect that this microclimatic temperature change has on bird populations depends on the climate, that is, the distance from the warm limit, in the region. The probability of incidence decreases in less forested areas close to the warm limit but is unaffected by forest cover at intermediate distances and can increase in less forested areas far from the warm limit (Fig. 3). Figure designed by Miranta Kouvari from Science Graphic Design.

Our results offer an empirically grounded framework for predicting inter- and intraspecific variation in how changes in forest cover may affect biodiversity under future climates. On average, forest loss brings populations 4 °C closer to species’ warm limits, negatively affecting populations in relatively warm regions of their species’ ranges but potentially benefiting those in regions that are cool relative to the species’ optimal temperatures (Figs. 3 and 4). This could explain why there is so much inter- and intraspecific variation in response to forest cover and has important implications for accurate projections of future biodiversity change. A population located at one position relative to its species’ warm limit (*T*_max_ distance) may not respond to deforestation in the same way as another population located at a different *T*_max_ distance, even though both populations may belong to the same species. Further, as populations are increasingly exposed to temperatures close to or beyond their species’ warm limits^69,70^, how a population responds to deforestation under past or current climatic conditions may not be indicative of how it will respond to deforestation at the same geographic location under future climatic conditions.

Incorporating population-level information on proximity to species’ warm limits into projections of the impact of land-use changes would allow models to move beyond assuming fixed species responses and to capture potential synergies between climate and land-use change. Although we have estimated proximity to species’ realized warm tolerance limits based on observed populations and geographic range limits here, integration of physiological data and biophysical models of individual heat budgets could provide more mechanistic insights into the processes governing biotic responses to the local climatic changes caused by land-use change. Such integration of landscape ecology, macroecology and mechanistic niche modelling would be a data-hungry and computationally intensive exercise, but it is an important next step in the development of more accurate projections of biodiversity change to inform both local and global conservation initiatives in a warmer human-modified world.

## Methods

### Bird community data

We compiled a large dataset consisting of studies of bird presence–absence (hereafter incidence) in response to changes in land use across six continents. Birds are an ideal study system for exploration of range-wide variation in responses to forest cover because their distributions are well characterized^71^ and there is a breadth of existing survey data of bird communities. We compiled studies on bird communities across landscapes of varying forest cover proportions. We use the term ‘site’ to refer to a georeferenced point location at which the sampling of a bird community took place. These sites were grouped into ‘studies’, in which sites within the same study were located close to each other, and were sampled using the same method by the same investigator during the same year(s).

The studies in our dataset were from three different databases, which we downloaded in January 2024. We chose these databases because the studies within them were designed to understand the effect of land-use change on ecological communities. The first of these was the PREDICTS Project database, which collates data from published studies of ecological assemblages across varying human-modified land uses^34^. The second was the BIOFRAG database, which collates primary datasets of ecological assemblages sampled at sites within fragmented forests^35^. To maximize the size of the overall dataset, we included studies from the PREDICTS or BIOFRAG databases that reported bird species abundance or incidence but converted the abundance data to incidence to ensure that we had a consistent response variable for our analyses. The third data source was the Atlantic Birds database, which reports bird species’ relative abundances (that is, numbers of individuals recorded per unit of sampling time) within studies across the Brazilian Atlantic Forest^36^. We converted this to incidence for each study; if a species was found at one site in a study but not at another site in the same study, we recorded that species as absent from the latter site. We also included four additional studies of bird abundance, which we converted to incidence, in the Atlantic Forest, sourced from relevant literature^21^.

As we were interested in the effects of forest cover, we removed studies conducted in non-forest ecoregions, defined according to the RESOLVE 2017 dataset^72^. We also removed species that did not use forests and only included species for which forest was listed as one of their habitat affiliations, according to the IUCN habitat classifications^38^. Our results were robust to this exclusion of non-forest species (Supplementary Appendix 5). To examine responses to forest cover, we used the global tree-cover dataset^39^ (see the ‘Forest cover’ section) which provides maps of tree cover in the year 2000. To ensure that bird incidence could have been influenced by the forest cover in 2000, we filtered our incidence dataset to include only studies that were conducted after the year 2000, or for which the timespan of the study included the year 2000. The timespans of studies are shown in Supplementary Fig. 19. If there were temporal duplicates of a study (that is, repeated surveys of the same locations in different years), we kept the study that was conducted in the year closest to 2000 to avoid pseudoreplication.

We aligned the species taxonomy to the BirdLife International checklist v.8.1^73^ using crosswalk tables from AVONET^74^ and OpenTree^75^. Presence observations >200 km outside species’ ranges are likely to be due to taxonomic errors. Therefore, we used the BirdLife range maps^76^ to filter out potential taxonomic issues. If a species was recorded as present >200 km outside its range in one study, we removed the species from that study. However, if a species was recorded as present >200 km outside its range in multiple studies, we removed that species from the analysis entirely.

In total, our global dataset included presence–absence data from 2,262 species sampled at 7,326 sites, collected from 111 studies conducted between 1996 and 2019, spanning six continents (Fig. 1 and Supplementary Table 1). Of the 111 studies, 70 were conducted using point counts, 17 using line transects, 14 with mist nets, 1 with visual encounter surveys, 1 by systematic searching and 8 using a combination of methods. Analysis of survey data compiled in large databases provides an opportunity to investigate inter- and intraspecific variation at global, macroecological scales. With this approach, it is not possible to adjust for imperfect detection in the underlying studies using methods such as occupancy models owing to the large number of species in the dataset^77,78^. We used random intercepts in statistical models to account for variation among studies. However, this approach does not correct for detection biases that may covary with forest cover.

### Forest cover

We calculated forest cover from a global tree-cover dataset^39^ that provided the approximate percentage of tree cover for each 30-m pixel in the year 2000. We converted this into a binary map distinguishing between forest and non-forest areas. Pixels with a tree-cover percentage exceeding 70% were classified as forest, whereas those with a percentage below 70% were considered to be non-forest. A study comparing forest cover estimates derived from different tree-cover thresholds in the dataset of Hansen et al.^39^ with ground truth data in Myanmar suggested that a 50% threshold achieved the highest accuracy in ecological zones other than tropical rainforests, whereas an 80% threshold was optimal for tropical rainforests^79^. Similarly, another study suggested an 80% threshold for assessment of forest cover within the Amazon basin^80^. Given that our dataset encompassed a variety of forest types, we opted for a 70% threshold. We conducted a sensitivity test using 50% and 80% tree-cover thresholds and found that varying this threshold did not affect our results (Supplementary Appendix 6).

We calculated the proportion of forest pixels within a 600-m buffer surrounding each site. This buffer distance matched the sampling design used in many of the original studies included in the dataset and therefore minimized spatial overlap among buffers. A 600-m buffer has also been shown to be optimal for assessing bird responses to forest cover^40–44^. Sensitivity analyses showed that use of alternative buffer sizes of 400 m or 800 m—scales that are also sometimes used in studies of forest cover effects on bird communities^81^—did not affect our results (Supplementary Appendix 7). Using this global tree-cover dataset provided a valuable opportunity to estimate forest cover worldwide using a standardized approach, thereby enabling global analyses of bird responses to forest cover. However, this measure did not capture variation in forest structure, nor did it distinguish between native forests and plantation forests.

### *T*_max_ distance

We calculated the average daily maximum temperature of the warmest month, which corresponds to the official BIOCLIM variable BIO05. The full definition of BIO05 is the ‘maximum daily temperature averaged over the month with the highest monthly mean of daily maximum temperature’^82^. To calculate BIO05, we first obtained global hourly temperature data at 2 m above ground in the year 2000 at 0.25° gridded spatial resolution from ERA5^50^. For each pixel, we (1) identified the hottest temperature of each day, (2) calculated the average of these daily temperatures for each month and (3) selected the highest of these monthly average temperatures. We therefore identified the hottest month and the average daily maximum temperature of that month in each pixel. The BIO05 temperature in the year 2000 at our study sites was closely related to the longer-term 40-year average BIO05 temperature at these sites (Supplementary Appendix 9). We estimated the realized warm tolerance limit for each species by overlaying these maximum temperature maps with expert-verified geographic range maps from BirdLife^76^. For each species, the realized warm tolerance limit (referred to as the ‘warm limit’ or *T*_max_) is the highest maximum temperature within its geographic range^27^. The realized distribution reflects the set of environmental conditions under which a species currently persists. At temperatures beyond the realized warm tolerance limit, there is a lack of evidence that the species can persist in the wild^49,70^. We used realized warm tolerance limits inferred from geographic distributions rather than physiological tolerance limits derived from laboratory experiments; this was because physiological data are scarce, particularly for vertebrates; often lack comparability across studies; and may not accurately represent conditions experienced in natural environments^83–85^. Therefore, we inferred species’ realized warm limits from their geographic range maps. This provided a standardized approach that could be generalized across the 2,262 species in our study. These warm limits represent the upper envelope of regional macroclimatic conditions associated with the mapped range. To test the robustness of our results against potential temperature outliers within species’ ranges or potential use of cooler microhabitats by species within the hottest pixel of their geographic range, we also calculated the warm limits using the 95th percentile of the hottest monthly temperature within each species’ range rather than the maximum; we found that this did not affect our results (Supplementary Appendix 8).

When estimating each species’ realized warm limit, we retained range polygons with an origin category of ‘native’ or ‘reintroduced’ and a presence status of ‘extant’ or ‘possibly extant’. For migratory species, we included both migratory and non-migratory ranges, as we were interested in estimating the hottest realized temperature associated with locations in which the species is known or likely to exist. We found that both migratory and non-migratory species showed similar effects of *T*_max_ distance on responses to forest cover (Extended Data Fig. 4 and Supplementary Table 10), so we included migratory species in our main analyses, as in ref. ^86^.

We also extracted the maximum temperature of the warmest month in 2000 at each site and used the difference between the maximum temperature of a site and the species’ realized warm limit (*T*_max_ distance) to investigate how the species-specific macroclimate temperatures in which a population was located might affect its response to forest cover (Fig. 2). Although the studies in our dataset were conducted at various times of the year, we used the maximum temperature of the warmest month (BIO05) as a key climatic variable, because maximum temperatures can have lasting ecological effects beyond the period in which they occur^63,87^.

### Species’ traits

We investigated whether species’ traits could explain how much their *T*_max_ distance affected their response to forest cover. We chose the following traits because they have been shown to affect species sensitivity to forest cover or temperature: geographic range size, primary habitat preference, nest type, migration, body mass, hand-wing index, primary foraging height and primary diet. Specifically, species with smaller ranges have been shown to be more likely to have greater sensitivity to forest cover^53^. Species with forest as their primary habitat preference would be expected to be more sensitive to forest cover^7^. Nest type can affect responses to temperature, as open nests tend to be less thermally buffered than closed nests^52^. Body mass could also affect responses to temperature, as climatic warming tends to favour smaller-bodied species^54^. Species with lower dispersal abilities (as measured by the hand-wing index) have been shown to be more sensitive to changes in forest cover, as they are less able to cross gaps between habitat patches^8^. Foraging height and diet can also affect sensitivity, with understorey species and insectivorous species often showing greater sensitivity to forest fragmentation^51^. We also included an analysis of migratory versus non-migratory species to test whether the inclusion of migratory species in our main analyses affected the temperature mediation of responses to forest cover.

We obtained data on species’ migration, body mass, hand-wing index, primary habitat preference and geographic range size from AVONET^74^. We obtained data on species’ nest type from the global database of bird nest traits^88^ and grouped these into ‘open’ and ‘closed’ nest types. We used data on species’ diet from EltonTraits^89^. There were five diet categories, based on species’ dominant food source: (1) plants and seeds; (2) fruits and nectar; (3) invertebrates; (4) vertebrates, fish and carrion; and (5) more than one of the above categories^89^. We also used data on species’ foraging height from EltonTraits^89^. We categorized each species into one foraging category according to the category it used the most and analysed the four most commonly occurring foraging categories; these were (1) ground, (2) understorey, (3) mid-height and (4) canopy, where ‘mid-height’ refers to foraging above 2 m in trees or high bushes but below canopy.

### Microclimate model

To investigate how local microclimates might drive the macroclimatic variation in bird responses to forest cover, we used the microclimc model^57^ in R v.4.3.1. This is a mechanistic framework based on first principles of physics that predicts heat exchange processes both above and below the forest canopy. We modelled the microclimate temperatures for each site (georeferenced point location) at 2 m above ground in hourly timesteps for the hottest month at that site in 2000. The effects of *T*_max_ distance on responses to forest cover were consistent regardless of the primary foraging height of the species (Extended Data Fig. 4 and Supplementary Table 13); therefore, we modelled the temperatures at a fixed height of 2 m above ground for all species. We chose the year 2000 as it aligned with the forest cover data. As the effects of warm temperatures can persist over time^87^, and the studies in our dataset were conducted during or after 2000, we expected species in these studies to have been influenced by the temperature in 2000. Although studies conducted in later years may have experienced different absolute temperatures to those in 2000, the relative difference in temperature between forested and non-forested sites was not expected to change; this allowed us to assess how temperature affected responses to forest cover.

We obtained hourly climate input data for the microclimate model from the ERA5 global climate reanalysis, using the single-levels surface dataset with a spatial resolution of 0.25° (ref. ^50^). For each study area, we used the mcera5 R package^90^ to extract the following climate variables: (1) air temperature at 2 m, (2) dewpoint temperature at 2 m, (3) surface pressure, (4) U-wind speed at 10 m (west to east component), (5) V-wind speed at 10 m (south to north component), (6) total precipitation, (7) total cloud cover, (8) mean surface net long-wave radiation flux, (9) mean surface downward long-wave radiation flux, (10) total sky direct solar radiation at surface and (11) surface solar radiation downwards.

We incorporated the following environmental predictors into the microclimate model: (1) yearly habitat type from MODIS at 500-m resolution for the year 2001^91^; and (2) monthly LAI from MODIS at 500-m resolution for 2001^92^. We used the LAI data for the hottest month that we identified in the year 2000, as this aligned with the timespan of our microclimate models. Habitat type and LAI data at this resolution were not available before 2001 but we expected the habitat type and LAI in 2001 to be good approximations of these variables in 2000. (3) Forest canopy height at 30-m resolution derived from Landsat data^93^ for the year 2000; (4) soil texture class at 250-m resolution and 0-m soil depth from the USDA classification^94^; and (5) elevation at 30-m resolution from AWS terrain tiles^95^.

### Microclimate model validation

We validated the outputs of the microclimate model using empirical microclimate temperature records from four datasets. To do this, we compared hourly modelled microclimate temperatures with above-ground in situ temperature measurements from four independently sourced datasets. Two of these datasets were from separate studies conducted in the Brazilian Atlantic Forest. The first dataset was collected between September 2009 and August 2010 and used iButton DS1922L-F5 loggers placed 1 m above ground; the second dataset was collected between October 2014 and December 2014 and used NOVUS model LOG BOX-RHT-LCD loggers 1 m above ground^96^; the third dataset came from the Stability of Altered Forest Ecosystems project in lowland tropical forests in Borneo, where microclimate temperatures were recorded between September 2011 and May 2012 using Hygrochron iButton loggers at 1 m above ground^97,98^; and the fourth dataset was collected from temperate forests in 2009 at the H. J. Andrews Experimental Forest in Oregon using HOBO Pendant loggers 1.5 m above ground^99^.

We modelled the microclimate of the hottest month for which in situ temperature measurements were available. For each site, we used the modelling method described above but modelled the microclimate at the same height above ground at which the loggers were placed for each dataset (1 m for the Atlantic Forest and Borneo datasets, and 1.5 m for the Oregon dataset). For the microclimate model input, we used vegetation and ERA5 climate variables that matched the timespan of the recorded temperatures, although we used canopy height from the year 2000 as yearly canopy height data were not available with the required spatial extent and resolution.

We used two complementary approaches to assess the performance of the microclimate model. First, we compared the overall agreement between empirical and modelled hourly temperatures by fitting a linear regression to each of the four datasets and calculating the root mean square error and *R*^2^. Association could arise owing to similarities in macroclimate between these two variables. Therefore, we also conducted a more stringent test to assess how well the model captured deviations from macroclimate conditions. For this, we calculated the difference between the modelled microclimate temperature and the ERA5 macroclimate temperature at each site; similarly, we calculated the difference between the empirical temperature recorded by in situ loggers and the ERA5 macroclimate temperature. We then fitted a linear regression between these two sets of differences and calculated the root mean square error and *R*^2^ (Supplementary Appendix 10). Although these approaches provide estimates of microclimate model performance, any differences between recorded and modelled microclimate temperatures could have several causes, including known inaccuracies of in situ temperature loggers^100^.

### Micro*T*_max_ distance

We identified the daily maximum temperatures from the hourly output of the microclimate model at each site. We then calculated the mean of these daily maximum temperatures over the hottest month in the year 2000. This gave a microclimate analogue of the BIO05 variable. For each site-by-species combination, we calculated the temperature difference (in °C) between this microclimate temperature at the sample site and the species’ warm limit (micro*T*_max_ distance). We used this to investigate how local microclimates might drive responses to forest cover (Fig. 3).

### Statistical analysis

To investigate how bird incidence was affected by *T*_max_ distance and forest cover, we first used a binomial GLMM with a logit link function. This model included forest cover proportion, *T*_max_ distance and their interaction as fixed-effect predictors of incidence (Fig. 2a). Before model fitting, we confirmed that forest cover proportion and *T*_max_ distance were not correlated (Pearson’s correlation coefficient = −0.078, 95% CI −0.081, −0.075). To account for variation among studies, we included a nested random intercept for site within study (1 | study/site). The models also incorporated random slopes for each species that allowed the effects of forest cover, *T*_max_ distance and their interaction to vary among species. We also used random intercepts to account for variation in baseline incidence among species. The species-level random effects were (1 + forest cover + *T*_max_ distance + forest cover:*T*_max_ distance | species) (Supplementary Table 2). To test for phylogenetic correlation in the species-level random effects, we used phylogenetic generalized least squares to estimate *λ*, a measure of phylogenetic signal. Phylogenetic signal was consistently low (*λ* *=* 0.029–0.084). Consequently, phylogeny was not included in the main analytical models (Supplementary Appendix 3). As a sensitivity test, we fitted a phylogenetic GLMM in which phylogeny was incorporated as a structured random effect. The inclusion of phylogeny did not affect our results (Supplementary Appendix 3).

To assess whether the effects of *T*_max_ distance, forest cover and their interaction on incidence varied across biogeographic realms (Fig. 2b and Supplementary Table 3), we fitted a single model that included a three-way interaction among forest cover, *T*_max_ distance and biogeographic realm, along with all lower-order two-way interactions and main effects. This model used the same random effects as those described above. We also fitted a similar model with biome instead of realm to assess whether the effects of *T*_max_ distance, forest cover and their interaction on incidence varied across biomes (Extended Data Fig. 2 and Supplementary Table 4). As *T*_max_ distance was correlated with latitude (correlation coefficient = 0.47), and latitude can influence responses to forest cover^8,10,68^, we also fitted a model with *T*_max_ distance, forest cover and their interaction, as well as latitude and its interaction with forest cover, to test whether accounting for latitude would alter the effect of *T*_max_ distance on responses to forest cover (Supplementary Fig. 2, Supplementary Table 20 and Supplementary Appendix 4).

To analyse variation in the responses to forest cover both across all species and within each species, we took the model derivative with respect to forest cover for the fixed effects and species-specific random effects, respectively^20^ (Fig. 2c). A positive value of this derivative indicated an increase in incidence with increasing forest cover, whereas a negative value indicated a decrease in incidence with increasing forest cover. Taking the derivative with respect to forest cover for the fixed-effects model estimates provided a metric of sensitivity to forest cover at each *T*_max_ distance, averaged across all species. Application of this calculation to the species-specific random effects provided a separate estimate for each species of its sensitivity to forest cover at each *T*_max_ distance^20^.

We visualized both the overall fixed-effects derivatives and the species-specific random-effect derivatives to characterize how the overall average response to forest cover varied with *T*_max_ distance and to illustrate the heterogeneity in these patterns among species (Fig. 2c and Extended Data Figs. 3 and 4). To summarize the direction and relative magnitude of the association between *T*_max_ distance and response to forest cover within each species, we fitted a separate linear regression for each species, with *T*_max_ distance as the predictor and the derivative with respect to forest cover as the response. These regressions were used solely as descriptive summaries of the direction and relative magnitude of species-specific trends. Formal statistical inference was not assessed because *T*_max_ distance served both as the predictor in these regressions and as an input to the derivative calculations, resulting in no residual variance around the fitted relationships.

To investigate whether species’ traits modulated the effect of *T*_max_ distance on responses to forest cover, we fitted a suite of GLMMs, each incorporating a different trait as an additional fixed effect. We tested the following traits individually in separate models: geographic range size, primary habitat preference (forest or non-forest), nest type, migration type, body mass, hand-wing index, primary diet type and primary foraging height. In each model, the fixed effects were forest cover, *T*_max_ distance and the trait of interest, along with their interactions (including a three-way interaction among these predictors, as well as all corresponding lower-order two-way interactions).

Previous global assessments of the thermal buffering effect have found that the magnitude of the buffering effect was stronger in tropical forests than temperate forests^29^. Therefore, to investigate how forests buffer warm microclimate temperatures, and how this might vary across biogeographic realms, we used a linear mixed effect model with forest cover and biogeographic realm as interacting fixed-effect predictors (Extended Data Fig. 5 and Supplementary Table 15). The response variable was the average of the hottest daily temperatures across the hottest month (a microclimate analogue of the BIO05 variable). We used the emmeans package^101^ to compute the slopes for each realm. The model also included a random slope for each study. We fitted a similar model with biome instead of realm to assess how the microclimate buffering effect of forest might vary across biomes (Extended Data Fig. 5 and Supplementary Table 16).

To quantify the extent to which declining forest cover pushed populations closer to their species’ warm limit, we fitted a linear mixed effect model with forest cover as a predictor of *T*_max_ distance. This model included a random slope and intercept for each species (Fig. 3a). To investigate how micro*T*_max_ distance affected the probability of species’ incidence, we used a binomial GLMM with a quadratic term for micro*T*_max_ distance. The random effects were a random intercept for study and random intercepts and slopes to account for differences in the fixed effects across species (Supplementary Table 6). We also fitted a similar model without the quadratic term (using only micro*T*_max_ distance as the fixed-effect predictor) and compared the Akaike information criterion (AIC) of this model with that of the model incorporating the quadratic term. Incorporating a quadratic term into this model improved the AIC (ΔAIC = 2,221). The assumptions underlying the statistical models were checked using the DHARMa package^102^. Unless otherwise stated, all analyses were conducted in R v.4.4.1. All R scripts used for analysis are available via figshare^103^.

### Reporting summary

Further information on research design is available in the Nature Portfolio Reporting Summary linked to this article.

## Supplementary information

Supplementary Information Supplementary Appendices 1–11, including Figs. 1–19 and Tables 1–34.

Reporting Summary

Peer Review File

## Data Availability

The PREDICTS database can be downloaded from https://data.nhm.ac.uk/dataset/the-2016-release-of-the-predicts-database-v1-1 and https://data.nhm.ac.uk/dataset/release-of-data-added-to-the-predicts-database-november-2022. The BIOFRAG data can be requested from https://biofrag.wordpress.com/biofrag-measuring-biodiversity-response-to-forest-fragmentation/. The Atlantic Bird data can be downloaded from https://doi.org/10.1002/ecy.2119, the global tree-cover maps used for estimation of forest cover from https://storage.googleapis.com/earthenginepartners-hansen/GFC-2022-v1.10/download.html and the hourly temperature maps used for calculating *T*_max_ distance from https://cds.climate.copernicus.eu/datasets/reanalysis-era5-single-levels?tab=download. The species’ range maps used for estimating species’ warm can be requested from BirdLife International via https://datazone.birdlife.org/contact-us/request-our-data. The hourly climate data used to drive the microclimate model can be downloaded from https://cds.climate.copernicus.eu/datasets/reanalysis-era5-single-levels?tab=download. The environmental parameters used in the microclimate model included: (1) LAI (https://lpdaac.usgs.gov/products/mod15a2hv006/), (2) canopy height (https://glad.umd.edu/dataset/GLCLUC2020), (3) habitat types (https://lpdaac.usgs.gov/products/mcd12q1v061/), (4) digital elevation (https://registry.opendata.aws/terrain-tiles/) and (5) soil types (https://doi.org/10.5281/zenodo.2525817). The R scripts and data generated in this study are available via figshare at https://doi.org/10.6084/m9.figshare.32736735 (ref. ^103^).

## Code availability

The R scripts are available via figshare at https://doi.org/10.6084/m9.figshare.32736735 (ref. ^103^).
