# Supplementary material for: Temperature mediates variation in avian sensitivity to forest cover
Source: Nat Ecol Evol. 2026 Aug 28;10(10):1914–26. doi: 10.1038/s41559-026-03160-9 (PMC13642040; doi:10.1038/s41559-026-03160-9)
Supplement: Supplementary file 1 [file 41559_2026_3160_MOESM1_ESM.pdf]

nature ecology & evolution

Supplementary information https://doi.org/10.1038/s41559-026-03160-9

Temperature mediates variation in avian sensitivity to forest cover

In the format provided by the authors and unedited

Contents

Appendix 1: List of studies 2
Appendix 2: Model coefficients 5
Appendix 3: Testing for and accounting for phylogenetic non-independence among species 20
Appendix 4: Accounting for latitude 22
Appendix 5: Sensitivity test for including non-forest species 26
Appendix 6: Sensitivity test for tree cover thresholds used to calculate forest cover proportions in the landscape surrounding each site 30
Appendix 7: Sensitivity test for buffer sizes used to calculate forest cover proportions in the landscape surrounding each site 37
Appendix 8: Sensitivity test for calculating T_{max} using the 95th percentile of the maximum monthly temperatures experienced within the species' range 44
Appendix 9: Comparison of maximum temperature of the warmest month (*BIO05*) in the year 2000 with long-term climate average 48
Appendix 10: Microclimate model validation 50
Appendix 11: Timespan of studies 52
References 53

Appendix 1: List of studies

Table S1. List of studies.

	Database	Author	Year	Realm	Method	Number of sites	Number of species surveyed
**1**	BIOFRAG	Phalan	2007	Afrotropical	Point count	24	145
**2**	BIOFRAG	Marsh	2008	Afrotropical	Point count	43	21
**3**	BIOFRAG	Banks-Leite	2005-2007	Neotropical	Mist net	17	117
**4**	BIOFRAG	Banks-Leite	2005-2007	Neotropical	Mist net	21	118
**5**	BIOFRAG	Banks-Leite	2005-2007	Neotropical	Mist net	19	118
**6**	BIOFRAG	Stouffer	2007	Neotropical	Mist net	11	102
**7**	BIOFRAG	Wood	2007-2009	Nearctic	Point count	238	63
**8**	BIOFRAG	Cerezo	1998-2001	Neotropical	Point count	118	171
**9**	BIOFRAG	Richard	2001-2006	Australasian	Point count	48	45
**10**	BIOFRAG	Kormann	2008-2012	Neotropical	Point count	40	147
**11**	BIOFRAG	Barlow	2005	Neotropical	Mist net	15	207
**12**	BIOFRAG	Betts	2000-2002	Nearctic	Point count	741	27
**13**	BIOFRAG	Arroyo-Rodriguez	2010-2012	Neotropical	Point count	20	63
**14**	BIOFRAG	Possingham	2001	Australasian	Point count	164	64
**15**	BIOFRAG	Morante-Filho	2013-2014	Neotropical	Point count	40	153
**16**	BIOFRAG	Olivier	2012-2015	Afrotropical	Point count	721	106
**17**	BIOFRAG	Barbaro	2011	Palearctic	Point count	12	19
**18**	BIOFRAG	Lasky	2005	Neotropical	Point count	111	49
**19**	BIOFRAG	Karubian	2014-2016	Neotropical	Mist net	32	94
**20**	BIOFRAG	Karubian	2004	Neotropical	Mist net	15	87
**21**	BIOFRAG	Proença	2005	Palearctic	Point count	26	30
**22**	BIOFRAG	Raman	2002-2003	Indo-Malayan	Point count	13	87
**23**	BIOFRAG	Jactel	2012-2013	Palearctic	Various	28	31
**24**	BIOFRAG	Jactel	2012-2013	Palearctic	Various	38	29
**25**	BIOFRAG	Jactel	2012-2013	Palearctic	Various	36	17
**26**	BIOFRAG	Jactel	2012-2013	Palearctic	Various	43	24
**27**	BIOFRAG	Jactel	2012-2013	Palearctic	Various	28	26
**28**	BIOFRAG	Jactel	2012-2013	Palearctic	Various	35	23
**29**	BIOFRAG	Terraube	2011	Palearctic	Line transect	32	30
**30**	BIOFRAG	Terraube	2011	Palearctic	Line transect	21	14
**31**	BIOFRAG	Terraube	2011	Palearctic	Line transect	22	29
**32**	Addition	Hatfield	2015-2017	Neotropical	Point count	24	171
**33**	Addition	dosAnjos	1997-2000	Neotropical	Point count	14	158
**34**	Addition	Uezu	2002-2004	Neotropical	Point count	28	90
**35**	Addition	Develey	2000-2003	Neotropical	Point count	31	144
**36**	ATLANTIC	Alexandrino	2011-2012	Neotropical	Point count	8	128
**37**	ATLANTIC	Athiê	2007-2009	Neotropical	Point count	2	69
**38**	ATLANTIC	Coelho	2007-2011	Neotropical	Point count	6	72
**39**	ATLANTIC	Gussoni	2006-2007	Neotropical	Point count	2	117

**40**	ATLANTIC	Lobo-Araújo	2003-2008	Neotropical	Point count	2	104
**41**	ATLANTIC	Longo	2005-2006	Neotropical	Mist net	2	30
**42**	ATLANTIC	Luz	2011-2012	Neotropical	Mist net	8	62
**43**	ATLANTIC	Piratelli	2000-2001	Neotropical	Mist net	4	5
**44**	ATLANTIC	Ribon	1996-2000	Neotropical	Point count	39	59
**45**	ATLANTIC	Roos	2001	Neotropical	Mist net	5	41
**46**	ATLANTIC	Sebaio	2000-2001	Neotropical	Mist net	5	68
**47**	ATLANTIC	Silveira	2001	Neotropical	Line transect	14	146
**48**	PREDICTS	Alvarez	2014-2015	Neotropical	Point count	40	43
**49**	PREDICTS	Azhar	2009	Indo-Malayan	Line transect	74	146
**50**	PREDICTS	Azman	2009	Indo-Malayan	Various	3	89
**51**	PREDICTS	Bartolommei	2008	Palearctic	Point count	122	4
**52**	PREDICTS	Barzan	2012-2013	Neotropical	Point count	59	96
**53**	PREDICTS	Borges	2003	Neotropical	Visual encounter survey	12	107
**54**	PREDICTS	Brandt	2010	Palearctic	Point count	61	58
**55**	PREDICTS	Báldi	2003	Palearctic	Line transect	42	35
**56**	PREDICTS	Cabral	2017-2019	Neotropical	Point count	8	88
**57**	PREDICTS	Chapman	2000	Nearctic	Point count	228	67
**58**	PREDICTS	Chiawo	2012-2013	Afrotropical	Point count	3	64
**59**	PREDICTS	Cockle	2001-2002	Neotropical	Line transect	2	163
**60**	PREDICTS	Dayananda	2015-2018	Indo-Malayan	Point count	32	37
**61**	PREDICTS	Doulton	2005	Afrotropical	Point count	121	20
**62**	PREDICTS	Dures	2008-2009	Afrotropical	Point count	29	27
**63**	PREDICTS	Edenius	2010	Palearctic	Point count	29	22
**64**	PREDICTS	Farwig	2005-2006	Afrotropical	Point count	15	94
**65**	PREDICTS	Flaspohler	2006	Australasian	Systematic searching	2	6
**66**	PREDICTS	Gomes	2003-2004	Neotropical	Line transect	9	74
**67**	PREDICTS	Gove	2007	Afrotropical	Point count	38	57
**68**	PREDICTS	Hassan	2010-2011	Afrotropical	Point count	32	77
**69**	PREDICTS	Hayward	2003	Afrotropical	Line transect	84	15
**70**	PREDICTS	Kajtoch	2014	Palearctic	Point count	66	36
**71**	PREDICTS	Karp	2016	Neotropical	Point count	118	75
**72**	PREDICTS	Kessler	2007	Australasian	Various	15	64
**73**	PREDICTS	Kitazawa	2015-2016	Palearctic	Line transect	15	12
**74**	PREDICTS	Latta	2006-2007	Neotropical	Point count	2	7
**75**	PREDICTS	Le	2018	Palearctic	Point count	100	37
**76**	PREDICTS	Mallari	2006	Indo-Malayan	Line transect	65	45
**77**	PREDICTS	Mandal	2014	Indo-Malayan	Line transect	100	67
**78**	PREDICTS	Martin	2017-2018	Afrotropical	Point count	80	12
**79**	PREDICTS	Milder	2008-2009	Neotropical	Point count	79	119
**80**	PREDICTS	Miranda	2006-2007	Neotropical	Mist net	2	22
**81**	PREDICTS	Moreno	2005-2007	Palearctic	Point count	65	2
**82**	PREDICTS	Morgado	2014-2016	Palearctic	Point count	191	16
**83**	PREDICTS	Munyekenye	2004-2005	Afrotropical	Point count	208	106
**84**	PREDICTS	Munyekenye	2005	Afrotropical	Point count	64	106

**85**	PREDICTS	Naithani	2005-2007	Indo-Malayan	Line transect	6	67
**86**	PREDICTS	Ndang	2010-2011	Afrotropical	Point count	334	39
**87**	PREDICTS	O'Dea	2003	Neotropical	Point count	150	169
**88**	PREDICTS	Otto	2010	Nearctic	Point count	143	19
**89**	PREDICTS	Otto	2011	Nearctic	Point count	79	19
**90**	PREDICTS	Palomino	2001-2003	Palearctic	Point count	277	47
**91**	PREDICTS	Politi	2005-2006	Neotropical	Point count	4	83
**92**	PREDICTS	Ranganathan	2002	Neotropical	Point count	67	120
**93**	PREDICTS	Ranganathan	2004	Indo-Malayan	Line transect	30	79
**94**	PREDICTS	Reid	2007	Neotropical	Point count	27	139
**95**	PREDICTS	Rey	2008	Palearctic	Point count	95	33
**96**	PREDICTS	Rey	2009	Palearctic	Point count	111	33
**97**	PREDICTS	Rosselli	2009-2010	Neotropical	Point count	12	46
**98**	PREDICTS	Sam	2010-2011	Australasian	Point count	4	92
**99**	PREDICTS	Santana	2005-2006	Palearctic	Point count	48	32
**100**	PREDICTS	Shahabuddin	2002	Indo-Malayan	Point count	30	39
**101**	PREDICTS	Sheldon	2005	Indo-Malayan	Point count	33	107
**102**	PREDICTS	Song	2016	Palearctic	Line transect	16	34
**103**	PREDICTS	St-Laurent	2004	Nearctic	Point count	60	19
**104**	PREDICTS	Suarez	2004-2005	Neotropical	Point count	23	41
**105**	PREDICTS	Suarez	2006	Nearctic	Point count	6	32
**106**	PREDICTS	Tanalgo	2010	Indo-Malayan	Mist net	4	25
**107**	PREDICTS	Van	2004	Neotropical	Point count	198	135
**108**	PREDICTS	Wang	2006	Palearctic	Line transect	5	58
**109**	PREDICTS	Yamaura	2009	Palearctic	Line transect	6	34
**110**	PREDICTS	Zhang	2017-2018	Palearctic	Point count	33	68
**111**	PREDICTS	de Lima	2009-2010	Afrotropical	Point count	292	22

Appendix 2: Model coefficients

Table S2. Binomial GLMM results for model testing how T_{max} distance, forest cover and their interaction affect probability of incidence. *In the model formula, the * operator is a shorthand for including all main effects and their interactions, so Incidence ~ T_{max} distance * forest cover is equivalent to Incidence ~ Tmax distance + forest cover + T_{max} distance: forest cover.*

**Incidence ~ T_{max} distance * forest cover + (1|Study / Site) + (T_{max} distance * forest cover | Species)**				
Predictors	*Log-Odds*	*std. Error*	*z*	*p*
T_{max} distance	0.090	0.015	6.156	**<0.001**
Forest cover proportion	3.188	0.188	16.913	**<0.001**
Interaction between T_{max} distance and forest cover	-0.201	0.017	-12.202	**<0.001**
N_{Site}	7326			
N_{Study}	111			
$N_{Species}$	2262			
Observations	467333			
Marginal R^2 / Conditional R^2	0.02 / 0.72			
AIC	294910			

Appendix 2: Model coefficients

Table S3. Binomial GLMM results for model testing how T_{max} distance, forest cover, biogeographic realm and their interactions affect probability of incidence.

Incidence ~ T_{max} distance * forest cover * biogeographic realm + (1|Study / Site) + (T_{max} distance * forest cover | Species)

Predictors	*Log-Odds*	*std. Error*	*z*	*p*
T_{max} distance [reference level = Afrotropical]	-0.041	0.021	-1.959	0.050
Forest cover proportion [reference level = Afrotropical]	1.954	0.349	5.593	**<0.001**
Interaction between T_{max} distance and forest cover proportion [reference level = Afrotropical]	-0.177	0.028	-6.246	**<0.001**
Interaction between T_{max} distance and realm [Indo-Malayan]	0.237	0.033	7.148	**<0.001**
Interaction between T_{max} distance and realm [Nearctic]	0.140	0.054	2.588	**0.010**
Interaction between T_{max} distance and realm [Neotropical]	0.264	0.026	10.191	**<0.001**
Interaction between T_{max} distance and realm [Australasian]	0.060	0.055	1.092	0.275
Interaction between T_{max} distance and realm [Palearctic]	0.145	0.028	5.196	**<0.001**
Interaction between forest cover proportion and realm [Indo-Malayan]	1.361	0.562	2.423	**0.015**
Interaction between forest cover proportion and realm [Nearctic]	4.587	1.025	4.474	**<0.001**
Interaction between forest cover proportion and realm [Neotropical]	2.317	0.428	5.412	**<0.001**
Interaction between forest cover proportion and realm [Australasian]	-0.598	0.811	-0.737	0.461
Interaction between forest cover proportion and realm [Palearctic]	2.967	0.686	4.327	**<0.001**
Interaction between T_{max} distance, forest cover proportion and realm [Indo-Malayan]	-0.028	0.045	-0.628	0.530
Interaction between T_{max} distance, forest cover proportion and realm [Nearctic]	-0.178	0.069	-2.599	**0.009**
Interaction between T_{max} distance, forest cover proportion and realm [Neotropical]	-0.124	0.036	-3.419	**0.001**

Interaction between T_{max} distance, forest cover proportion and realm [Australasian]	0.027	0.073	0.373	0.709
Interaction between T_{max} distance, forest cover proportion and realm [Palearctic]	-0.085	0.039	-2.182	**0.029**
N_{Site}	7326			
N_{Study}	111			
$N_{Species}$	2262			
Observations	467333			
Marginal R^2 / Conditional R^2	0.05 / 0.72			
AIC	294614			

Table S4. Binomial GLMM results for model testing how T_{max} distance, forest cover, biome and their interactions affect probability of incidence.

Incidence ~ T_{max} distance * forest cover * biome + (1|Study / Site) + (T_{max} distance * forest cover | Species)

Predictors	*Log-Odds*	*std. Error*	*z*	*p*
T_{max} distance [reference level = Mediterranean Forests, Woodlands & Scrub]	0.090	0.024	3.758	**<0.001**
Forest cover proportion [reference level = Mediterranean Forests, Woodlands & Scrub]	3.421	0.547	6.251	**<0.001**
Interaction between T_{max} distance and forest cover proportion [reference level = Mediterranean Forests, Woodlands & Scrub]	-0.205	0.035	-5.806	**<0.001**
Interaction between T_{max} distance and biome [reference level = Temperate Broadleaf & Mixed Forests]	0.001	0.022	0.058	0.954
Interaction between T_{max} distance and biome [reference level = Temperate Conifer Forests]	0.069	0.032	2.183	0.029
Interaction between T_{max} distance and biome [reference level = Tropical & Subtropical Coniferous Forests]	0.049	0.039	1.272	0.203
Interaction between T_{max} distance and biome [reference level = Tropical & Subtropical Dry Broadleaf Forests]	0.172	0.042	4.118	**<0.001**
Interaction between T_{max} distance and biome [reference level = Tropical & Subtropical Moist Broadleaf Forests]	0.008	0.023	0.362	0.717
Interaction between forest cover proportion and biome [reference level = Temperate Broadleaf & Mixed Forests]	1.145	0.655	1.749	0.080
Interaction between forest cover proportion and biome [reference level = Temperate Conifer Forests]	5.637	1.023	5.513	**<0.001**
Interaction between forest cover proportion and biome [reference level = Tropical & Subtropical Coniferous Forests]	-1.015	1.148	-0.884	0.377

Interaction between forest cover proportion and biome [reference level = Tropical & Subtropical Dry Broadleaf Forests]	2.789	0.837	3.333	**0.001**
Interaction between forest cover proportion and biome [reference level = Tropical & Subtropical Moist Broadleaf Forests]	-0.404	0.556	-0.726	0.468
Interaction between T_{max} distance, forest cover proportion and biome [reference level = Temperate Broadleaf & Mixed Forests]	-0.059	0.036	-1.659	0.097
Interaction between T_{max} distance, forest cover proportion and biome [reference level = Temperate Conifer Forests]	-0.203	0.046	-4.427	**<0.001**
Interaction between T_{max} distance, forest cover proportion and biome [reference level = Tropical & Subtropical Coniferous Forests]	0.053	0.077	0.692	0.489
Interaction between T_{max} distance, forest cover proportion and biome [reference level = Tropical & Subtropical Dry Broadleaf Forests]	-0.321	0.084	-3.836	**<0.001**
Interaction between T_{max} distance, forest cover proportion and biome [reference level = Tropical & Subtropical Moist Broadleaf Forests]	-0.009	0.035	-0.260	0.795
N_{Site}	7238			
N_{Study}	109			
$N_{Species}$	2256			
Observations	465325			
Marginal R^2 / Conditional R^2	0.03 / 0.72			

Appendix 2: Model coefficients

Table S5. Linear mixed effect model results for model testing how forest cover affects microT$_{max}$ distance.

**microT$_{max}$ distance ~ Forest cover proportion + (Forest cover proportion | Species)**				
Predictors	*Estimates*	*std. Error*	*z*	*p*
Forest cover proportion	4.660	0.088	52.845	**<0.001**
N $_{Species}$	2262			
Observations	467333			
Marginal R^2 / Conditional R^2	0.08 / 0.84			

Table S6. Binomial GLMM results for model testing how microT$_{max}$ distance affects probability of incidence.

**Incidence ~ microT$_{max}$ distance^2 + (1 |Study/Site) + (microT$_{max}$ distance^2 | Species)**				
Predictors	*Log-Odds*	*std. Error*	*z*	*p*
microT$_{max}$ distance [1st degree]	0.400	0.025	15.935	**<0.001**
microT$_{max}$ distance [2nd degree]	-0.013	0.001	-14.362	**<0.001**
N $_{Site}$	7326			
N $_{Study}$	111			
N $_{Species}$	2262			
Observations	467333			
Marginal R^2 / Conditional R^2	0.04 / 0.71			
AIC	299752			

Appendix 2: Model coefficients

Table S7. Binomial GLMM results for model testing how T_{max} distance, forest cover, species' geographic range area (log-transformed) and their interactions affect probability of incidence.

Incidence ~ T_{max} distance * forest cover * log(range size) + (1|Study / Site) + (T_{max} distance * forest cover | Species)

Predictors	*Log-Odds*	*std. Error*	*z*	*p*
T_{max} distance	0.108	0.105	1.028	0.304
Forest cover proportion	4.295	1.080	3.976	**<0.001**
Interaction between T_{max} distance and forest cover	0.158	0.119	1.328	0.184
Interaction between T_{max} distance and log(range size)	-0.003	0.007	-0.401	0.689
Interaction between forest cover and log(range size)	-0.102	0.075	-1.358	0.174
Interaction between T_{max} distance, forest cover and log(range size)	-0.022	0.008	-2.765	**0.006**
N_{Site}	7326			
N_{Study}	111			
$N_{Species}$	2255			
Observations	466939			
Marginal R^2 / Conditional R^2	0.03 / 0.72			

Appendix 2: Model coefficients

Table S8. Binomial GLMM results for model testing how T_{max} distance, forest cover, species' primary habitat preference (forest or non-forest) and their interactions affect probability of incidence.

**Incidence ~ T_{max} distance * forest cover * primary habitat preference + (1|Study / Site) + (T_{max} distance * forest cover | Species)**				
Predictors	*Log-Odds*	*std. Error*	*z*	*p*
T_{max} distance [reference level: primary habitat preference = forest]	0.092	0.017	5.285	**<0.001**
Forest cover proportion [reference level: primary habitat preference = forest]	3.551	0.209	16.974	**<0.001**
Interaction between T_{max} distance and forest cover [reference level: primary habitat preference = forest]	-0.171	0.019	-8.829	**<0.001**
Interaction between T_{max} distance and primary habitat preference [primary habitat preference = non-forest]	-0.078	0.025	-3.152	**0.002**
Interaction between forest cover and primary habitat preference [primary habitat preference = non-forest]	-2.519	0.338	-7.457	**<0.001**
Interaction between T_{max} distance , forest cover and primary habitat preference [primary habitat preference = non-forest]	0.041	0.029	1.384	0.166
N_{Site}	7326			
N_{Study}	111			
$N_{Species}$	2255			
Observations	466939			
Marginal R^2 / Conditional R^2	0.03 / 0.72			

Appendix 2: Model coefficients

Table S9. Binomial GLMM results for model testing how T_{max} distance, forest cover, species' nest type and their interactions affect probability of incidence.

**Incidence ~ T_{max} distance * forest cover * nest type + (1|Study / Site) + (T_{max} distance * forest cover | Species)**				
Predictors	*Log-Odds*	*std. Error*	*z*	*p*
T_{max} distance [reference level = closed nest]	0.055	0.018	3.026	**0.002**
Forest cover proportion [reference level = closed nest]	2.739	0.242	11.295	**<0.001**
Interaction between T_{max} distance and forest cover [reference level = closed nest]	-0.162	0.021	-7.742	**<0.001**
Interaction between T_{max} distance and nest type[open]	0.053	0.025	2.153	**0.031**
Interaction between forest cover and nest type[open]	0.844	0.316	2.667	**0.008**
Interaction between T_{max} distance, forest cover and nest type[open]	-0.083	0.029	-2.876	**0.004**
N_{Site}	7326			
N_{Study}	111			
$N_{Species}$	2068			
Observations	443110			
Marginal R^2 / Conditional R^2	0.02 / 0.72			

Table S10. Binomial GLMM results for model testing whether the effects of T_{max} distance and forest cover on probability of incidence vary depending on whether the species is migratory*. The "migratory" category includes both partially migratory and fully migratory species.*

**Incidence ~ T_{max} distance * forest cover * migration status + (1|Study / Site) + (T_{max} distance * forest cover | Species)**				
Predictors	*Log-Odds*	*std. Error*	*z*	*p*
T_{max} distance [reference level = migratory]	0.072	0.027	2.705	**0.007**
Forest cover proportion [reference level = migratory]	2.870	0.407	7.056	**<0.001**
Interaction between T_{max} distance and forest cover [reference level = migratory]	-0.197	0.031	-6.381	**<0.001**
Interaction between T_{max} distance and migration [non-migratory]	0.024	0.029	0.812	0.417
Interaction between forest cover and migration [non-migratory]	0.313	0.426	0.735	0.462
Interaction between T_{max} distance, forest cover and migration [non-migratory]	0.002	0.034	0.060	0.952
N_{Site}	7326			
N_{Study}	111			
$N_{Species}$	2256			
Observations	466968			
Marginal R^2 / Conditional R^2	0.02 / 0.72			

Table S11. Binomial GLMM results for model testing how T_{max} distance, forest cover, species' body mass in grams (log-transformed) and their interactions affect probability of incidence.

**Incidence ~ T_{max} distance * forest cover * log(mass) + (1|Study / Site) + (T_{max} distance * forest cover | Species)**				
Predictors	*Log-Odds*	*std. Error*	*z*	*p*
T_{max} distance	0.108	0.034	3.159	**0.002**
Forest cover proportion	2.535	0.446	5.683	**<0.001**
Interaction between T_{max} distance and forest cover	-0.142	0.040	-3.568	**<0.001**
Interaction between T_{max} distance and log(mass)	-0.004	0.009	-0.433	0.665
Interaction between forest cover and log(mass)	0.179	0.114	1.577	0.115
Interaction among T_{max} distance, forest cover and log(mass)	-0.016	0.010	-1.621	0.105
N_{Site}	7326			
N_{Study}	111			
$N_{Species}$	2256			
Observations	466968			
Marginal R^2 / Conditional R^2	0.03 / 0.72			

Appendix 2: Model coefficients

Table S12. Binomial GLMM results for model testing how T_{max} distance, forest cover, species' hand-wing index (HWI) and their interactions affect probability of incidence.

**Incidence ~ T_{max} distance * forest cover * HWI + (1|Study / Site) + (T_{max} distance * forest cover | Species)**				
Predictors	*Log-Odds*	*std. Error*	*z*	*p*
T_{max} distance	0.135	0.028	4.784	**<0.001**
Forest cover proportion	3.324	0.344	9.657	**<0.001**
Interaction between T_{max} distance and forest cover	-0.210	0.033	-6.425	**<0.001**
Interaction between T_{max} distance and HWI	-0.001	0.001	-1.516	0.129
Interaction between forest cover and HWI	-0.006	0.013	-0.455	0.649
Interaction among T_{max} distance, forest cover and HWI	0.000	0.001	0.275	0.783
N_{Site}	7326			
N_{Study}	111			
$N_{Species}$	2256			
Observations	466968			
Marginal R^2 / Conditional R^2	0.03 / 0.72			

Table S13. Binomial GLMM results for model testing how T_{max} distance, forest cover, species' primary foraging height and their interactions affect probability of incidence.

**Incidence ~ T_{max} distance * forest cover * height + (1|Study / Site) + (T_{max} distance * forest cover | Species)**				
Predictors	*Log-Odds*	*std. Error*	*z*	*p*
T_{max} distance [reference level = canopy]	0.062	0.037	1.687	0.092
Forest cover proportion [reference level = canopy]	3.003	0.449	6.684	**<0.001**
Interaction between T_{max} distance and forest cover proportion [reference level = canopy]	-0.171	0.042	-4.088	**<0.001**
Interaction between T_{max} distance and height [ground]	0.046	0.041	1.125	0.261
Interaction between T_{max} distance and height [mid-height]	0.022	0.043	0.520	0.603
Interaction between T_{max} distance and height [understory]	0.019	0.043	0.450	0.653
Interaction between forest cover proportion and height [ground]	0.227	0.514	0.441	0.659
Interaction between forest cover proportion and height [mid-height]	0.238	0.517	0.460	0.645
Interaction between forest cover proportion and height [understory]	-0.195	0.529	-0.368	0.713
Interaction among T_{max} distance, forest cover proportion and height [ground]	-0.073	0.047	-1.540	0.124
Interaction among T_{max} distance, forest cover proportion and height [mid-height]	0.005	0.049	0.096	0.924
Interaction among T_{max} distance, forest cover proportion and height [understory]	0.022	0.049	0.452	0.651
N_{Site}	7326			
N_{Study}	111			
$N_{Species}$	2170			
Observations	457004			
Marginal R^2 / Conditional R^2	0.02 / 0.72			

Appendix 2: Model coefficients

Table S14. Binomial GLMM results for model testing how T_{max} distance, forest cover, species' primary diet and their interactions affect probability of incidence. *Species were assigned to one of five diet categories* [1]*: (1) species that mainly eat plants and seeds (*PlantSeed*). (2) species that mainly eat fruits and nectar (*FruiNect*). (3) species that mainly eat invertebrates (*Invertebrate*). (4) species that mainly eat vertebrates, fish and carrion (*VertFishScav*). (5) species that eat more than one of the above categories (*Omnivore*).*

**Incidence ~ T_{max} distance * forest cover * diet + (1|Study / Site) + (T_{max} distance * forest cover | Species)**				
Predictors	*Log-Odds*	*std. Error*	*z*	*p*
T_{max} distance [reference level = FruiNect]	0.106	0.032	3.276	**0.001**
Forest cover proportion [reference level = FruiNect]	3.580	0.379	9.439	**<0.001**
Interaction between T_{max} distance and Forest cover proportion [reference level = FruiNect]	-0.197	0.037	-5.323	**<0.001**
Interaction between T_{max} distance and diet [Invertebrate]	0.001	0.034	0.025	0.980
Interaction between T_{max} distance and diet [Omnivore]	-0.064	0.042	-1.518	0.129
Interaction between T_{max} distance and diet [PlantSeed]	0.015	0.051	0.287	0.774
Interaction between T_{max} distance and diet [VertFishScav]	-0.089	0.061	-1.462	0.144
Interaction between forest cover proportion and diet [Invertebrate]	-0.310	0.412	-0.751	0.453
Interaction between forest cover proportion and diet [Omnivore]	-1.317	0.520	-2.530	**0.011**
Interaction between forest cover proportion and diet [PlantSeed]	-1.165	0.685	-1.699	0.089
Interaction between forest cover proportion and diet [VertFishScav]	-0.981	0.860	-1.141	0.254
Interaction among T_{max} distance, forest cover proportion and diet [Invertebrate]	-0.002	0.040	-0.051	0.959
Interaction among T_{max} distance, forest cover proportion and diet [Omnivore]	0.054	0.049	1.101	0.271
Interaction among T_{max} distance, forest cover proportion and diet [PlantSeed]	-0.040	0.061	-0.659	0.510

Interaction among T_{max} distance, forest cover proportion and diet [VertFishScav]	0.057	0.073	0.782	0.434
N $_{Site}$	7326			
N $_{Study}$	111			
N $_{Species}$	2251			
Observations	467277			
Marginal R^2 / Conditional R^2	0.03 / 0.72			

Table S15. Linear mixed effect model results for model testing how forest cover affects the maximum microclimate temperature in each biogeographic realm.

**Maximum site temperature ~ forest cover * realm + (forest cover | study)**				
Predictors	*Estimates*	*std. Error*	*z*	*p*
(Intercept) [reference level = Afrotropical]	27.726	1.093	25.369	**<0.001**
Forest cover proportion [reference level = Afrotropical]	-4.478	1.041	-4.301	**<0.001**
(Intercept) Realm [Indo-Malayan]	2.529	1.720	1.470	0.141
(Intercept) Realm [Nearctic]	-4.839	2.124	-2.278	**0.023**
(Intercept) Realm [Neotropical]	-0.522	1.299	-0.401	0.688
(Intercept) Realm [Australasian]	-1.924	2.369	-0.812	0.417
(Intercept) Realm [Palearctic]	-2.342	1.369	-1.711	0.087
Interaction between forest cover proportion and realm [Indo-Malayan]	-0.002	1.716	-0.001	0.999
Interaction between forest cover proportion and realm [Nearctic]	0.604	1.998	0.302	0.763
Interaction between forest cover proportion and realm [Neotropical]	0.553	1.259	0.440	0.660
Interaction between forest cover proportion and realm [Australasian]	-1.108	2.294	-0.483	0.629
Interaction between forest cover proportion and realm [Palearctic]	-3.052	1.334	-2.288	**0.022**
N $_{Study}$	109			
Observations	7286			
Marginal R^2 / Conditional R^2	0.35 / 0.91			

Appendix 2: Model coefficients

Table S16. Linear mixed effect model results for model testing how forest cover affects the maximum microclimate temperature in each biome.

**Maximum site temperature ~ forest cover * biome + (forest cover | Study)**				
Predictors	*Estimates*	*std. Error*	*z*	*p*
(Intercept) [reference level = Mediterranean Forests, Woodlands & Scrub]	28.505	1.056	27.001	**<0.001**
Forest cover proportion [reference level = Mediterranean Forests, Woodlands & Scrub]	-9.194	1.184	-7.765	**<0.001**
(Intercept) Biome [Temperate Broadleaf & Mixed Forests]	-4.132	1.380	-2.994	**0.003**
(Intercept) Biome [Temperate Conifer Forests]	-8.820	2.264	-3.896	**<0.001**
(Intercept) Biome [Tropical & Subtropical Coniferous Forests]	-1.471	2.798	-0.526	0.599
(Intercept) Biome [Tropical & Subtropical Dry Broadleaf Forests]	4.139	2.102	1.969	**0.049**
(Intercept) Biome [Tropical &Subtropical Moist Broadleaf Forests]	-1.067	1.165	-0.916	0.360
Interaction between forest cover proportion and biome [Temperate Broadleaf & Mixed Forests]	3.417	1.519	2.249	**0.024**
Interaction between forest cover proportion and biome [Temperate Conifer Forests]	5.218	2.342	2.228	**0.026**
Interaction between forest cover proportion and biome [Tropical & Subtropical Coniferous Forests]	7.345	3.381	2.172	**0.030**
Interaction between forest cover proportion and biome [Tropical & Subtropical Dry Broadleaf Forests]	-0.156	2.442	-0.064	0.949
Interaction between forest cover proportion and biome [Tropical & Subtropical Moist Broadleaf Forests]	5.124	1.295	3.958	**<0.001**
N_{Study}	108			
Observations	7238			
Marginal R^2 / Conditional R^2	0.45 / 0.91			

Appendix 3: Testing for and accounting for phylogenetic non-independence among species

We assessed the robustness of our results to phylogenetic non-independence among species in two ways. (1) Using phylogenetic tree data from OpenTree,[2] we quantified phylogenetic signal in species' level random effect coefficients and found that phylogenetic signal was very low. (2) As a sensitivity test, we then incorporated phylogenetic non-independence in our main GLMM and found that it did not affect our results.

We used random slopes in our statistical models to account for variation in the fixed effects across species. To test whether these slopes were phylogenetically correlated, we used phylogenetic generalised least squares (PGLS) to estimate λ, a measure of phylogenetic signal. A value of 0 indicates no phylogenetic signal and a value of 1 indicates strong phylogenetic signal. Using the model reported in Table S2 and Figure 2A, where T_{max} *distance* and forest cover proportion were interacting predictors of species incidence, we extracted the random effect slopes and intercepts for each species for each model coefficient. We then used these slopes and intercepts as response variables in separate PGLS models We used null PGLS models (coefficient ~ 1) and estimated λ by maximum likelihood using the caper R package [3]. There was very low phylogenetic signal in the species' coefficients and intercepts.

***Table S17. Phylogenetic signal, measured by λ in PGLS models, of species-level random effect coefficients*.**

**Coefficient**	**λ (95% CI)**
Intercept	0.099 (0.039, 0.198)
T_{max} distance	0.029 (0.002, 0.081)
Forest cover	0.084 (0.034, 0.166)
Interaction between T_{max} distance and forest cover	0.029 (0.005, 0.081)

Appendix 3: Testing for and accounting for phylogenetic non-independence among species

To test whether incorporating phylogenetic non-independence affected our results, we fitted a phylogenetic generalised linear mixed model using the *glmmTMB* R package [4,5]. We retained the same fixed-effects structure as the main analysis, along with the same random effects. In addition, we included a phylogenetic random effect specified using a correlation matrix derived from the species-level phylogeny obtained from OpenTree [2]. Accounting for phylogenetic relatedness did not affect the direction or magnitude of the fixed effects, indicating that our results are robust to phylogenetic non-independence.

Table S18. Binomial GLMM results for model testing how T_{max} distance, forest cover and their interaction affect probability of incidence when accounting for phylogenetic non-independence.

Incidence ~ T_{max} distance * forest cover + (1 | Study/Site) + (1 + forest cover * T_{max} distance | Species) + propto(0 + Species | g, phylo.mat)				
Predictors	*Log-Odds*	*std. Error*	*z*	*p*
T_{max} distance	0.097	0.015	6.640	**<0.001**
Forest cover	3.146	0.188	16.754	**<0.001**
Interaction between T_{max} distance and forest cover	-0.198	0.016	-12.044	**<0.001**
N_{Site}	7326			
N_{Study}	111			
$N_{Species}$	2243			
Observations	465718			

Appendix 4: Accounting for latitude

T_{max} *distance* is correlated with absolute latitude (correlation coefficient = 0.47), and latitude has been shown to mediate responses to forest cover, with species at lower latitudes showing heightened sensitivity to forest cover [6–8]. Therefore, we tested whether accounting for the potentially confounding effect of latitude changes the effect of T_{max} *distance* on responses to forest cover. We found that the effect of T_{max} *distance* on responses to forest cover was robust to accounting for latitude.

We found that latitude mediated responses to forest cover (Table S18; Figure S1), consistent with previous studies [6–8], but latitude did not have a significant effect when it was included in a model with T_{max} *distance* (Table S19; Figure S2). The effect of T_{max} *distance* on response to forest cover, as reported in the main text (e.g. Table S2; Figure 2A) remained the same even when accounting for the effect of latitude on response to forest cover (Table S19; Figure S2).

Appendix 4: Accounting for latitude

Table S19. Binomial GLMM results for model testing how absolute latitude, forest cover and their interaction affect probability of incidence

**Incidence ~ latitude * forest cover + (1|Study / Site) + (latitude * forest cover | Species)**				
Predictors	*Log-Odds*	*std. Error*	*z*	*p*
Latitude	0.037	0.011	3.366	**0.001**
Forest cover proportion	1.833	0.163	11.255	**<0.001**
Latitude * forest cover	-0.034	0.007	-4.921	**<0.001**
N_{Site}	7326			
N_{Study}	111			
$N_{Species}$	2262			
Observations	467333			
Marginal R^2 / Conditional R^2	0.02 / 0.76			
AIC	294460			

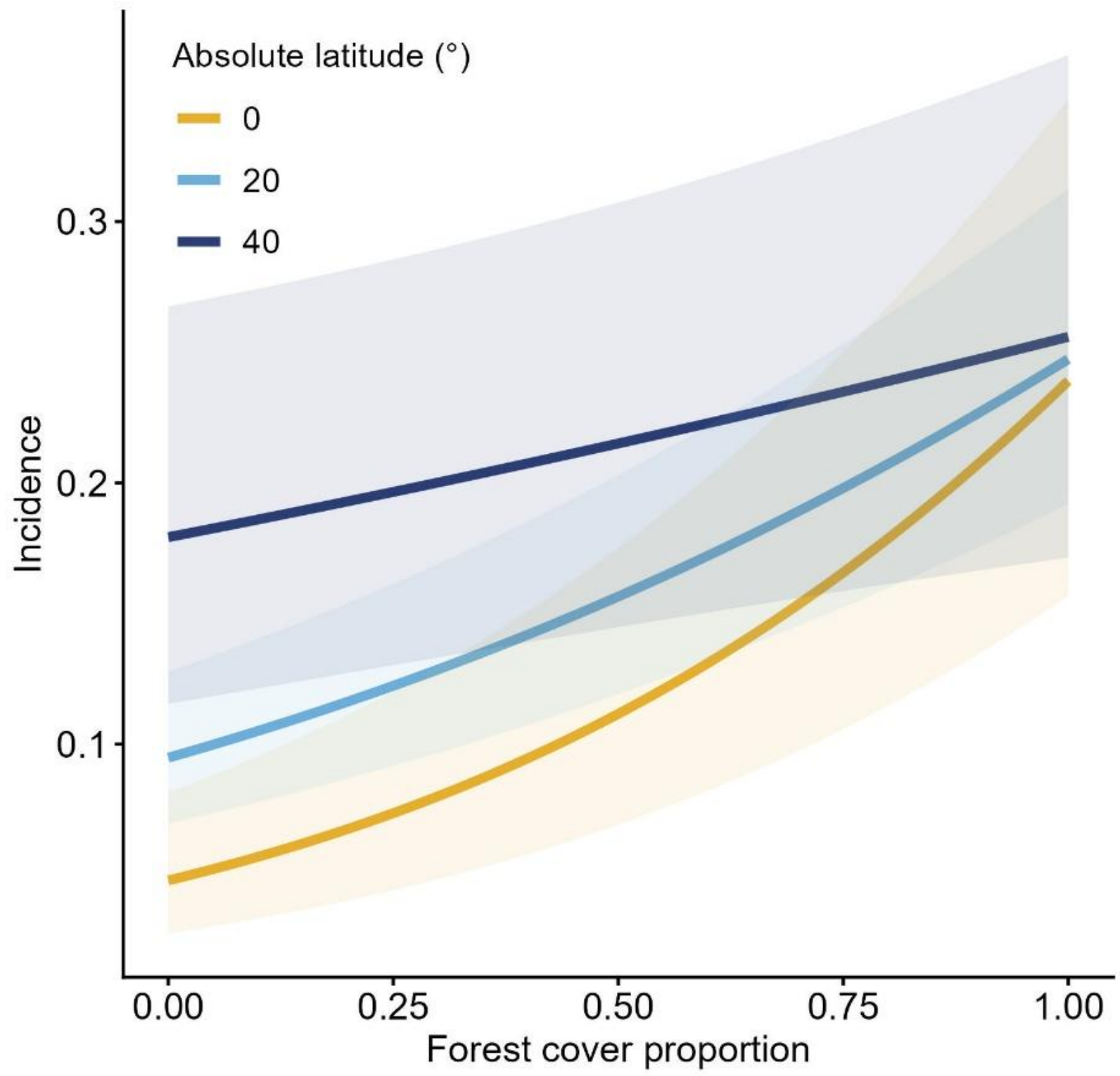

Figure S1. Latitude mediates variation in how forest cover affects incidence. *Lines show modelled relationships for populations at different latitudes: 0°, 20°, and 40°. The shading shows the 95% confidence intervals.*

Table S20. Binomial GLMM results for model testing how T_{max} distance, forest cover and their interaction affect probability of incidence when accounting for the effect of latitude on responses to forest cover.

Incidence ~ T_{max} distance * forest cover + Latitude * forest cover + (1|Study / Site) + (T_{max} distance * forest cover + Latitude * forest cover | Species)

Predictors	*Log-Odds*	*std. Error*	*z*	*p*
T_{max} distance	0.091	0.017	5.426	**<0.001**
Forest cover proportion	3.342	0.203	16.441	**<0.001**
Latitude	0.017	0.011	1.470	0.142
Interaction between T_{max} distance and forest cover	-0.219	0.019	-11.722	**<0.001**
Interaction between latitude and forest cover	0.004	0.007	0.472	0.637
N_{Site}	7326			
N_{Study}	111			
$N_{Species}$	2262			
Observations	467333			
Marginal R^2 / Conditional R^2	0.02 / 0.80			
AIC	289212			

Appendix 4: Accounting for latitude

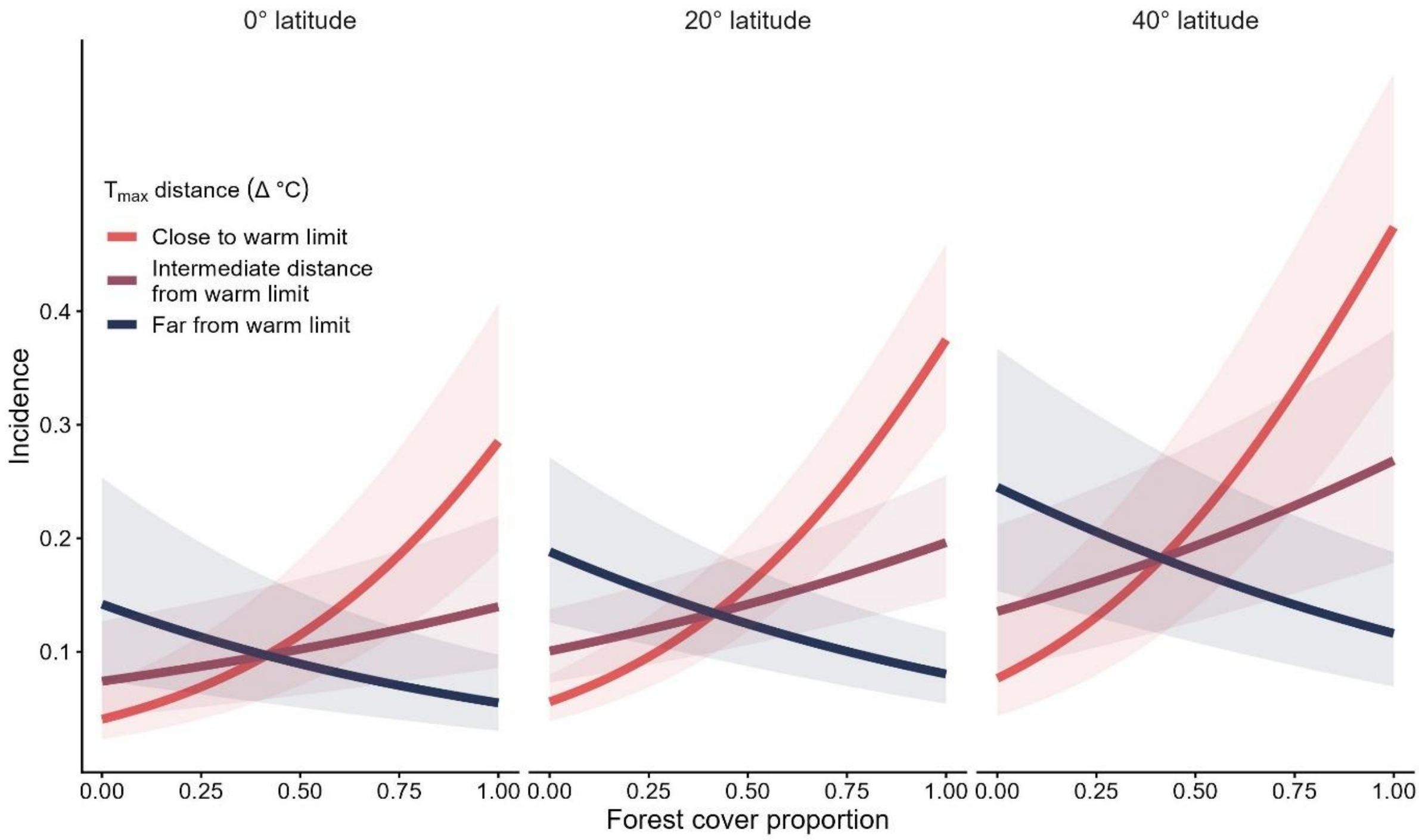

Figure S2. Across latitudes, T_{max} distance mediates variation in how forest cover affects incidence. *Panels show modelled relationships at 0°, 20°, and 40° latitude. Lines represent populations differing in T_{max} distance: 5 °C from the warm limit (pink; close to the warm limit), 12 °C (purple; intermediate), and 20 °C (blue; far from the warm limit). The shading shows the 95% confidence intervals.*

Appendix 5: Sensitivity test for including non-forest species

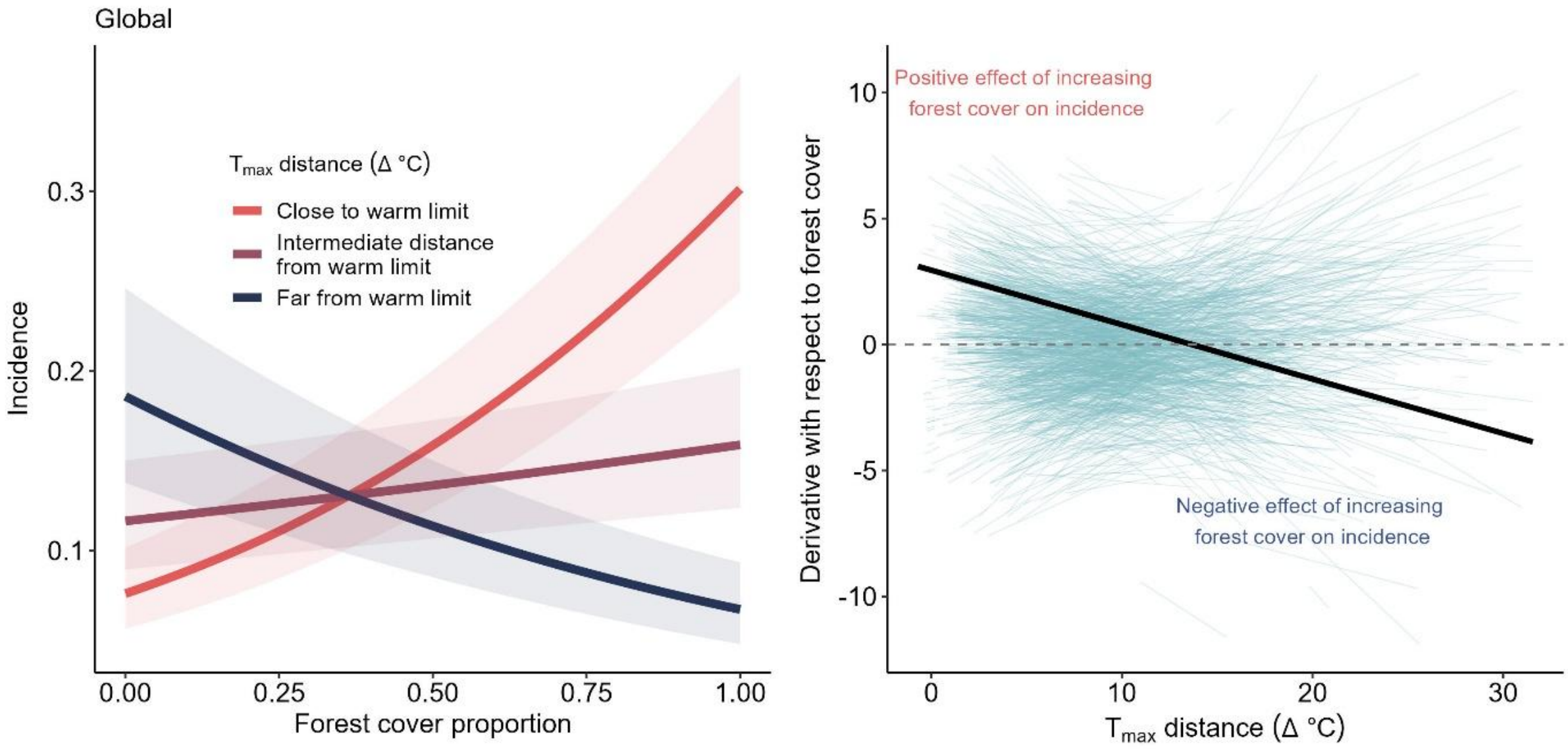

Figure S3. When including non-forest species, T_{max} distance mediates inter- and intraspecific variation in bird responses to forest cover. *The left panel shows how forest cover proportion within a 600 m radius influences bird incidence across populations at different distances from their species' realised warm tolerance limits (T_{max} distances). The lines represent populations near (pink line, 10th percentile), intermediate (purple, 50th percentile) and far (blue, 90th percentile) from these limits. The shading shows the 95% confidence intervals. The right panel illustrates the within-species variation. Each thin line represents one species and the thick black line shows the overall mean. The y-axis indicates the direction and strength of the response to forest cover, with the grey dotted line marking the point where the response to increasing forest cover changes from negative to positive. Populations closer to their species' realised warm tolerance limits generally show more positive responses to increases in forest cover.*

Appendix 5: Sensitivity test for including non-forest species

Table S21. When including non-forest species, T_{max} distance mediates inter- and intraspecific variation in bird responses to forest cover. *The table shows the binomial GLMM results for model testing how T_{max} distance, forest cover and their interaction affect probability of incidence for all bird species, including those who do not have forest listed as one of their habitat affiliations according to the IUCN classification [9]. The results are similar to those presented in the main manuscript, indicating that our findings are robust to the decision to exclude non-forest species.*

**Incidence ~ T_{max} distance * forest cover + (1|Study / Site) + (T_{max} distance * forest cover | Species)**				
Predictors	*Log-Odds*	*std. Error*	*z*	*p*
T_{max} distance	0.078	0.013	6.045	**<0.001**
Forest cover proportion	2.951	0.180	16.422	**<0.001**
Interaction between T_{max} distance and forest cover	-0.216	0.016	-13.860	**<0.001**
N_{Site}	7326			
N_{Study}	111			
$N_{Species}$	2522			
Observations	544083			
Marginal R^2 / Conditional R^2	0.02 / 0.71			

Appendix 5: Sensitivity test for including non-forest species

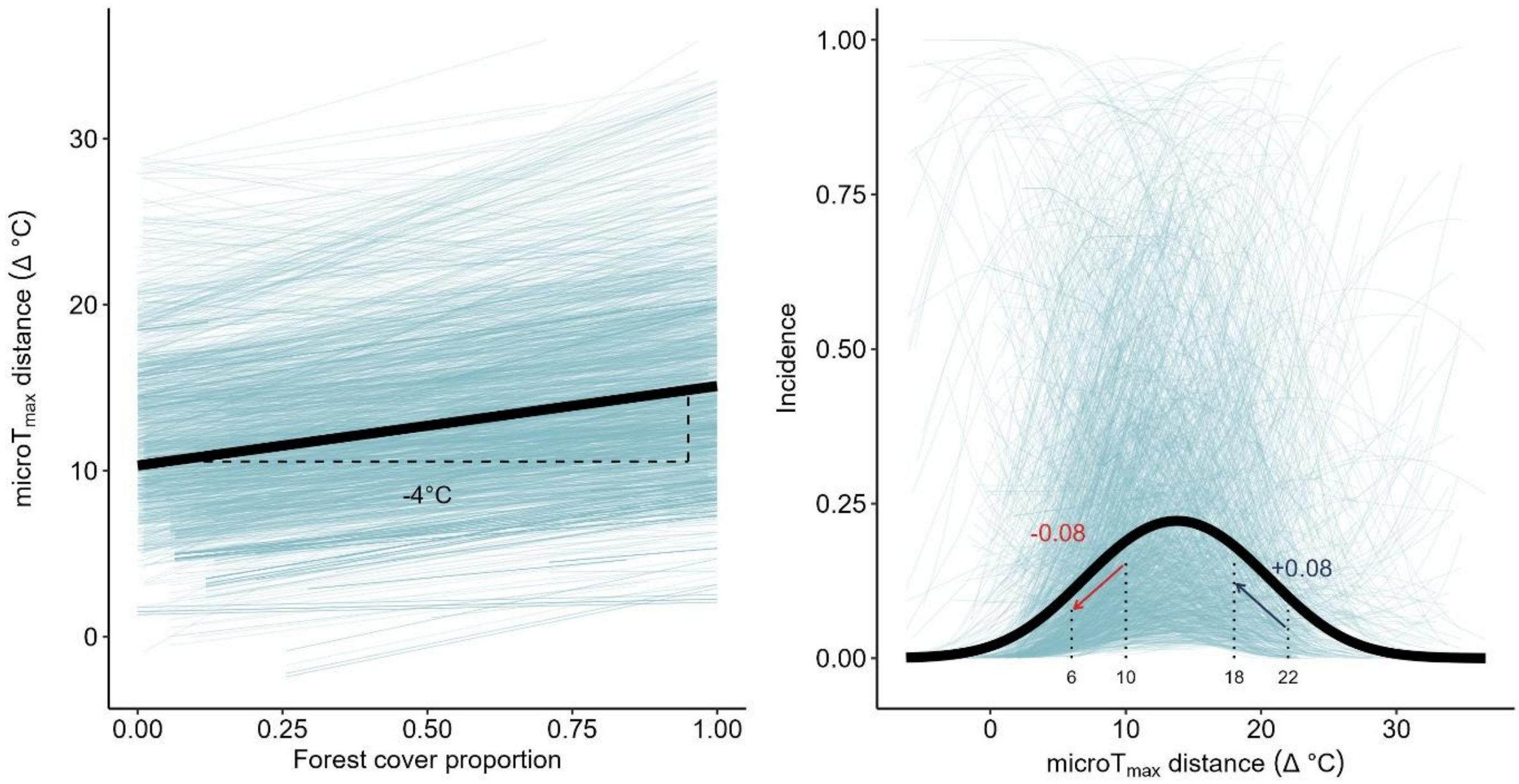

Figure S4. When including non-forest species, sites at low forest cover are closer to species' warm limits and this microclimatic shift can affect populations differently depending on the macroclimate in which they are located. *The left panel shows how lower forest cover is associated with a reduction in microT$_{max}$ distance. Lower values of microT$_{max}$ distance indicate microclimatic temperatures closer to a species' warm limit. The thick black line shows the average relationship across all species, and the thin blue lines show the relationships for each species. For a change in forest cover from 95% to 5%, there is a 4°C reduction in the average microT$_{max}$ distance. The right panel shows the effect of microT$_{max}$ distance on incidence. The thick black line shows the overall relationship, and the thin blue lines show the relationships for each species. We annotate an example of how a 4°C decrease in microT$_{max}$ distance can have contrasting effects on species' incidence depending on the starting microT$_{max}$ distance.*

Appendix 5: Sensitivity test for including non-forest species

Table S22. Linear mixed effect model results for model testing how forest cover affects microT_{max} distance, when including non-forest species.

**microT_{max} distance ~ Forest cover proportion + (Forest cover proportion | Species)**				
Predictors	*Estimates*	*std. Error*	*z*	*p*
Forest cover proportion	4.785	0.086	55.758	**<0.001**
N $_{Species}$	2522			
Observations	544083			
Marginal R^2 / Conditional R^2	0.08 / 0.85			

Table S23. Binomial GLMM results for model testing how microT_{max} distance affects probability of incidence, when including non-forest species.

**Incidence ~ microT_{max} distance^2 + (1 |Study/Site) + (microT_{max} distance^2 | Species)**				
Predictors	*Log-Odds*	*std. Error*	*z*	*p*
microT_{max} distance [1st degree]	0.388	0.024	16.001	**<0.001**
microT_{max} distance [2nd degree]	-0.014	0.001	-15.708	**<0.001**
N $_{Site}$	7326			
N $_{Study}$	111			
N $_{Species}$	2522			
Observations	544083			
Marginal R^2 / Conditional R^2	0.05 / 0.71			

Appendix 6: Sensitivity test for tree cover thresholds used to calculate forest cover proportions in the landscape surrounding each site

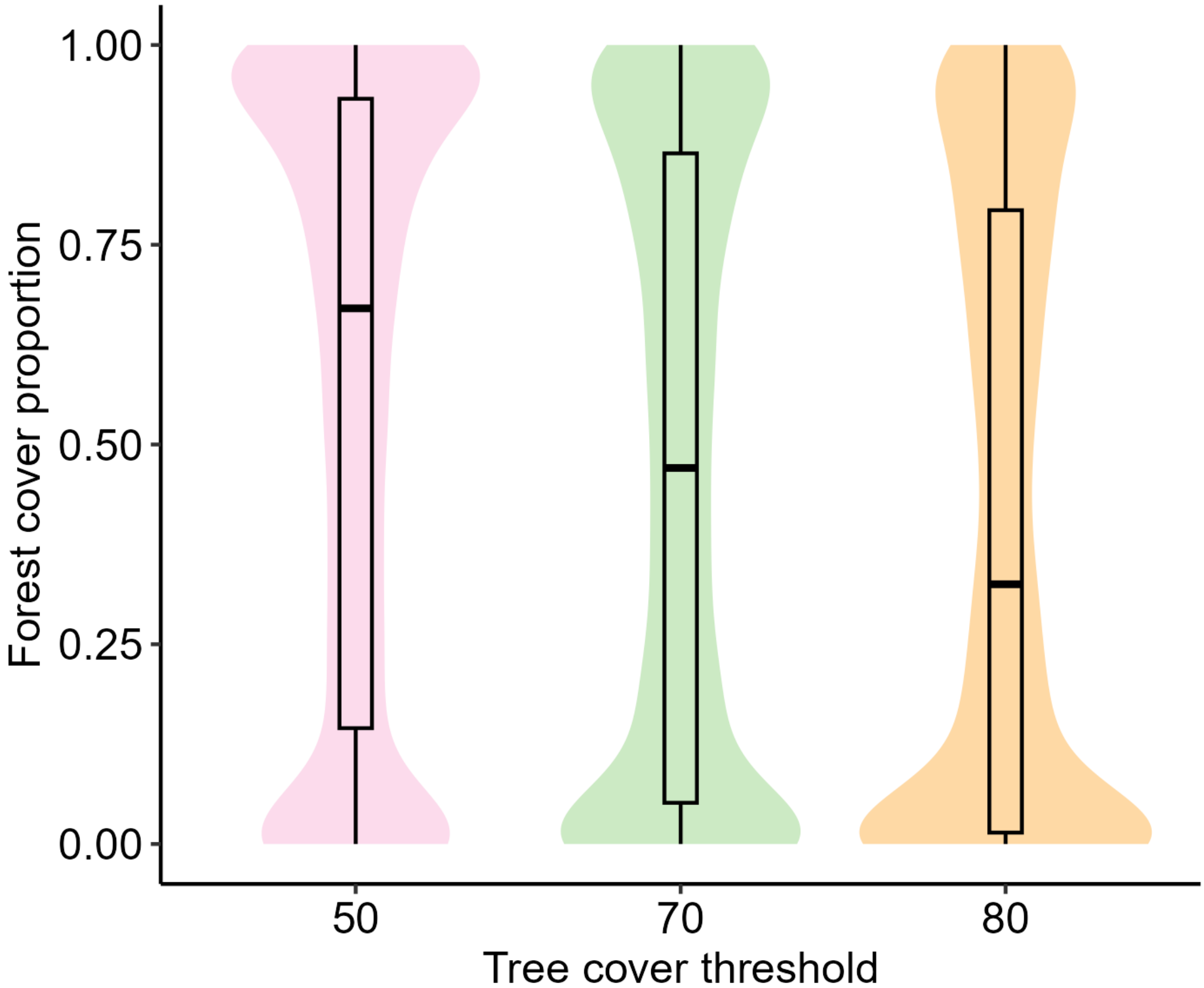

Figure S5. Distribution of forest cover proportions across all sites, calculated using various thresholds of tree cover for defining forests. *The horizontal line inside each box represents the median forest cover proportion at each tree cover threshold. The boxes represent the inter-quartile range, and the lines extending from the boxes represent the range. The violin plots around each box indicate the distribution of data.*

Appendix 6: Sensitivity test for tree cover thresholds used to calculate forest cover proportions

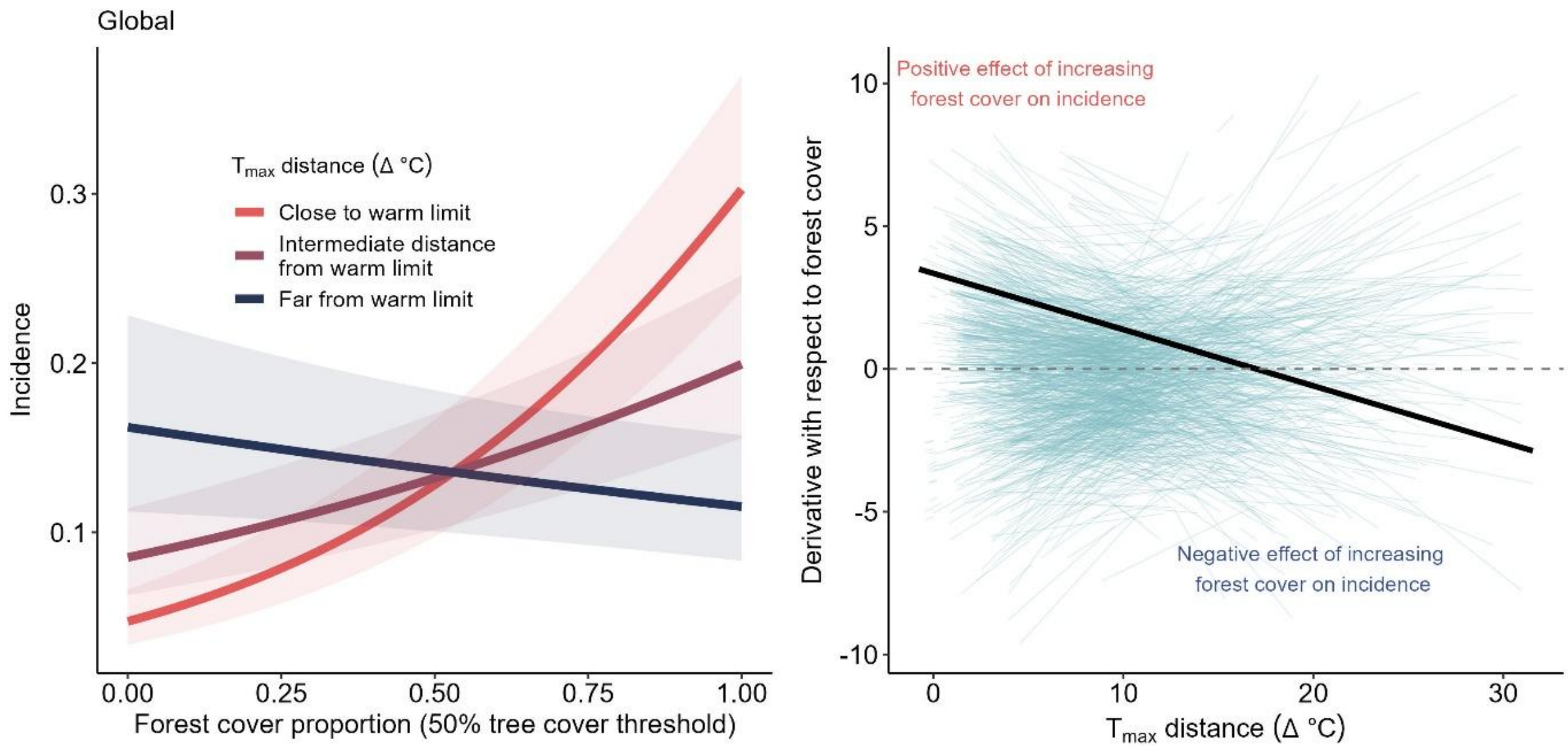

Figure S6. When using a 50% tree cover threshold to calculate forest cover, T_{max} distance mediates inter- and intraspecific variation in bird responses to forest cover. *The left panel shows how forest cover proportion within a 600 m radius influences bird incidence across populations at different distances from their species' realised warm tolerance limits (T_{max} distances). The lines represent populations near (pink line, 10th percentile), intermediate (purple, 50th percentile) and far (blue, 90th percentile) from these limits. The shading shows the 95% confidence intervals. The right panel illustrates the within-species variation. Each thin line represents one species and the thick black line shows the overall mean. The y-axis indicates the direction and strength of the response to forest cover, with the grey dotted line marking the point where the response to increasing forest cover changes from negative to positive. Populations closer to their species' realised warm tolerance limits generally show more positive responses to increases in forest cover.*

Appendix 6: Sensitivity test for tree cover thresholds used to calculate forest cover proportions

Table S24. Model coefficients for model testing how T_{max} distance, forest cover proportion and their interaction affect probability of species incidence. *Forest cover proportion was calculated using a* ***50% threshold*** *to convert the tree cover map into a binary map of forest and non-forest (rather than the 70% threshold used in the main manuscript). Varying the threshold did not affect our results.*

**Tree cover threshold used for calculating forest cover proportion = 50%**				
Predictors	Log-Odds	std. Error	z	p
T_{max} distance	0.105	0.017	6.175	**<0.001**
Forest cover proportion	3.436	0.211	16.304	**<0.001**
Interaction between T_{max} distance and forest cover	-0.201	0.018	-11.014	**<0.001**
N_{Site}	7282			
N_{Study}	109			
$N_{Species}$	2255			
Observations	463429			
Marginal R^2 / Conditional R^2	0.02 / 0.73			

Appendix 6: Sensitivity test for tree cover thresholds used to calculate forest cover proportions

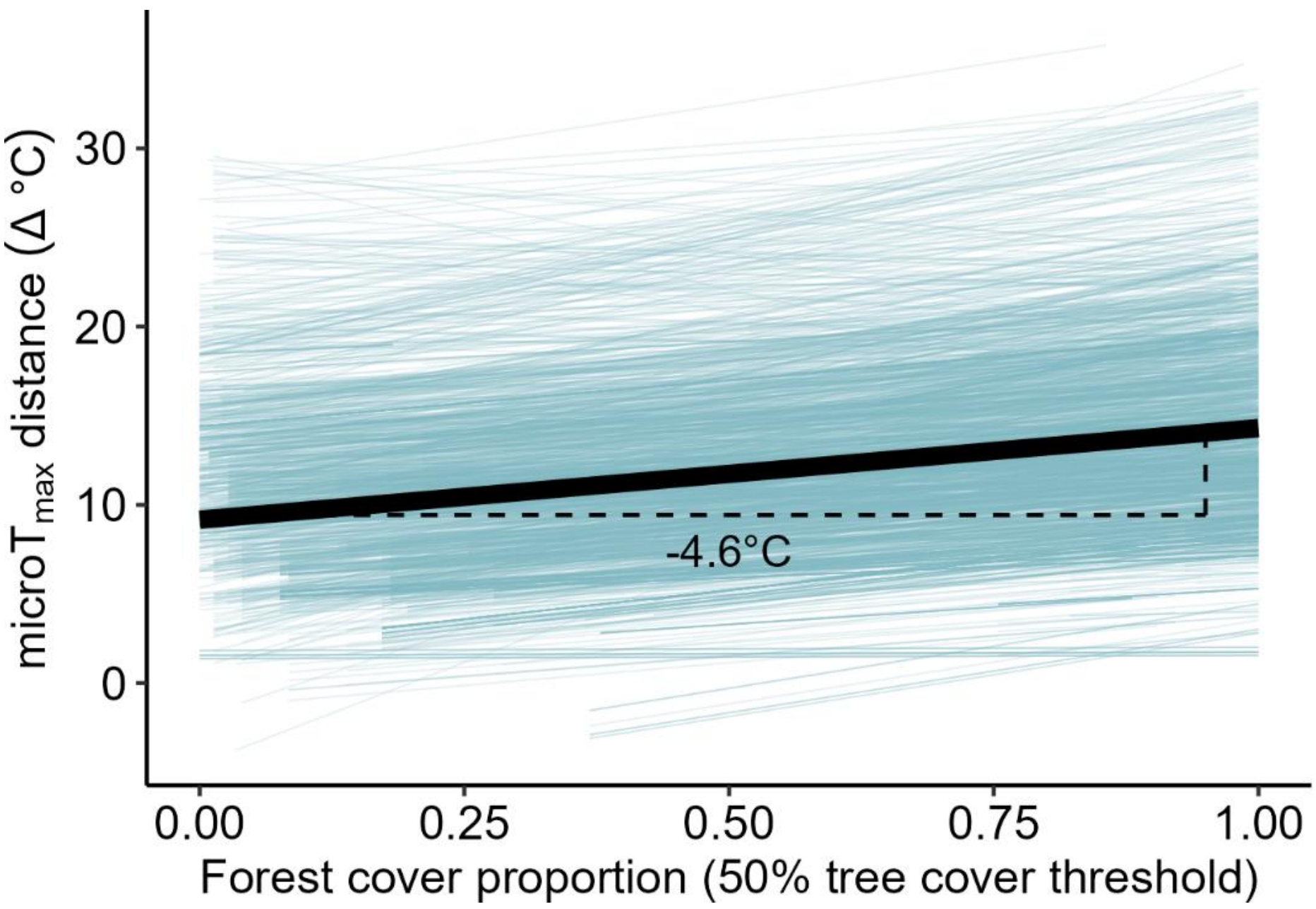

Figure S7. When using a 50% tree cover threshold for calculating forest cover proportion, lower forest cover is associated with a reduction in microT$_{max}$ distance. *Lower values of microT$_{max}$ distance indicate microclimatic temperatures closer to a species' warm limit. The thick black line shows the average relationship across all species, and the thin blue lines show the relationships for each species. For a change in forest cover from 95% to 5%, there is a 4.6°C reduction in the average microT$_{max}$ distance.*

Table S25. Linear mixed effect model results for model testing how forest cover affects microT$_{max}$ distance. *Forest cover proportion was calculated using a* ***50% threshold*** *to convert the tree cover map into a binary map of forest and non-forest (rather than the 70% threshold used in the main analyses).*

**Tree cover threshold used for calculating forest cover proportion = 50%**				
Predictors	*Estimates*	*std. Error*	*z*	*p*
Forest cover proportion	5.141	0.096	53.492	**<0.001**
N Species	2256			
Observations	469309			
Marginal R^2 / Conditional R^2	0.08 / 0.84			

Appendix 6: Sensitivity test for tree cover thresholds used to calculate forest cover proportions

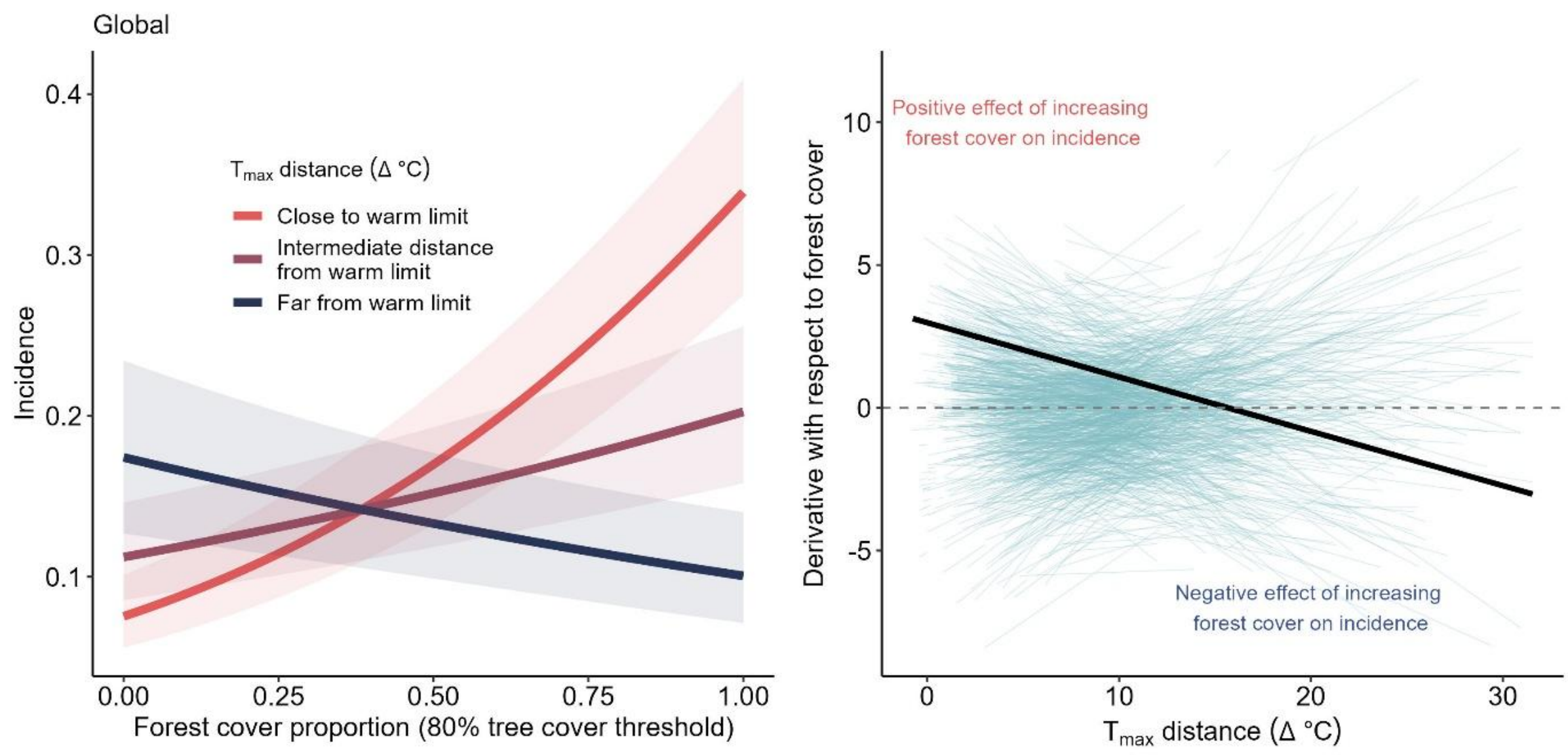

Figure S8. When using an 80% tree cover threshold to calculate forest cover, T_{max} distance mediates inter- and intraspecific variation in bird responses to forest cover. *The left panel shows how forest cover proportion within a 600 m radius influences bird incidence across populations at different distances from their species' realised warm tolerance limits (T_{max} distances). The lines represent populations near (pink line, 10th percentile), intermediate (purple, 50th percentile) and far (blue, 90th percentile) from these limits. The shading shows the 95% confidence intervals. The right panel illustrates the within-species variation. Each thin line represents one species and the thick black line shows the overall mean. The y-axis indicates the direction and strength of the response to forest cover, with the grey dotted line marking the point where the response to increasing forest cover changes from negative to positive. Populations closer to their species' realised warm tolerance limits generally show more positive responses to increases in forest cover.*

Appendix 6: Sensitivity test for tree cover thresholds used to calculate forest cover proportions

Table S26. Model coefficients for model testing how T_{max} distance, forest cover proportion and their interaction affect probability of species incidence. *Forest cover proportion was calculated using an **80% threshold** to convert the tree cover map into a binary map of forest and non-forest (rather than the 70% threshold used in the main analyses). Varying the threshold did not affect our results.*

**Tree cover threshold used for calculating forest cover proportion = 80%**				
Predictors	*Log-Odds*	*std. Error*	*z*	*p*
T_{max} distance	0.073	0.013	5.560	**<0.001**
Forest cover proportion	2.981	0.180	16.603	**<0.001**
Interaction between T_{max} distance and forest cover	-0.190	0.016	-11.932	**<0.001**
N_{Site}	7131			
N_{Study}	110			
$N_{Species}$	2279			
Observations	469251			
Marginal R^2 / Conditional R^2	0.02 / 0.71			

Appendix 6: Sensitivity test for tree cover thresholds used to calculate forest cover proportions

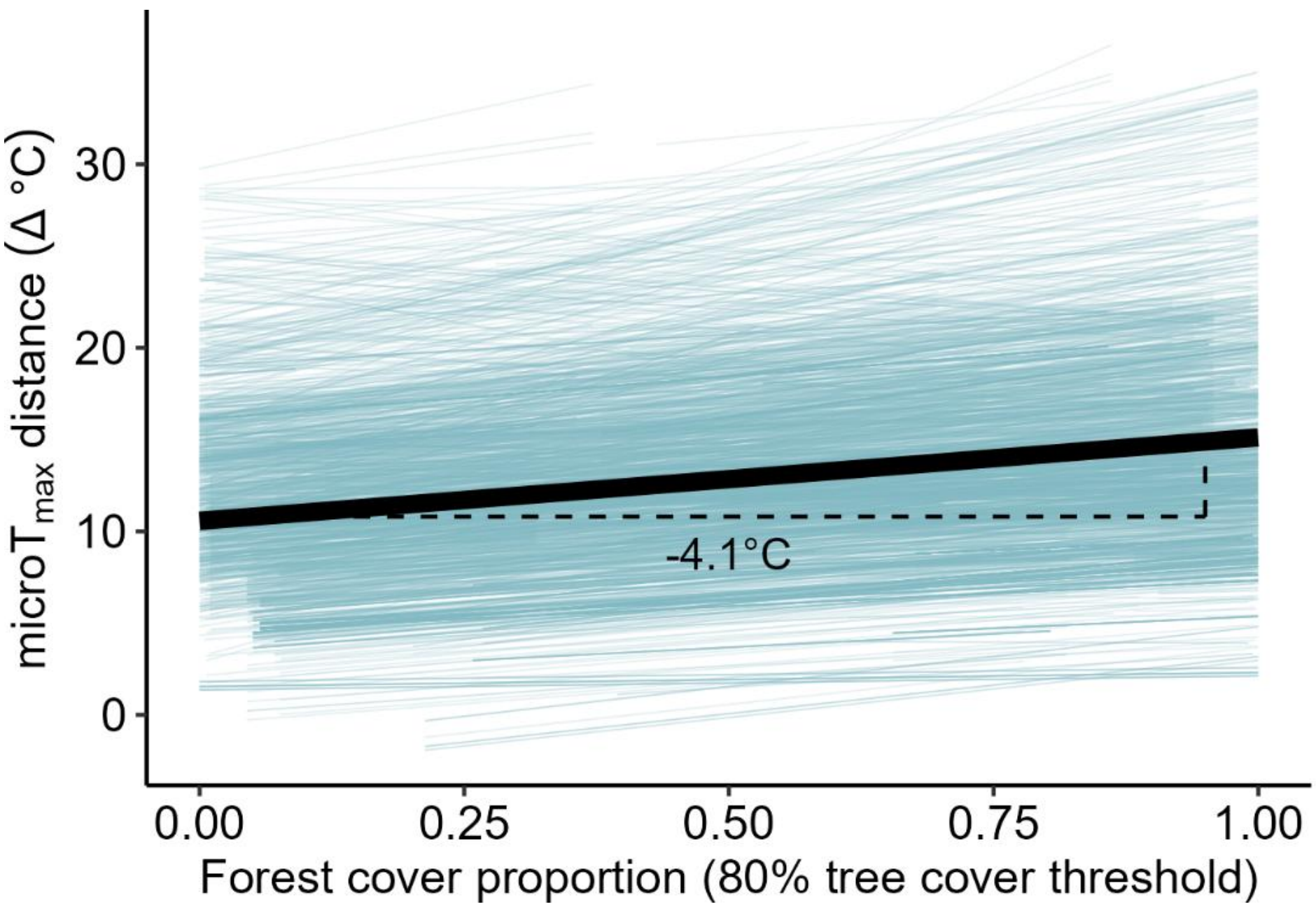

Figure S9. When using an 80% tree cover threshold for calculating forest cover proportion, lower forest cover is associated with a reduction in microT$_{max}$ distance. *Lower values of microT$_{max}$ distance indicate microclimatic temperatures closer to a species' warm limit. The thick black line shows the average relationship across all species, and the thin blue lines show the relationships for each species. For a change in forest cover from 95% to 5%, there is a 4.1°C reduction in the average microT$_{max}$ distance.*

Table S27. Linear mixed effect model results for model testing how forest cover affects microT$_{max}$ distance. *Forest cover proportion was calculated using an* ***80% threshold*** *to convert the tree cover map into a binary map of forest and non-forest (rather than the 70% threshold used in the main analyses).*

**Tree cover threshold used for calculating forest cover proportion = 80%**				
Predictors	*Estimates*	*std. Error*	*z*	*p*
Forest cover proportion	4.548	0.092	49.236	**<0.001**
N $_{Species}$	2279			
Observations	469251			
Marginal R^2 / Conditional R^2	0.08 / 0.83			

Appendix 7: Sensitivity test for buffer sizes used to calculate forest cover proportions in the landscape surrounding each site

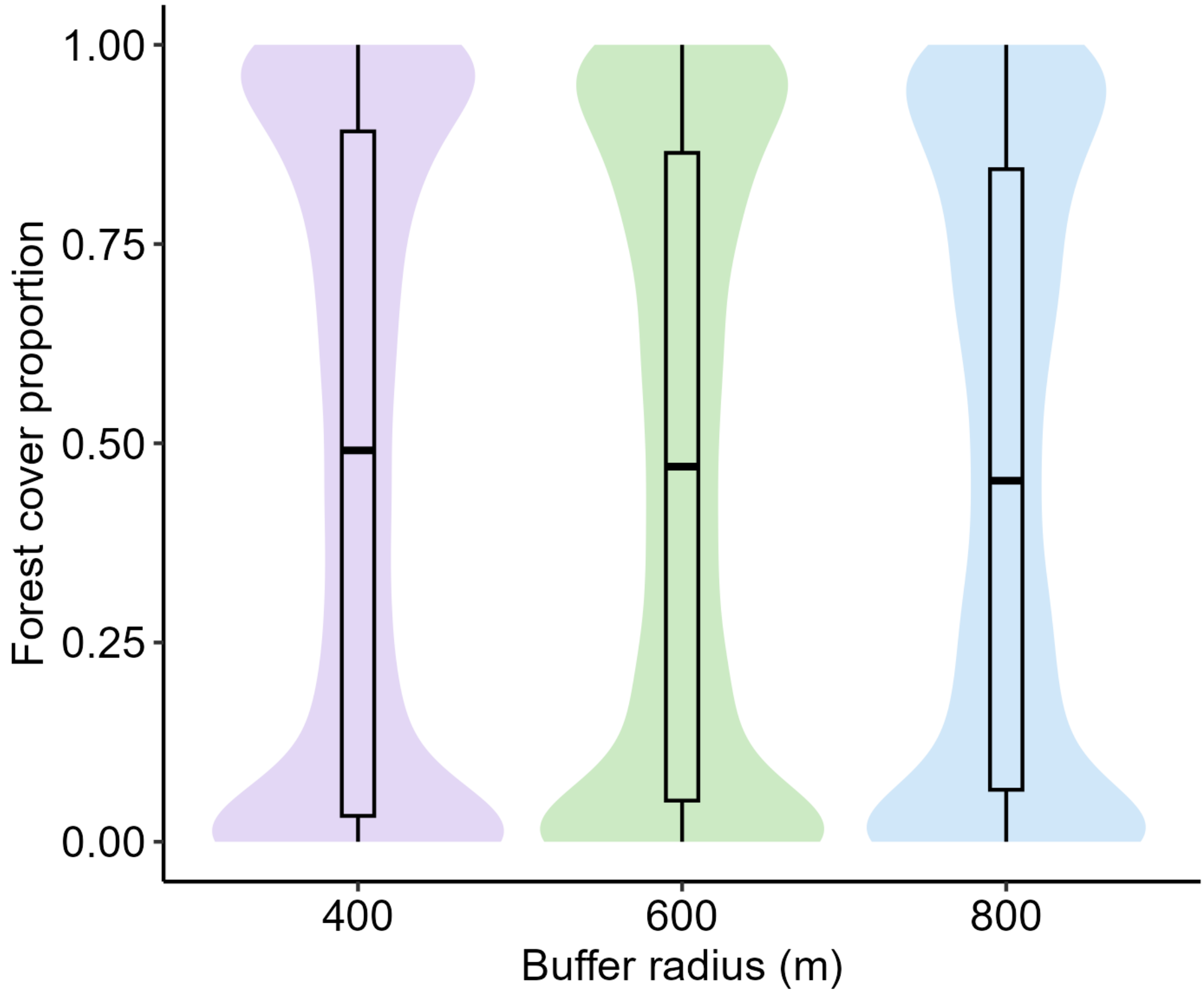

Figure S10. Distribution of forest cover proportions across all sites, calculated using various buffer sizes around each site. *The horizontal line inside each box represents the median forest cover proportion. The boxes represent the inter-quartile range, and the lines extending from the boxes represent the range. The violin plots around each box indicate the distribution of data.*

Appendix 7: Sensitivity test for buffer sizes used to calculate forest cover proportions

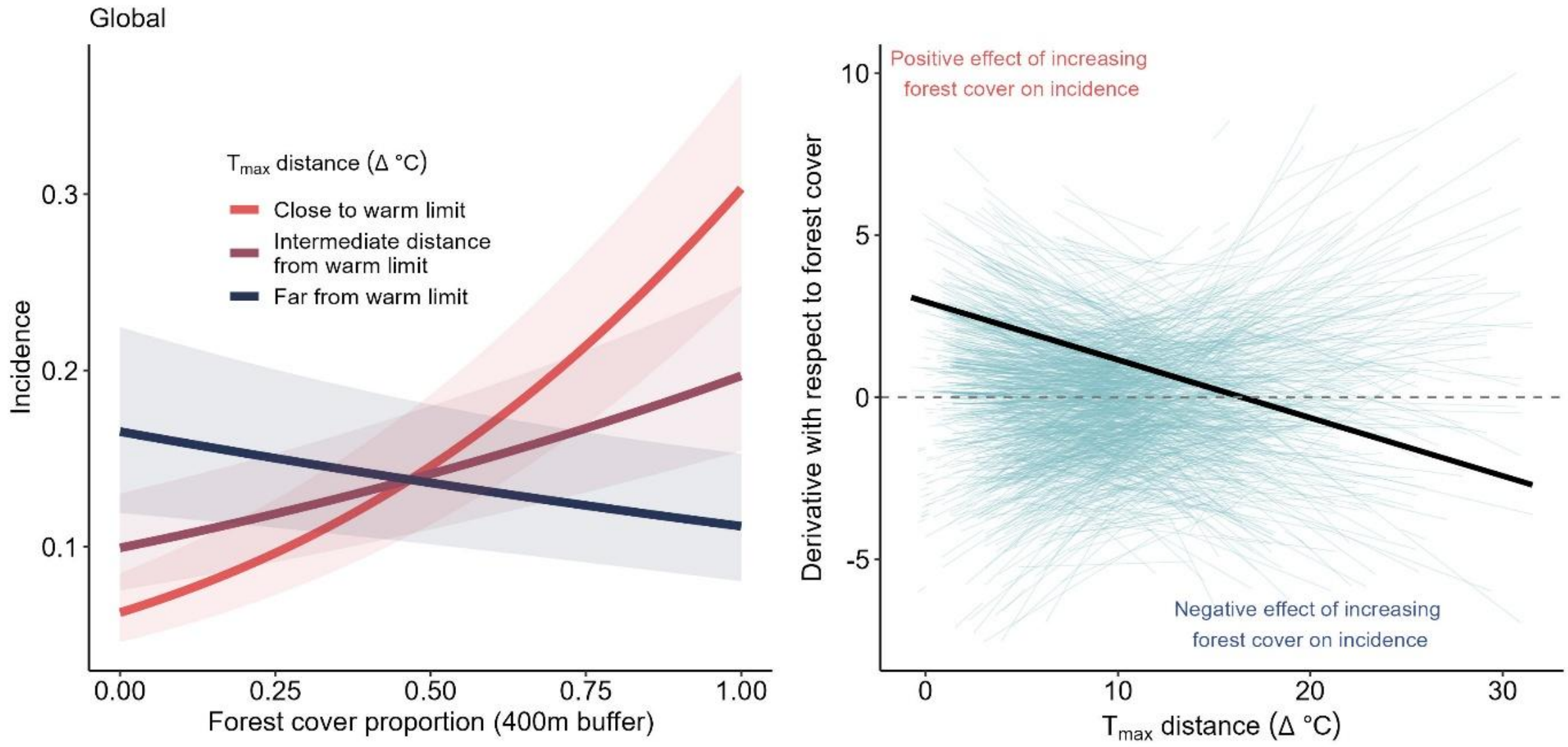

Figure S11. When using a 400m buffer to calculate forest cover, T_{max} distance mediates inter- and intraspecific variation in bird responses to forest cover. *The left panel shows how forest cover proportion within a 600 m radius influences bird incidence across populations at different distances from their species' realised warm tolerance limits (T_{max} distances). The lines represent populations near (pink line, 10th percentile), intermediate (purple, 50th percentile) and far (blue, 90th percentile) from these limits. The shading shows the 95% confidence intervals. The right panel illustrates the within-species variation. Each thin line represents one species and the thick black line shows the overall mean. The y-axis indicates the direction and strength of the response to forest cover, with the grey dotted line marking the point where the response to increasing forest cover changes from negative to positive. Populations closer to their species' realised warm tolerance limits generally show more positive responses to increases in forest cover.*

Appendix 7: Sensitivity test for buffer sizes used to calculate forest cover proportions

Table S28. Model coefficients for model testing how T_{max} distance, forest cover proportion and their interaction affect probability of species incidence. *Forest cover proportion was calculated using a* ***400m buffer*** *around each site (rather than the 600m buffer used in the main analyses). Varying the buffer size did not affect our results.*

**Buffer radius = 400m**				
Predictors	*Log-Odds*	*std. Error*	*z*	*p*
T_{max} distance	0.084	0.014	5.909	**<0.001**
Forest cover proportion	2.949	0.181	16.257	**<0.001**
Interaction between T_{max} distance and forest cover	-0.179	0.016	-11.322	**<0.001**
N_{Site}	7324			
N_{Study}	110			
$N_{Species}$	2272			
Observations	469315			
Marginal R^2 / Conditional R^2	0.02 / 0.71			

Appendix 7: Sensitivity test for buffer sizes used to calculate forest cover proportions

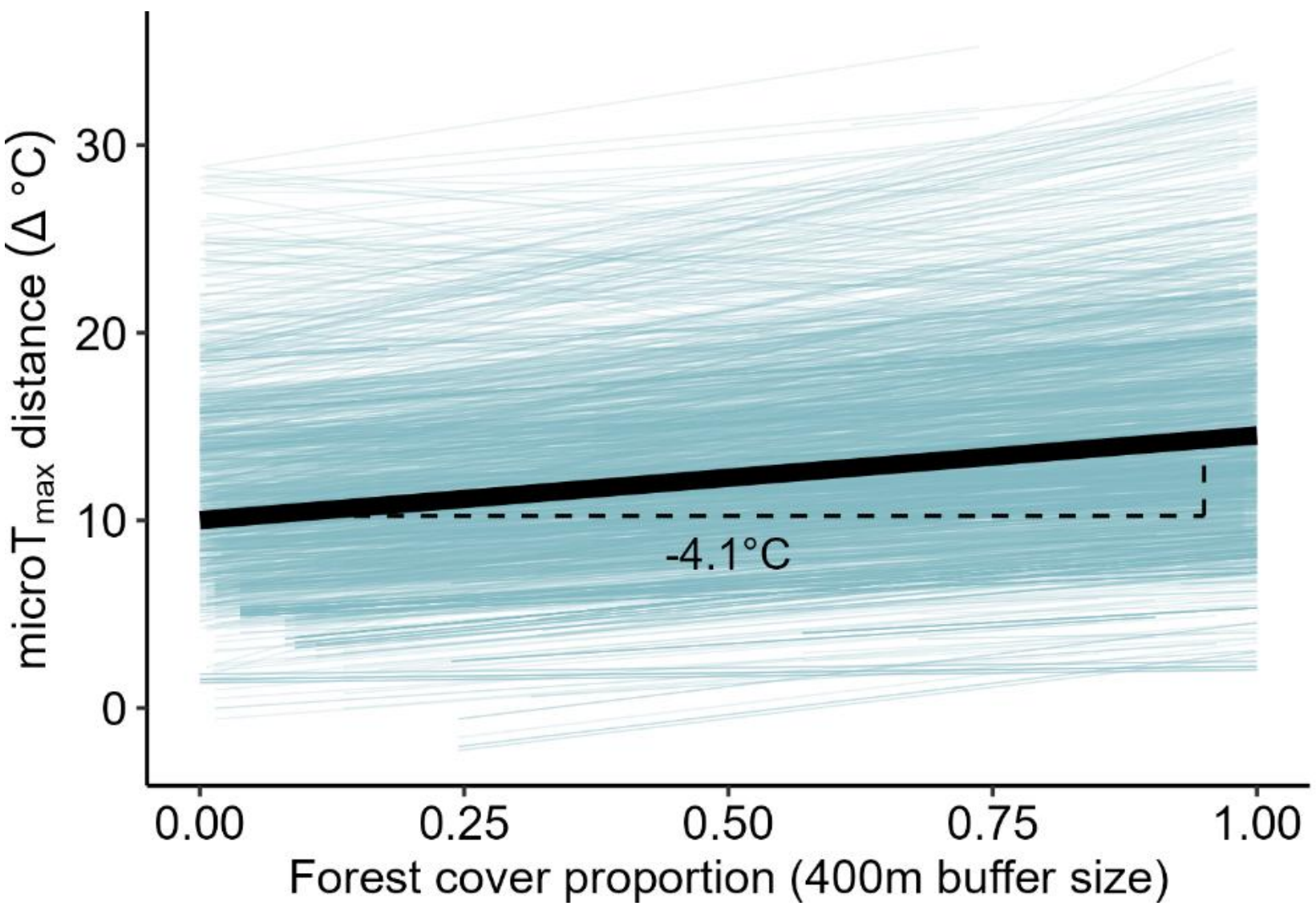

Figure S12. When using a 400m buffer for calculating forest cover proportion, lower forest cover is associated with a reduction in microT$_{max}$ distance. *Lower values of microT$_{max}$ distance indicate microclimatic temperatures closer to a species' warm limit. The thick black line shows the average relationship across all species, and the thin blue lines show the relationships for each species. For a change in forest cover from 95% to 5%, there is a 4.1°C reduction in the average microT$_{max}$ distance.*

Table S29. Linear mixed effect model results for model testing how forest cover affects microT$_{max}$ distance. *Forest cover proportion was calculated using a* ***400m buffer*** *around each site (rather than the 600m buffer used in the main analyses).*

**Buffer radius = 400m**				
Predictors	*Estimates*	*std. Error*	*z*	*p*
Forest cover proportion	4.518	0.079	57.160	**<0.001**
N Species	2272			
Observations	469315			
Marginal R^2 / Conditional R^2	0.08 / 0.84			

Appendix 7: Sensitivity test for buffer sizes used to calculate forest cover proportions

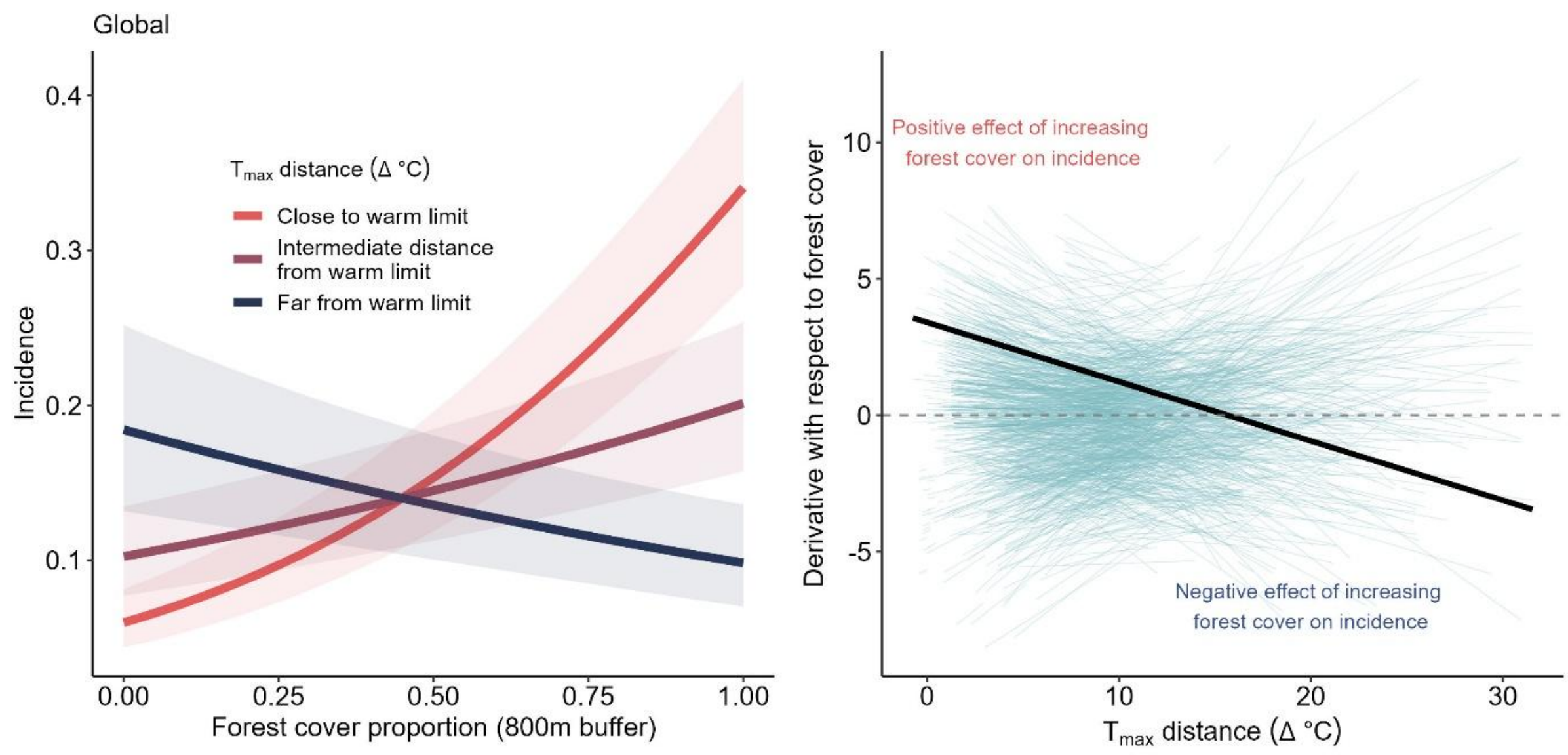

Figure S13. When using an 800m buffer to calculate forest cover, T_{max} distance mediates inter- and intraspecific variation in bird responses to forest cover. *The left panel shows how forest cover proportion within a 600 m radius influences bird incidence across populations at different distances from their species' realised warm tolerance limits (T_{max} distances). The lines represent populations near (pink line, 10th percentile), intermediate (purple, 50th percentile) and far (blue, 90th percentile) from these limits. The shading shows the 95% confidence intervals. The right panel illustrates the within-species variation. Each thin line represents one species and the thick black line shows the overall mean. The y-axis indicates the direction and strength of the response to forest cover, with the grey dotted line marking the point where the response to increasing forest cover changes from negative to positive. Populations closer to their species' realised warm tolerance limits generally show more positive responses to increases in forest cover.*

Appendix 7: Sensitivity test for buffer sizes used to calculate forest cover proportions

Table S30. Model coefficients for model testing how T_{max} distance, forest cover proportion and their interaction affect probability of species incidence. *Forest cover proportion was calculated using an* ***800m buffer*** *around each site (rather than the 600m buffer used in the main analyses). Varying the buffer size did not affect our results.*

**Buffer radius = 800m**				
Predictors	*Log-Odds*	*std. Error*	*z*	*p*
T_{max} distance	0.098	0.015	6.540	**<0.001**
Forest cover proportion	3.399	0.195	17.393	**<0.001**
Interaction between T_{max} distance and forest cover	-0.217	0.017	-12.649	**<0.001**
N_{Site}	7326			
N_{Study}	111			
$N_{Species}$	2279			
Observations	469641			
Marginal R^2 / Conditional R^2	0.02 / 0.72			

Appendix 7: Sensitivity test for buffer sizes used to calculate forest cover proportions

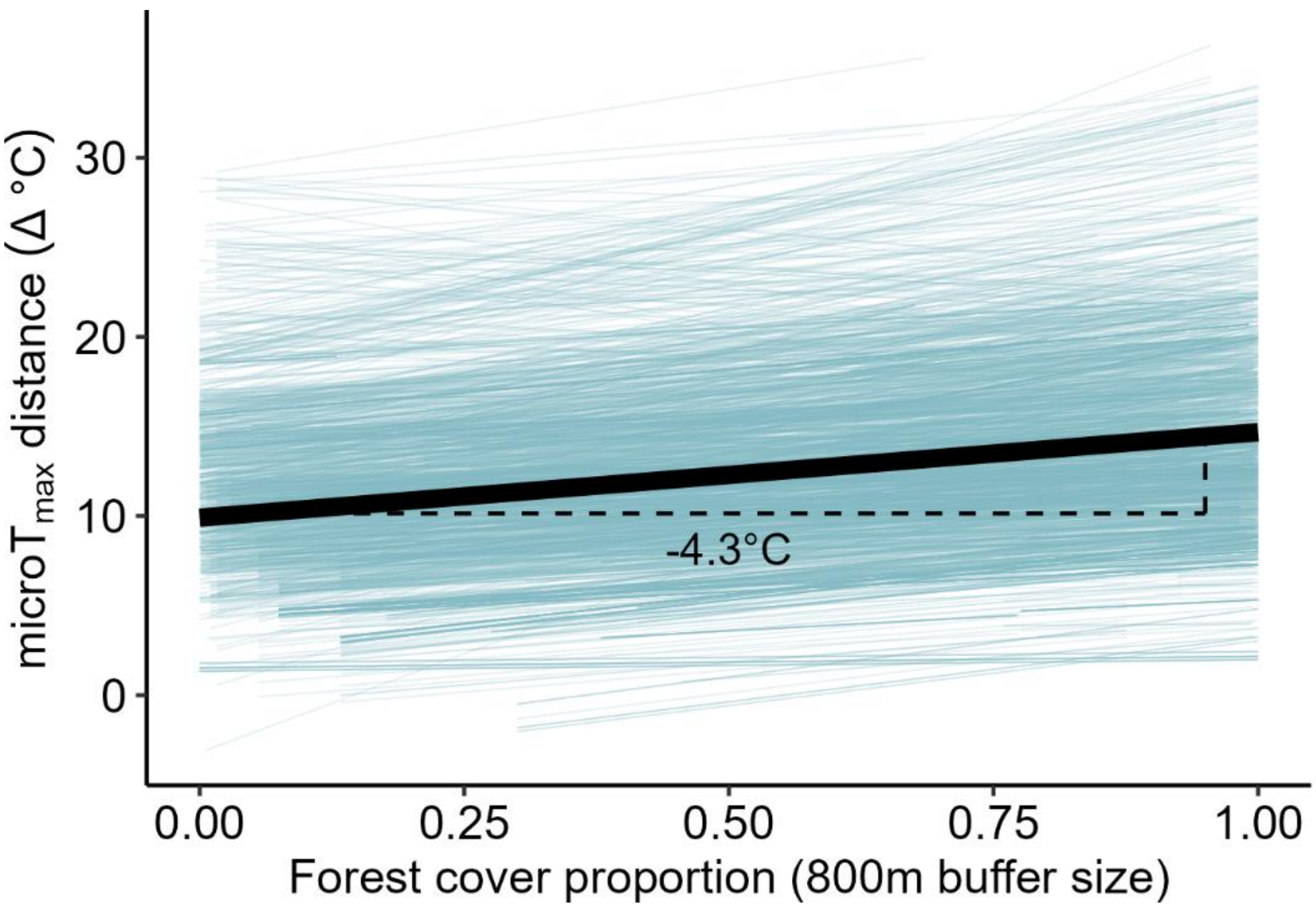

Figure S14. When using an 800m buffer for calculating forest cover proportion, lower forest cover is associated with a reduction in microT$_{max}$ distance. *Lower values of microT$_{max}$ distance indicate microclimatic temperatures closer to a species' warm limit. The thick black line shows the average relationship across all species, and the thin blue lines show the relationships for each species. For a change in forest cover from 95% to 5%, there is a 4.3°C reduction in the average microT$_{max}$ distance.*

Table S31. Linear mixed effect model results for model testing how forest cover affects microT$_{max}$ distance. *Forest cover proportion was calculated using an **800m buffer** around each site (rather than the 600m buffer used in the main analyses).*

**Buffer radius = 800m**				
Predictors	*Estimates*	*std. Error*	*z*	*p*
Forest cover proportion	4.772	0.097	49.217	**<0.001**
N $_{Final\_name}$	2279			
Observations	469641			
Marginal R^2 / Conditional R^2	0.08 / 0.84			

Appendix 8: Sensitivity test for calculating T_{max} using the 95th percentile of the maximum monthly temperatures experienced within the species' range

In the main manuscript, we estimated each species' realised warm tolerance limit by finding the maximum of the hottest monthly temperatures experienced within the species' geographic range. This could potentially be affected by anomalously high temperatures within species ranges. There is also a possibility that organisms could try to avoid exposure to the hottest macroclimatic temperatures within their geographic ranges, for instance by adjusting their distribution within their range and dispersing to cooler parts of their range, or by dwelling only within thermally buffered microhabitats when located within the hottest part of their range [10,11].

To test whether this could have affected our results, we calculated species' realised warm tolerance limits (T_{max}) using the 95th percentile (rather than the maximum) of the hottest monthly temperatures experienced within the species' geographic range. This provides a lower estimate of the warm limit which reduces the potential effects of outliers and species' use of buffered microhabitats within their range. As expected, this made the maximum *T_{max} distance* and *microTmax distance* generally lower. However, it had no effect on the results.

Appendix 8: Sensitivity test for calculating T_{max} using the 95th percentile of the maximum monthly temperatures experienced within the species' range

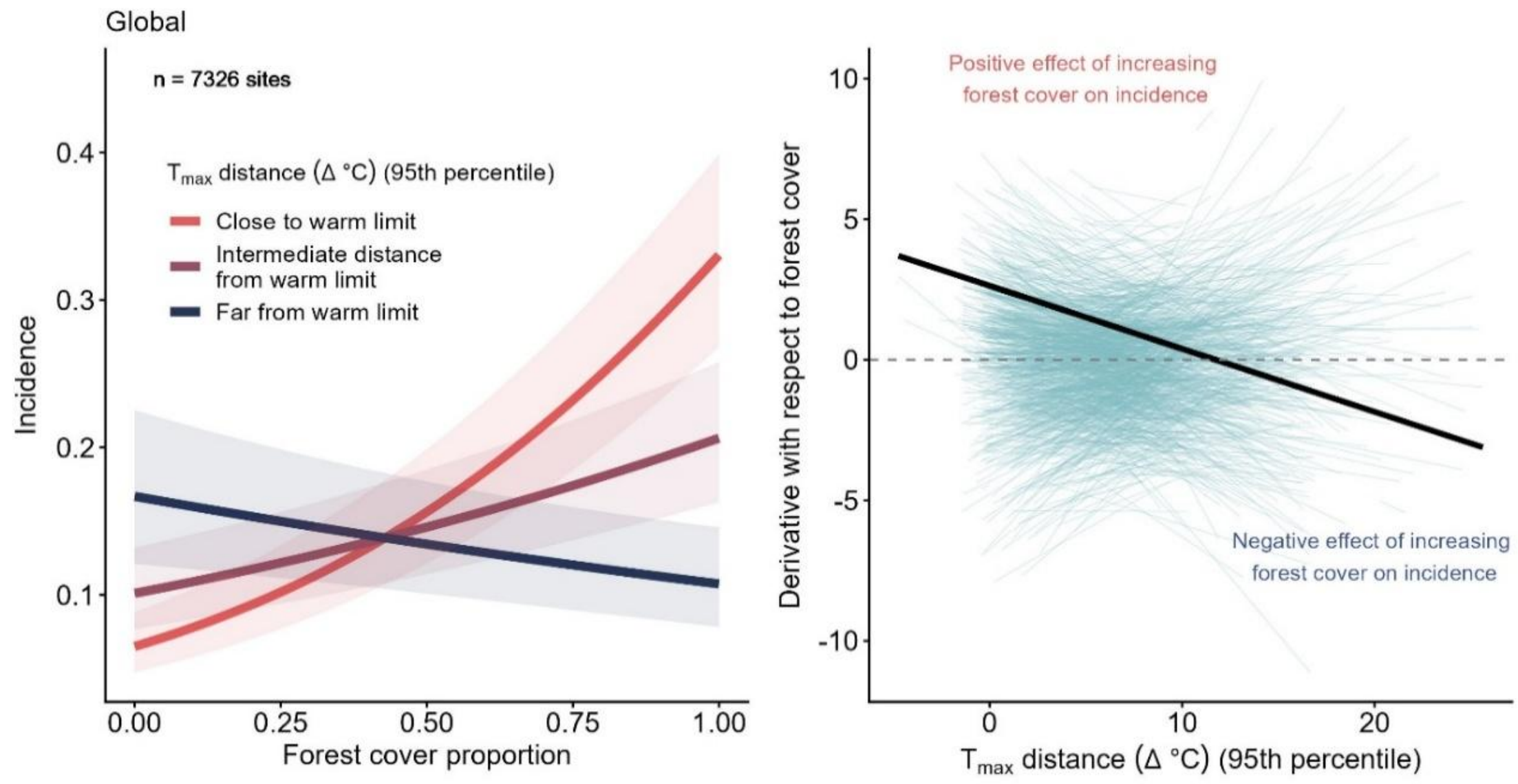

Figure S15. When calculating T_{max} distance using the 95th percentile of the maximum monthly temperatures within the species' range, T_{max} distance mediates inter- and intraspecific variation in bird responses to forest cover. *The left panel shows how forest cover proportion within a 600 m radius influences bird incidence across populations at different distances from their species' realised warm tolerance limits (T_{max} distances). The lines represent populations near (pink line, 10th percentile), intermediate (purple, 50th percentile) and far (blue, 90th percentile) from these limits. The 10th percentile of T_{max} distance is 3°C from the warm limit, 50th percentile is 8°C and 90th percentile of 14°C. The shading shows the 95% confidence intervals. The right panel illustrates the within-species variation. Each thin line represents one species and the thick black line shows the overall mean. The y-axis indicates the direction and strength of the response to forest cover, with the grey dotted line marking the point where the response to increasing forest cover changes from negative to positive. Populations closer to their species' realised warm tolerance limits generally show more positive responses to increases in forest cover.*

Appendix 8: Sensitivity test for calculating T_{max} using the 95th percentile of the maximum monthly temperatures experienced within the species' range

Table S32. Binomial GLMM results for model testing how T_{max} distance (based on 95% quantiles), forest cover and their interaction affect probability of incidence.

**Incidence ~ T_{max} distance * forest cover + (1|Study / Site) + (T_{max} distance * forest cover | Species)**				
Predictors	*Log-Odds*	*std. Error*	*z*	*p*
Distance to warm limit	0.096	0.016	5.847	**<0.001**
Forest cover proportion	2.633	0.156	16.903	**<0.001**
Interaction between distance to warm limit and forest cover	-0.224	0.019	-11.752	**<0.001**
N_{Site}	7326			
N_{Study}	111			
$N_{Species}$	2262			
Observations	467333			
Marginal R^2 / Conditional R^2	0.02 / 0.69			
AIC	294585			

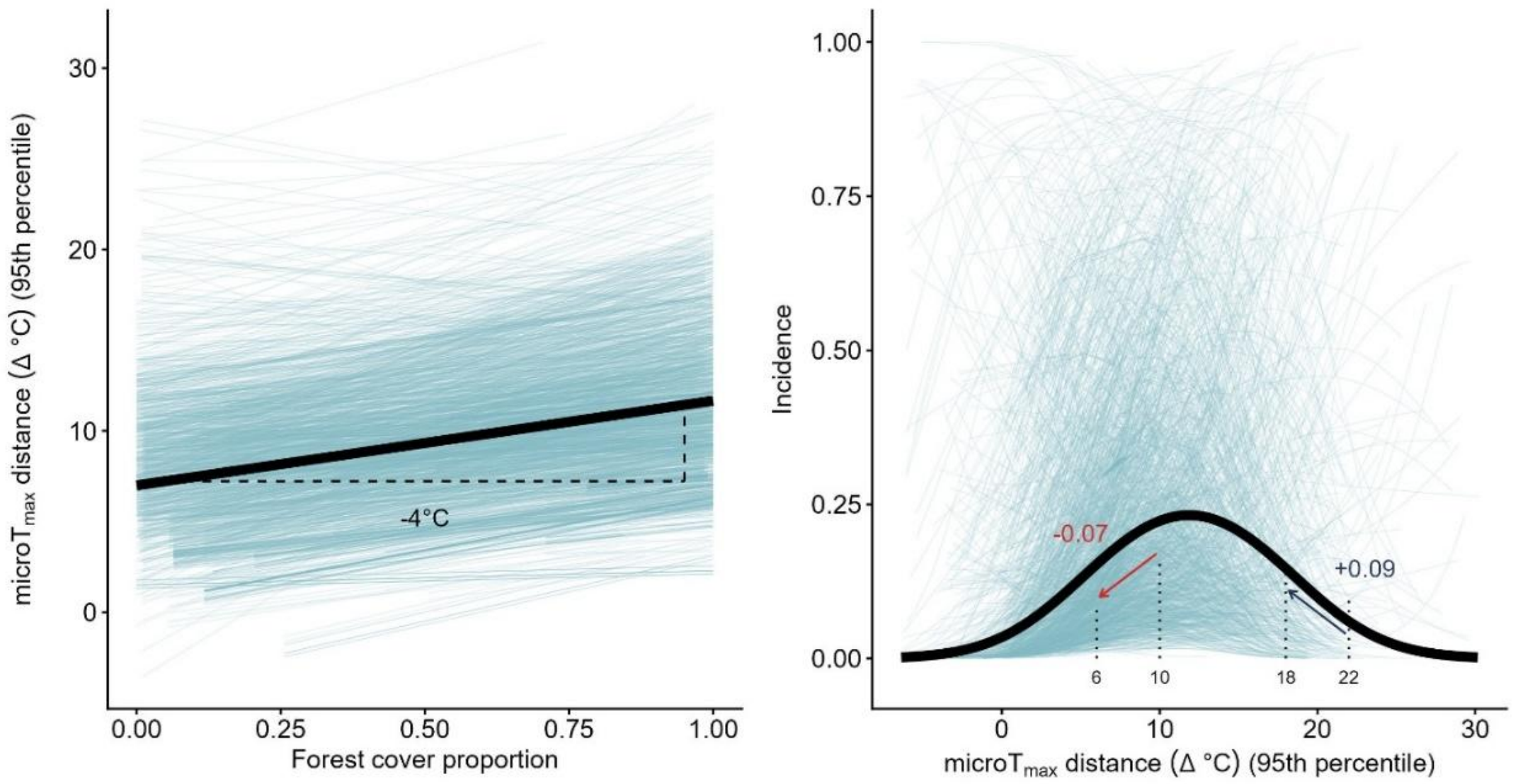

Figure S16. When calculating T_{max} distance using the 95th percentile of the maximum monthly temperatures within the species' range, sites at low forest cover are closer to species' warm limits and this microclimatic shift canaffect populations differently depending on the macroclimate in which they are located. *The left panel shows how lower forest cover is associated with a reduction in microT$_{max}$ distance. Lower values of microT$_{max}$ distance indicate microclimatic temperatures closer to a species' warm limit. The thick black*

line shows the average relationship across all species, and the thin blue lines show the relationships for each species. For a change in forest cover from 95% to 5%, there is a 4°C reduction in the average microT_{max} distance. The right panel shows the effect of microT_{max} distance on incidence. The thick black line shows the overall relationship, and the thin blue lines show the relationships for each species. We annotate an example of how a 4°C decrease in microT_{max} distance can have contrasting effects on species' incidence depending on the starting microT_{max} distance.

Table S33. Linear mixed effect model results for model testing how forest cover affects microT_{max} distance (based on 95% quantiles).

**microT_{max} distance ~ Forest cover proportion + (Forest cover proportion | Species)**				
Predictors	*Estimates*	*std. Error*	*z*	*p*
Forest cover proportion	4.670	0.088	52.919	**<0.001**
$N_{Species}$	2262			
Observations	467333			
Marginal R^2 / Conditional R^2	0.11 / 0.77			

Table S34. Binomial GLMM results for model testing how microT_{max} distance affects probability of incidence (based on 95% quantiles).

**Incidence ~ microT_{max} distance^2 + (1 |Study/Site) + (microT_{max} distance^2 | Species)**				
Predictors	*Log-Odds*	*std. Error*	*z*	*p*
microT_{max} distance [1st degree]	0.359	0.023	15.804	**<0.001**
microT_{max} distance [2nd degree]	-0.015	0.001	-13.652	**<0.001**
N_{Site}	7326			
N_{Study}	111			
$N_{Species}$	2262			
Observations	467333			
Marginal R^2 / Conditional R^2	0.01 / 0.80			
AIC	299323			

Appendix 9: Comparison of maximum temperature of the warmest month (*BIO05*) in the year 2000 with long-term climate average

We used temperature data from the year 2000 as it aligned with the forest cover data. Since the impacts of warm temperatures can persist over time [12], and the studies in our dataset were conducted during or after 2000, we expect species in these studies will have been influenced by temperature in 2000. Although studies conducted in later years may have experienced different absolute temperatures to those in 2000, the relative difference in temperature between forested and non-forested sites is not expected to change, allowing us to assess how temperature affects responses to forest cover.

Since the maximum monthly temperature (*BIO05*) may show inter-annual variations, we tested how closely the *BIO05* temperature in 2000 approximates the longer-term 40-year climate average of this variable. We downloaded the average *BIO05* temperature between 1979-2018 at 0.5° resolution from the ERA5 Bioclimatic Indicators dataset[13], and compared this with the *BIO05* temperature in 2000 at 0.5° resolution also from the ERA5 Bioclimatic Indicators dataset. We extracted the values of both variables for our study sites and found that the two variables were highly correlated (correlation coefficient = 0.97, Figure S17), indicating that the *BIO05* temperature in the year 2000 is a close approximation of the longer-term average.

Appendix 9: Comparison of maximum temperature of the warmest month (*BIO05*) in the year 2000 with long-term climate average

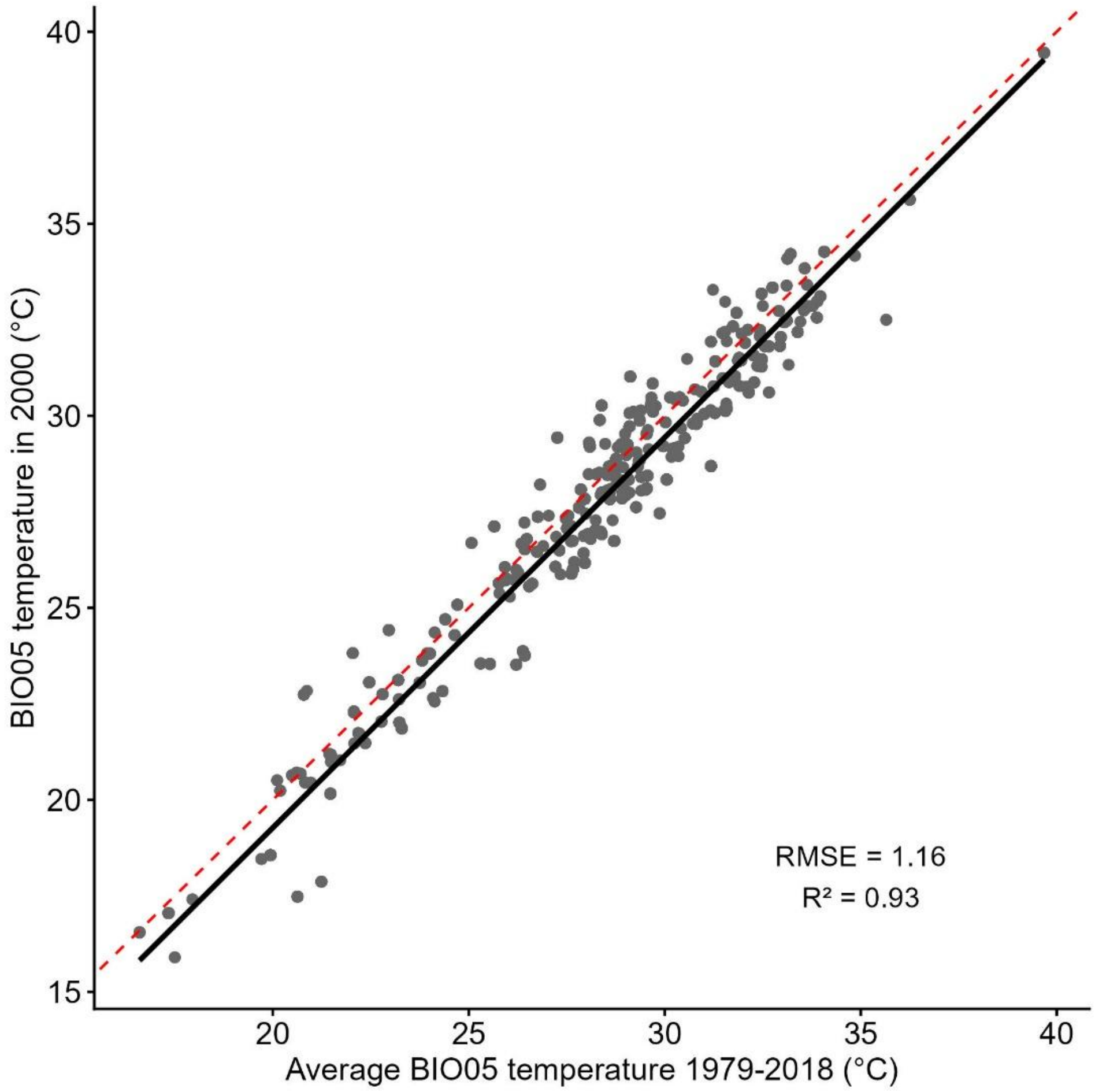

Figure S17. Comparison of maximum temperature of the warmest month (**BIO05*****) in the year 2000 with the 40-year average of this variable between 1979-2018 for the locations in our study.*** *The temperatures were extracted from the Bioclimatic Indicators dataset which is derived from ERA5 reanalysis and provided maps of* BIO05 *at 0.5° gridded resolution. Each grey point in the figure represents one sample site (n = 7326). Most sample sites within the same study have the same BIO05 temperature at 0.5 ° resolution as they are located within the same grid cell. The black line shows a linear regression between the average* BIO05 *temperature between 1979-2018 and the* BIO05 *temperature in 2000, and the dotted red line shows the theoretical 1:1 relationship between these two variables. The R^2 of the linear regression and the root-mean-square-error between the two variables are annotated to quantify similarity between the two variables.*

Appendix 10: Microclimate model validation

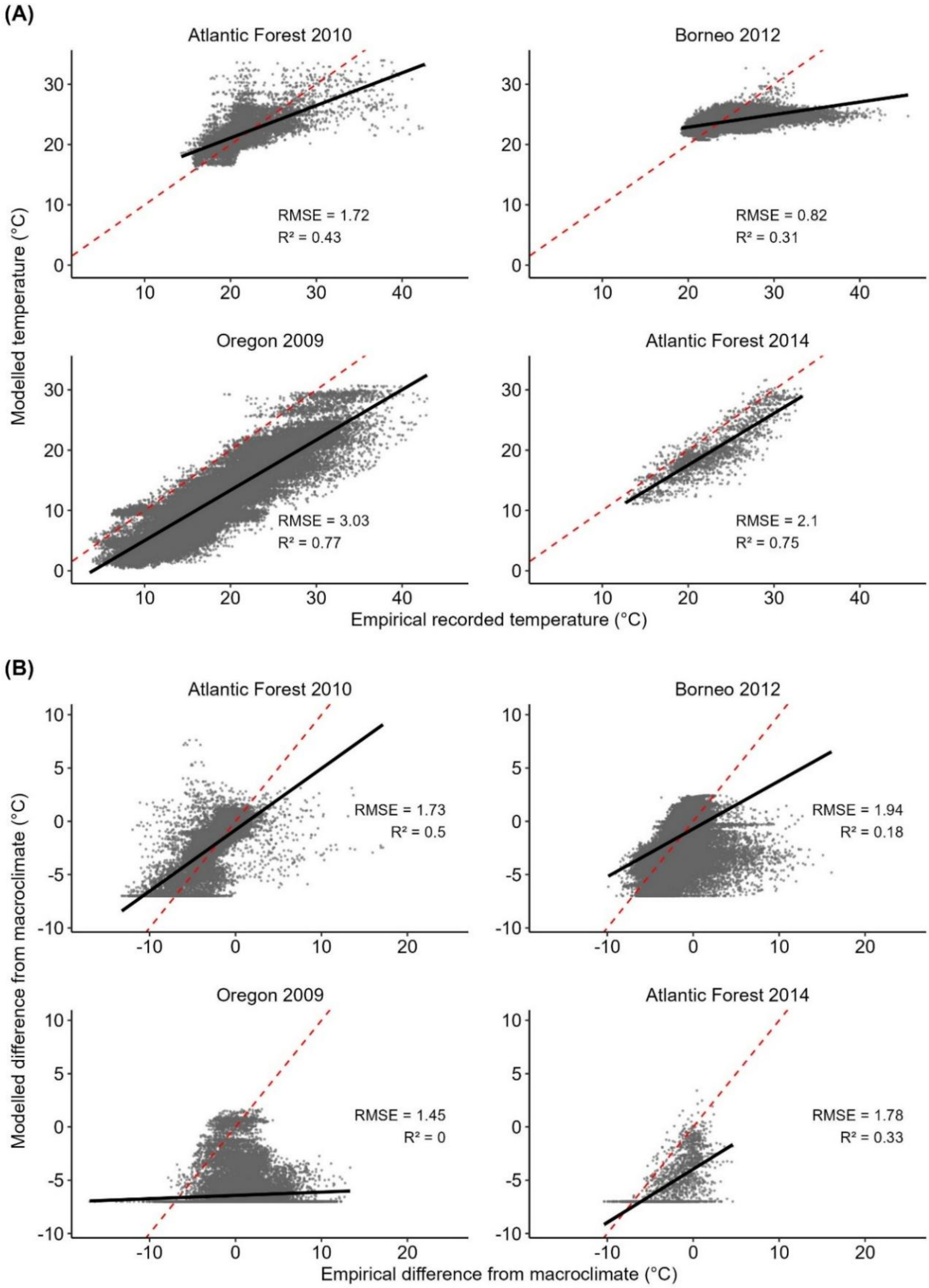

Figure S18. Comparison of modelled and empirical microclimate temperatures from four different datasets. *In panel (A) the y-axis shows the temperatures modelled using* microclimc

and the x-axis shows temperatures recorded using in situ temperature loggers. In panel (B) the y-axis shows the temperature difference between the macroclimate and the temperatures modelled using microclimc, *and the x-axis shows the temperature difference between the macroclimate and the temperatures recorded using in situ temperature loggers. Grey dots represent individual hourly temperature measurements. Black lines show linear regressions between the empirical and modelled temperatures, and dotted red lines show the theoretical 1:1 relationship. The root mean square error (RMSE) and* R^2 *for each dataset are annotated on the figure to quantify model performance.*

Appendix 11: Timespan of studies

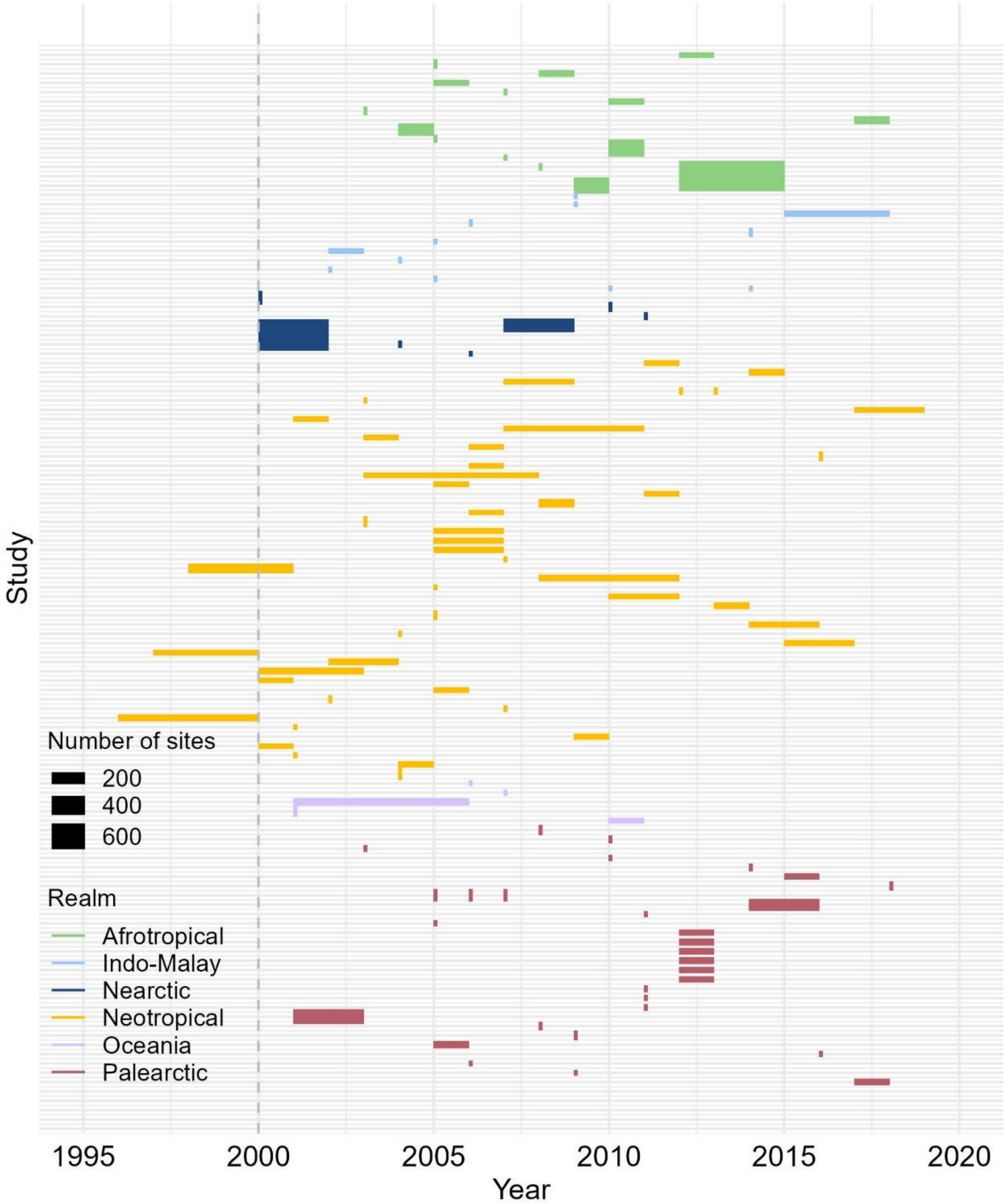

Figure S19. Temporal distribution of studies included in the dataset. *Each horizontal row represents one study. The position and length of each coloured bar on the x-axis indicate the year(s) during which the study was conducted. The height of the bars represents the number of sites within the study.*

References

1. Wilman, H. *et al.* EltonTraits 1.0: Species-level foraging attributes of the world's birds and mammals. *Ecology* **95**, 2027–2027 (2014).
2. McTavish, E. J. *et al.* A complete and dynamic tree of birds. *Proc. Natl. Acad. Sci.* **122**, e2409658122 (2025).
3. Orme, D. *et al.* caper: Comparative Analyses of Phylogenetics and Evolution in R. (2023).
4. Williams, C. *et al.* Fast phylogenetic generalised linear mixed-effects modelling using the glmmTMB R package. 2025.12.20.695312 Preprint at https://doi.org/10.64898/2025.12.20.695312 (2026).
5. Brooks, M. *et al.* glmmTMB: Generalized Linear Mixed Models using Template Model Builder. (2024).
6. Betts, M. G. *et al.* Extinction filters mediate the global effects of habitat fragmentation on animals. *Science* **366**, 1236–1239 (2019).
7. Weeks, T. L. *et al.* Climate-driven variation in dispersal ability predicts responses to forest fragmentation in birds. *Nat. Ecol. Evol.* **7**, 1079–1091 (2023).
8. Hua, F. *et al.* Ecological filtering shapes the impacts of agricultural deforestation on biodiversity. *Nat. Ecol. Evol.* **8**, 251–266 (2024).
9. IUCN. The IUCN Red List of Threatened Species. *IUCN Red List of Threatened Species* https://www.iucnredlist.org/en (2024).
10. Cohen, J. M. & Jetz, W. Geographic redistributions are insufficient to mitigate exposure to climate change in North American birds. *Nat. Ecol. Evol.* **9**, 1234–1244 (2025).
11. Cohen, J. M., Fink, D. & Zuckerberg, B. Spatial and seasonal variation in thermal sensitivity within North American bird species. *Proc. R. Soc. B Biol. Sci.* **290**, 20231398 (2023).
12. León, G. M.-D. & Thakur, M. P. Ecological debts induced by heat extremes. *Trends Ecol. Evol.* **39**, 1024–1034 (2024).
13. Copernicus Climate Change Service. Global bioclimatic indicators from 1979 to 2018 derived from reanalysis. ECMWF https://doi.org/10.24381/CDS.BCE175F0 (2021).
