# Supplementary material for: Temperature mediates variation in avian sensitivity to forest cover
Source: Nat Ecol Evol. 2026 Aug 28;10(10):1914–26. doi: 10.1038/s41559-026-03160-9 (PMC13642040; doi:10.1038/s41559-026-03160-9)
Supplement: Supplementary file 2 [file 41559_2026_3160_MOESM2_ESM.pdf]

nature portfolio

Corresponding author(s): Natasha Granville

Last updated by author(s): Jun 19, 2026

Reporting Summary

Nature Portfolio wishes to improve the reproducibility of the work that we publish. This form provides structure for consistency and transparency in reporting. For further information on Nature Portfolio policies, see our Editorial Policies and the Editorial Policy Checklist.

Statistics

For all statistical analyses, confirm that the following items are present in the figure legend, table legend, main text, or Methods section.

n/a	Confirmed	
☐	☒	The exact sample size (*n*) for each experimental group/condition, given as a discrete number and unit of measurement
☐	☒	A statement on whether measurements were taken from distinct samples or whether the same sample was measured repeatedly
☐	☒	The statistical test(s) used AND whether they are one- or two-sided<br>*Only common tests should be described solely by name; describe more complex techniques in the Methods section.*
☐	☒	A description of all covariates tested
☐	☒	A description of any assumptions or corrections, such as tests of normality and adjustment for multiple comparisons
☐	☒	A full description of the statistical parameters including central tendency (e.g. means) or other basic estimates (e.g. regression coefficient) AND variation (e.g. standard deviation) or associated estimates of uncertainty (e.g. confidence intervals)
☐	☒	For null hypothesis testing, the test statistic (e.g. *F*, *t*, *r*) with confidence intervals, effect sizes, degrees of freedom and *P* value noted<br>*Give P values as exact values whenever suitable.*
☒	☐	For Bayesian analysis, information on the choice of priors and Markov chain Monte Carlo settings
☒	☐	For hierarchical and complex designs, identification of the appropriate level for tests and full reporting of outcomes
☐	☒	Estimates of effect sizes (e.g. Cohen's *d*, Pearson's *r*), indicating how they were calculated

Our web collection on statistics for biologists contains articles on many of the points above.

Software and code

Policy information about availability of computer code

Data collection	We used the following openly available R packages to download data: gfcanalysis v1.8.0 (to download Hansen tree cover data), luna v0.3.7 (to download MODIS Leaf Area Index data), mcera5 v1.0 (to download ERA5 climate data for the microclimate model), microclima v0.1.0 (to download digital elevation rasters).
Data analysis	We used the following openly available R packages for data processing, modelling and analysis:<br>caper v1.0.4, cowplot v1.2.0, DHARMa v0.4.7, emmeans v2.0.0, fasterize v1.1.0, ggeffects v2.3, glmmTMB v1.1.13, landscapemetrics v2.2.1, microclimc v0.1.0, predictsFunctions v1.0, sf v1.0.22, sjPlot v2.9.0, terrra v1.8.80, tidyverse v2.0.0, units v1.0.0, weathermetrics v1.2.2.<br>We used R v4.3.1 to download the elevation data using the get_dem function from the microclima package and to run the microclimate model using the microclimc package. We used R v4.4.1 for all other analyses.<br>All R code used for this study is openly available on GitHub: https://github.com/natashagranville/Warm_Limits

For manuscripts utilizing custom algorithms or software that are central to the research but not yet described in published literature, software must be made available to editors and reviewers. We strongly encourage code deposition in a community repository (e.g. GitHub). See the Nature Portfolio guidelines for submitting code & software for further information.

Data

Policy information about availability of data

All manuscripts must include a data availability statement. This statement should provide the following information, where applicable:

- Accession codes, unique identifiers, or web links for publicly available datasets
- A description of any restrictions on data availability
- For clinical datasets or third party data, please ensure that the statement adheres to our policy

The PREDICTS Project database can be downloaded from: https://data.nhm.ac.uk/dataset/the-2016-release-of-the-predicts-database-v1-1 and https://data.nhm.ac.uk/dataset/release-of-data-added-to-the-predicts-database-november-2022.
The BIOFRAG data can be requested from: https://biofrag.wordpress.com/biofrag-measuring-biodiversity-response-to-forest-fragmentation/.
The Atlantic Bird data can be downloaded from: http://onlinelibrary.wiley.com/doi/10.1002/ecy.2119/suppinfo.
The global tree cover maps used for estimating forest cover can be downloaded from: https://storage.googleapis.com/earthenginepartners-hansen/GFC-2022-v1.10/download.html.
The hourly temperature maps used for calculating Tmax distance can be downloaded from: https://cds.climate.copernicus.eu/datasets/reanalysis-era5-single-levels?tab=download.
The species' range maps used for estimating species' warm limits can be requested from BirdLife International via: http://datazone.birdlife.org/species/requestdis.
The hourly climate data used to drive the microclimate model can be downloaded from: https://cds.climate.copernicus.eu/datasets/reanalysis-era5-single-levels?tab=download.
The environmental parameters used in the microclimate model include: (1) Leaf Area Index: https://lpdaac.usgs.gov/products/mod15a2hv006/. (2) Canopy height: https://glad.umd.edu/dataset/GLCLUC2020. (3) Habitat types: https://lpdaac.usgs.gov/products/mcd12q1v061/. (4) Digital elevation: https://registry.opendata.aws/terrain-tiles/. (5) Soil types: https://doi.org/10.5281/zenodo.2525817.

Research involving human participants, their data, or biological material

Policy information about studies with human participants or human data. See also policy information about sex, gender (identity/presentation), and sexual orientation and race, ethnicity and racism.

Reporting on sex and gender	N/A
Reporting on race, ethnicity, or other socially relevant groupings	N/A
Population characteristics	N/A
Recruitment	N/A
Ethics oversight	N/A

Note that full information on the approval of the study protocol must also be provided in the manuscript.

Field-specific reporting

Please select the one below that is the best fit for your research. If you are not sure, read the appropriate sections before making your selection.

☐ Life sciences ☐ Behavioural & social sciences ☒ Ecological, evolutionary & environmental sciences

For a reference copy of the document with all sections, see nature.com/documents/nr-reporting-summary-flat.pdf

Ecological, evolutionary & environmental sciences study design

All studies must disclose on these points even when the disclosure is negative.

Study description	This study investigates how the proximity of bird populations to their species' warm range limits affects their responses to forest cover. To do this, we compiled bird presence-absence data from the PREDICTS, BIOFRAG and Atlantic Bird databases. Our dataset consists of bird assemblages surveyed at sites which are nested within studies. The dataset includes a total of 2,262 bird species sampled across 7,326 sites within 111 studies, spanning all forested continents. We analysed how forest cover, distance to warm limit and their interaction affect bird probability of presence (incidence).<br>Next, to provide insights into the potential processes driving variability in population responses, we examined how forest cover impacts the local microclimatic temperatures that species experience. We modelled the microclimatic temperatures at each site using the openly available R package microclimc. We then used mixed effects models to investigate how these microclimatic temperatures affect species incidence.
Research sample	We chose to focus our study on birds because their distributions are well-characterised and there is a breadth of existing survey data for bird communities. We obtained bird presence-absence data from the PREDICTS, BIOFRAG and Atlantic Bird databases. The

	PREDICTS Project database collates data from published studies of ecological assemblages across varying human-modified land uses. We used studies on bird assemblages within forest ecoregions. The BIOFRAG database collates primary datasets of ecological assemblages sampled at sites within fragmented forests. The Atlantic Birds database reports bird species' relative abundances within studies across the Brazilian Atlantic Forest.
Sampling strategy	We included all studies from the BIOFRAG, PREDICTS and Atlantic Bird databases that met all of the following criteria: (1) study conducted on birds. (2) study located within a forest ecoregion. (3) >1 site within the study. (4) study conducted during or after the year 2000. Where there were temporal duplicates of a study (i.e., repeated surveys of the same locations in different years), we kept the study which was conducted in the year closest to 2000 to avoid pseudoreplication. The dataset consists of 2,262 bird species across 7,326 sites within 111 studies.
Data collection	Data were obtained from previously published sources and online databases. Microclimate estimates were obtained by microclimate modelling using the freely available microclimc R package to model temperatures in hourly increments at 2m above ground in the hottest month in the year 2000.
Timing and spatial scale	The dataset includes studies from all forested continents. Bird assemblage data were collected between 1996 and 2019. The timespan of each study is shown in Table S1. Forest cover, macroclimate and microclimate estimates were obtained for the year 2000.
Data exclusions	To minimise the impact of potential taxonomic issues or outdated range maps, we removed records of species present >200 km outside their geographic ranges (range maps obtained from BirdLife) using pre-established criteria: if a species was recorded as present >200 km outside its range in one study, we removed the species from that study. If a species was recorded as present >200 km outside its range in multiple studies, we removed that species from analysis entirely.
Reproducibility	All code used for data processing, modelling, analysis and construction of figures is freely available on GitHub: https://github.com/natashagranville/Warm_Limits.<br>The results are fully reproducible using the code and the data which can be obtained from the links in the data availability statement.
Randomization	Randomization is not possible for ths study as all data are from previously collected and published sources. We used random effects in our mixed-effect models to control for variation in sampling methods and detection probabilities across studies, as well as variation in responses across species (please see Methods for more details on the statistical models).
Blinding	Blinding was not relevant to this study as all data were from previously collected and published sources.

Did the study involve field work? ☐ Yes ☒ No

Reporting for specific materials, systems and methods

We require information from authors about some types of materials, experimental systems and methods used in many studies. Here, indicate whether each material, system or method listed is relevant to your study. If you are not sure if a list item applies to your research, read the appropriate section before selecting a response.

Materials & experimental systems

n/a	Involved in the study
☒	☐ Antibodies
☒	☐ Eukaryotic cell lines
☒	☐ Palaeontology and archaeology
☒	☐ Animals and other organisms
☒	☐ Clinical data
☒	☐ Dual use research of concern
☒	☐ Plants

Methods

n/a	Involved in the study
☒	☐ ChIP-seq
☒	☐ Flow cytometry
☒	☐ MRI-based neuroimaging

Plants

Seed stocks	N/A
Novel plant genotypes	N/A
Authentication	N/A
