# Supplementary material for: Temperature mediates variation in avian sensitivity to forest cover
Source: Nat Ecol Evol. 2026 Aug 28;10(10):1914–26. doi: 10.1038/s41559-026-03160-9 (PMC13642040; doi:10.1038/s41559-026-03160-9)
Supplement: Supplementary file 3 [file 41559_2026_3160_MOESM3_ESM.pdf]

nature portfolio

Peer Review File

Temperature mediates variation in avian sensitivity to forest cover

Corresponding Author: Ms Natasha Granville

Version 0:

Reviewer comments:

Reviewer #1

(Remarks to the Author)
The authors have produced an ambitious and thoughtfully constructed manuscript that I enjoyed reading. The integration of macroclimatic thermal position, fine-scale microclimatic buffering, and global bird community data to explain variation in avian responses to forest cover is conceptually elegant. The central idea, whereby proximity to a species' realised warm limit mediates both inter- and intraspecific sensitivity to forest cover, is clearly supported by the analyses. Overall, I congratulate the authors on an exciting and well written manuscript and consider this work a strong fit for Nature Ecology & Evolution.

Major comments
The manuscript defines each species' warm limit (Tmax) as the highest value of BIO05 (mean daily maximum temperature of the warmest month) experienced anywhere within the species' BirdLife range polygon, using ERA5 in 2000. This is appropriately framed as a realised warm tolerance inferred from distribution rather than a physiological limit. However, because Tmax is calculated across entire range polygons using coarse macroclimate grids, it is best interpreted as the upper envelope of regional macroclimatic conditions associated with the mapped range, rather than temperatures necessarily experienced in the forest microhabitats where many populations occur. The authors do acknowledge this issue directly (i.e., that Tmax distance based on site macroclimate may not represent temperatures birds experience inside forests) and the microclimate modelling is done on this basis. It would be helpful to briefly discuss that some species may not occupy the hottest parts of their mapped range (or may persist there only in buffered microhabitats). The Appendix 7 sensitivity test (95th percentile Tmax) addresses potential outliers within ranges, but not habitat-specific exposure; acknowledging this would improve interpretability.

Please clarify how the Tmax-distance interpretation applies to species whose ranges span relatively few ERA5 grid cells or limited climatic variation. In such cases, Tmax may reflect restricted climatic breadth. The manuscript currently emphasises generality across traits, but range breadth is an important separate axis.

The microclimate component is central to the mechanistic interpretation, and the results explicitly rely on it to argue that forest cover alters microclimatic thermal exposure and thus mediates incidence responses. I have two suggestions here:
1) The model is validated against four logger datasets. However, I suggest that the authors provide a bit more detail on logger placement (height above ground and forest cover/type) for each validation dataset. This is important because the simulations are conducted at 2 m above ground, and "above-ground in situ" could still encompass a range of placements that affect comparability.
2) Because mechanistic microclimate modelling is a major element of the analyses, I recommend introducing it more explicitly in the introduction (even briefly). As written, the reader could form an early impression that the study is purely macroclimate-driven before encountering the valuable mechanistic component.

The manuscript justifies using the year 2000 to align with the forest cover data and argues that relative forest/non-forest temperature differences are not expected to change, even if absolute temperatures differ in later survey years. However, because BIO05 captures peak seasonal heat (mean daily maximum in the warmest month), it may be more sensitive to interannual variability than broader metrics like mean annual temperature. I suggest adding a brief statement on whether 2000 was checked against a longer-term climate for BIO05. This point is particularly relevant because the interpretation hinges on "distance to warm limits," which could shift with year-to-year extremes.

Figure 2C shows substantial interspecific variation in the species-specific effects of forest cover across Tmax distance, with many species exhibiting slopes opposite to the overall mean trend. The manuscript does acknowledge that results identify general trends but do not reveal underlying processes. It would be useful to explicitly quantify this heterogeneity (e.g.,

proportion of species with positive vs negative forest sensitivity at low vs high Tmax distance; or distribution of species-level interaction terms). This would clarify whether the key inference is best viewed as a shift in the average response across species/populations rather than a rule that applies to most species.

The manuscript reports that the Tmax distance × forest cover interaction varies among biogeographic realms, being weakest in Australasia, and some realms have notably fewer sites (e.g., Australasia, Indo-Malaya). This makes it difficult to distinguish genuine biological differences from reduced statistical power and uneven sampling. In addition, realms differ substantially in internal climatic and habitat heterogeneity (e.g., Afrotropical encompasses Mediterranean, montane, and tropical systems), which may influence both the distribution of Tmax distances and the strength of forest buffering. Please clarify these limitations and consider summarising how Tmax distance (and its variance) is distributed within and among realms, to support interpretation of realm-level differences.

The manuscript links results to a latitudinal narrative (low latitudes being closer to warm limits). While plausible, latitude is not directly analysed, and realms contain substantial internal heterogeneity. I recommend either (i) adding an explicit latitude/biome analysis, or (ii) softening the wording so that the latitudinal mechanism is framed more clearly as a hypothesis consistent with the results rather than demonstrated by them.

Finally, because forest buffering depends on canopy structure and forest type, a brief discussion acknowledging that “forest cover proportion” does not capture forest structural variation (and that realms encompass diverse forest types and canopy properties) would strengthen interpretation and help avoid overgeneralising the buffering mechanism across biomes.

Line-specific comments
Figure 2A: If the close/intermediate/far bins are based on global percentiles, I would note that the same Tmax distance does not mean the same “closeness to the warm limit” for all species. Species with small ranges (or little climate variation within their range) may have much less room to vary in Tmax distance than wide-ranging species, so “close” is not directly comparable across species.

Lines 292–293: I suggest authors clarify how abundance/relative abundance were converted to incidence.

Lines 317–323: The manuscript appropriately notes occupancy modelling is infeasible at this scale and uses random intercepts to account for among-study differences. However, random intercepts do not correct for detection biases that covary with forest cover. A short sentence clarifying this limitation would be helpful.

(Remarks on code availability)
Code is accessible on github with a clear readme file associated with all analysis steps and figures.

Reviewer #2

(Remarks to the Author)
Thank you for the opportunity to review the manuscript titled “Temperature drives variation in avian sensitivity to forest cover”. This study is an interesting attempt to test the underlying mechanisms for the observed intraspecific variations in how populations of the same species respond to deforestation, specifically focusing on the hypothesis that populations in climates closer to the upper thermal limit of the species may be more sensitive to deforestation (below I shall simply refer to this hypothesis as pertaining to ‘thermal niche’, as a shorthand). To do this, the study compiled a large global dataset on the incidence of forest birds from three major existing databases, and assessed how climate conditions of the study sites may have mediated the relationship between species incidence and forest cover. The study found that as forest cover decreases, the incidence of a given species decreases more severely for populations in warmer macroclimates (represented by air temperature, relative to the maximum temperature in the species’ distribution range) than in cooler macroclimates, and that this pattern applied across species of different functional traits. Using microclimate data for the study sites generated from a mechanistic microclimate model, the study further found that forest cover impacted the microclimate species experience (local, under-canopy temperature, relative to the maximum air temperature in the species’ distribution range), and that species incidence exhibited a unimodal relationship with microclimate, thus concluding that (quoting abstract starting in line 9) “populations in warmer macroclimates decline in incidence in deforested landscapes because hotter microclimates push them closer to their species’ realized upper thermal tolerance limits”, and that “in contrast, populations in cooler macroclimates are less affected, or even benefit, in deforested landscapes as microclimatic temperatures are pushed closer to more optimal temperatures”.

I think the study aimed to address a very important and interesting question – including from an important angle (the position of populations in a given species’ thermal niche space) that opens up an exciting scope to link more mechanistic processes with large-scale numerical patterns. The study is also laudable in extending a great amount of effort and care in attempting to answer the question proposed, with the latter especially shown through the extensive sensitivity analyses for various methodological decisions. Overall, I very much enjoyed reading the manuscript and being taken through the journey of addressing the scientific question proposed. That said, I have a number of concerns about the study in its current form, which I hope will help the authors improve the manuscript.

1. The first concern is a conceptual mismatch between the conservation issue the study was set up to address (species’

incidence response to deforestation and more broadly, land-use change) and the operational translation of this issue (the relationship between species incidence to forest cover). Whereas the former implies an understanding of how species' incidence patterns respond to forest cover change resulting from anthropogenic modifications (i.e., relative to what natural conditions would have been), the understanding to be provided by the latter pertains to how species' incidence patterns respond to forest cover itself – i.e., as the combined outcome of natural conditions and anthropogenic modifications. The problem of this conceptual distinction is that the effect the study found of forest cover on species incidence (and its dependence on climate conditions) cannot provide really inference on possible effects of deforestation.

To illustrate this problem, imagine a hypothetical situation where a site has 70% forest cover from a baseline of 100% (i.e., 30% deforestation), whereas another site has 40% forest cover from a baseline of 40% (i.e., 0% deforestation); given the large geographical scale of this study, it is conceivable that different sites in the database could differ considerably in their baseline forest cover (i.e., in the absence of deforestation), related to different natural biophysical conditions either at the regional scale or at the more local, landscape scale. By equating the test of 'incidence~forest cover' with the test of 'incidence~deforestation', the analytical framework of this study essentially views the species incidence at the 40%-forest-cover site as representing a more deforested study system than the species incidence of the 70%-forest-cover site, when it is in fact the 'original' incidence that is free from the impacts of deforestation for its study system.

This explanation is really just a lengthy way of saying that for the species incidence patterns in the study's dataset, there is a confounding effect of baseline forest cover that is not accounted for in the current analytical framework, such that any effect found of forest cover is the combined effect of deforestation and the baseline forest cover. I am not sure how the study could make valid inference on the effect of deforestation (and the relevance of climate on this effect), other than to use matched data between deforested sites and their baseline, non-deforested sites – such that it can obtain data on the contrast in incidence and the extent of deforestation for analyses. An alternative is to simply stick to the operational question addressed by the analyses: how does species incidence respond to forest cover itself. Then it would seem to me that the study's framing should probably be revised quite a bit, including the narrative and clarification about its ecological meaning and significance (note that the 'forest cover' here also does not differentiate between native forests and plantations).

2. My second concern is regarding the extent to which the study could defensibly make inference on the 'thermal niche' hypothesis based on the finding that in the GLMM model, macroclimate was found to influence how species incidence responds to forest cover. I do not doubt that macroclimate (represented by the mean daily maximum temperature of the warmest month at each site) is a valid climate/thermal metric, but this metric could also be collinear with some other factors that can potentially affect how species responds to forest cover and that do not necessarily operate through the thermal niche angle.

One such factor I can think of is climate seasonality, which is largely correlated with temperature and probably affects the seasonality in forest foliage conditions (e.g., deciduous forests in colder climates versus evergreen forests in the tropics): regional differences in this factor can potentially mean different levels of pre-adaptation to lower/higher forest cover even in the same species (this possibility was raised for a different context by a recent paper, Hua et al. 2024 in Nature Ecology & Evolution, titled "Ecological filtering shapes the impacts of agricultural deforestation on biodiversity"). Another potential factor is disturbance history and its related 'extinction filtering' effect highlighted in the 2019 Science paper by Betts et al. and in fact noted by the Introduction of this study; in that paper, disturbance history was considered to largely follow a latitudinal gradient, which again can be highly collinear with temperature. There may be other factors that can similarly render macroecological gradients in how species respond to forest cover, in ways that are not necessarily about thermal ecology.

If the above possibility of alternative mechanisms is real (and I think it is, or at least warrants consideration), I think the study should be cautious about interpreting the statistical findings, and should at least discuss alternative interpretations of such findings. Note that in the current manuscript, the study made rather unequivocal conclusions that species response to forest cover (or 'deforestation) is mediated by distance to its upper thermal limit (as one notable example, the statement in line 221-222 says "Our results suggest that many of the impacts of changes in landscape structure on ecological communities are mediated by the effect of landscape structure on temperature").

3. My third concern is about a number of analytical decisions of the study.

3-1. Regarding the two-step analytical process of assessing the relevance of macroclimatic conditions and then microclimatic conditions: As the study noted when introducing the second step, macroclimates are hardly the actual climatic conditions a forest organism experiences. In this sense, would it really make sense to use macroclimate in the GLMM models by which the study tested the response of species incidence to forest cover and how this response may be mediated by climate? Why not go straight to using microclimatic conditions – the actual climate conditions that are ecologically relevant to particularly forest species?

3-2. Another analytical question is why the study did not choose to compile more temporally-relevant forest cover data for each primary study (presumably doable), but instead used a blanket time point of year 2000. It seems to me that not doing the latter has the added drawback of bringing in a potential confounding effect on species response to forest cover: if the surveys of different study sites were conducted in different years, their different lengths of time since 2000 could conceivably confound the incidences being compared interspecifically or intraspecifically?

3-3. An additional analytical question is about the choice of functional traits: I was struck by the fact that the list of traits considered does not include what seems to me to be a most relevant trait – i.e., the level to which a given species is dependent on (native) forest habitat. I understand that data for this trait are not readily available from existing databases, and

that is probably a barrier to the study including it, but conceptually, I think it would be a key trait to consider in the analysis here. Given the large number of species included in this study and the rather loose criteria by which these species were selected, I would imagine there to be large variations in this trait among the study species; I also think it is conceivable that this trait can influence how species responds to forest cover and how climate influences this response (note that the study concluded that traits were overall irrelevant to these effects).

3-4. Yet another analytical question is about the assessment of intraspecific variations in species response to forest cover and how this response is influenced by climate, presumably the central question of the study. From the manuscript, it seems to me that the analyses in which this question was tested were the same models used to test for interspecific variations. It is not clear to me why this is (why not more directly test for intraspecific variations), or how the interspecific models could inform on the intraspecific variations (the study mentioned derivatives/partial effects from these models, but I found the explanation in this part – in the Results section as well as the later Methods section – unclear with regard to exactly what was done and how it could provide the inference needed).

3-5. Finally and probably a nuisance question: why did the study not directly use phylogenetic regressions to account for species' phylogenetic non-independence? In its current form, the study simply "tested for phylogenetic correlation in the species-level random effects" (line 483).

4. While the writing is generally clear, I think some important methodological information is missing from the main text (even though it is provided later in Methods). Notable examples include the criteria used to select study species (line 54-58), the rationale for selecting a 600-m buffer in quantifying forest cover (line 62; note that the explanation provided in the Methods section on this point – in line 338-341 – is also not very clear), and the model structure used to assess the effects of predictor variables (the first paragraph of the Results and discussion section).

It also seems to me that for a number of important methodological decisions, the manuscript did not explain how they were made or done. Examples include the time stamp of the databases consulted when compiling the dataset used in the study, how the random intercepts were structured that accounted for "variations in sampling methods, detection probabilities and sampling efforts among studies" (line 321-323), and the rationale for choosing the list of traits considered – either in the main text or Methods sections.

(Remarks on code availability)

Version 1:

Reviewer comments:

Reviewer #1

(Remarks to the Author)
I thank the authors for their careful and thorough responses to my previous comments. I have reviewed the revised manuscript and response letter, and I am satisfied that my concerns have been addressed.

In particular, the authors have clarified the interpretation of Tmax as a realised macroclimatic warm limit rather than a physiological threshold, added appropriate caveats regarding habitat-specific exposure and thermally buffered microhabitats, incorporated range size and primary habitat affiliation into the trait analyses, provided additional detail on the microclimate validation datasets, and added useful analyses addressing concerns related to interannual variation in BIO05, biome-level patterns, latitude, and among-species variation in responses.

I have a minor comment regarding the new analyses whereby 52% of species showed the predicted within-species pattern (populations closer to the species' warm limits exhibit greater sensitivity to forest cover). Simply, this is only just over half, and I encourage the authors to ensure that broad claims about intraspecific responses are framed as average tendencies rather than general rules. More specifically, the wording in the abstract, fig 2 caption (eg. The location of a population relative to its species' realised warm tolerance limit (Tmax distance) mediates both inter- and intraspecific variation in how forest cover affects bird incidence), as well as the section heading for the corresponding results (eg. Thermal exposure explains inter- and intraspecific variation) could be toned down a bit more to emphasise that Tmax distance explains an important component of variation, while substantial species-level heterogeneity remains.

Other than the above, I have no further substantive concerns and would support publication.

(Remarks on code availability)

Reviewer #2

(Remarks to the Author)
Thank you to the authors for their thoughtful revision of the manuscript. I think overall, this revision has successfully addressed the previous concerns, and is shaping up to be a strong contribution to the journal. I have the following, mostly

small, comments for the authors to consider.

1. Regarding the current framing of the study as a direct test of species response to forest cover that can inform the bigger question of species response to deforestation: I think this is a sensible way to frame the study, and the authors did a good job making the case for the linkage between what the study did exactly and its 'bigger-question' interpretations in their response letter. But I think the entry of the framing in the manuscript should be improved a bit more.

Currently, the specific topic of 'bird response to forest cover' enters abruptly right after the manuscript opens very broadly with land use change, without any transition to link it with the preceding content meant essentially to set the scene for deforestation. The argument needed for this linkage enters only later in the next paragraph (lines 66-68) – after **the** paragraph on what the study did and why it is relevant to the broader question. I think when the manuscript brings up the study aim, which is about specie response to forest cover, it should explain and clarify right there the rationale of this aim – why it is a fitting way to respond to the broader issue and its gaps. In its current form, when the study enters the picture, there remains a disconnect between what the study did and what it is apparently set up to do.

2. Regarding the key finding of negative relationships between species incidence and forest cover in macroclimates further from species' warm limits (lines 116-117), one question the reader would naturally have is how so. Do you have any ecological interpretations for this counterintuitive finding, or could it rather be a statistical artefact? I think this counterintuitive finding should probably be given some discussion.

3. For the analysis on traits, the study essentially used multiple single regressions on individual traits. But how reasonable is this, given that if multiple traits are relevant to species response, then assessing one trait at a time will inevitably generate biased estimates of the effect of individual traits? (This question pertains to the general rationale for running multiple regressions rather than multiple single regressions.)

4. Regarding the presentation of methods information, I think this version is much improved. But there remain some small issues:
a. The sentence in lines 129-133 needs improved clarity in terms of its meaning.
b. For the analysis on functional traits, the sentence in lines 187-188 noted the traits that are not (strongly) relevant as 'morphological or functional traits'. Given the study's findings, this term should not include range size and species' dependence on forest habitat (these two traits were indeed found to be relevant), which can be at odds with the typical interpretation of this term. I think rephrasing of this statement is probably warranted.
c. Does the word 'derivative' when discussing model interpretation and results mean slope (coefficient of predictor variables; e.g. as used in line 629)? If so, slope / coefficient is a much more intuitive and familiar term than 'derivative'.

5. Some small writing issues:
a. Grammar of the sentence in lines 175-176 needs checking.
b. The word 'deforestation' in line 265 should be changed to 'forest cover'.
c. The sentence in lines 634-635 should be checked for sentence completion and clarity.

(Remarks on code availability)

Open Access This Peer Review File is licensed under a Creative Commons Attribution 4.0 International License, which permits use, sharing, adaptation, distribution and reproduction in any medium or format, as long as you give appropriate credit to the original author(s) and the source, provide a link to the Creative Commons license, and indicate if changes were made.
In cases where reviewers are anonymous, credit should be given to 'Anonymous Referee' and the source.
The images or other third party material in this Peer Review File are included in the article's Creative Commons license, unless indicated otherwise in a credit line to the material. If material is not included in the article's Creative Commons license and your intended use is not permitted by statutory regulation or exceeds the permitted use, you will need to obtain permission directly from the copyright holder.
To view a copy of this license, visit https://creativecommons.org/licenses/by/4.0/

Dear Dr Andriuzzi,

We would like to thank you and the reviewers for the time and care taken to review our manuscript. We are grateful for the insightful suggestions which have substantially improved the clarity, rigour and scope of the study. We have addressed all the comments in full and paid particular attention to the following key points:

(1) **Accounting for latitude as a potential correlate of T_{max} *distance*:** We have added an additional analysis that explicitly controls for latitude as a potential confounding factor. Our results were robust to accounting for latitudinal effects on sensitivity to forest cover. Indeed, while latitude was significant in isolation, latitude was no longer a significant predictor of response to forest cover having accounted for T_{max} *distance.*

(2) **Inter- vs. intraspecific variation**: We have restructured the text in the Methods and Results to more clearly explain how the effect of T_{max} *distance* on within-species variation in responses to forest cover was disentangled from the overall variation across all species. In particular, we conducted a new analysis quantifying the proportion of species (52%) for which populations closer to their species' warm limits exhibit greater sensitivity to forest cover.

(3) Additional traits: forest specialisation and range size: Following the reviewers' suggestions, we incorporated two additional traits into our trait-based analyses. We found that species with larger geographic ranges, and species for which forest is the primary habitat affiliation, were more likely to show heightened sensitivity to forest cover near their warm limits.

(4) **Expanded justification of methodological decisions**: We have strengthened the rationale for key analytical choices and clarified caveats throughout the manuscript, in accordance with the reviewers' suggestions. We also conducted an additional sensitivity analysis which showed that accounting for phylogenetic relatedness does not affect our results.

(5) **Clarifying *forest cover* and *deforestation***: We now consistently use the term *forest cover* when describing our Methods and Results. As clarified in the manuscript and detailed in our response to reviewer 2, the design and location of the studies within our dataset means that our results can provide insights into the likely impacts of deforestation, which we link to in the Introduction and Discussion.

(6) **Biome-specific analyses:** In addition to analysing variation in the temperature-mediation of forest cover responses across biogeographic realms, we have also

analysed variations across biomes, following the suggestion from reviewer 1. We found broadly consistent patterns across biomes.

Detailed point-by-point responses to all reviewer comments are provided below. Our responses are written in blue text beneath each comment, and line numbers refer to the marked-up version of the revised manuscript.

Reviewer #1:

The authors have produced an ambitious and thoughtfully constructed manuscript that I enjoyed reading. The integration of macroclimatic thermal position, fine-scale microclimatic buffering, and global bird community data to explain variation in avian responses to forest cover is conceptually elegant. The central idea, whereby proximity to a species' realised warm limit mediates both inter- and intraspecific sensitivity to forest cover, is clearly supported by the analyses. Overall, I congratulate the authors on an exciting and well written manuscript and consider this work a strong fit for Nature Ecology & Evolution.

Thank you so much for taking the time to review our manuscript. We were happy to read these positive remarks, and we are very grateful for the valuable insights you have shared.

Major comments

The manuscript defines each species' warm limit (Tmax) as the highest value of BIO05 (mean daily maximum temperature of the warmest month) experienced anywhere within the species' BirdLife range polygon, using ERA5 in 2000. This is appropriately framed as a realised warm tolerance inferred from distribution rather than a physiological limit. However, because Tmax is calculated across entire range polygons using coarse macroclimate grids, it is best interpreted as the upper envelope of regional macroclimatic conditions associated with the mapped range, rather than temperatures necessarily experienced in the forest microhabitats where many populations occur. The authors do acknowledge this issue directly (i.e., that Tmax distance based on site macroclimate may not represent temperatures birds experience inside forests) and the microclimate modelling is done on this basis. It would be helpful to briefly discuss that some species may not occupy the hottest parts of their mapped range (or may persist there only in buffered microhabitats). The Appendix 7 sensitivity test (95th percentile Tmax) addresses potential outliers within ranges, but not habitat-specific exposure; acknowledging this would improve interpretability.

This is a good point – thank you for raising it. We agree with your description of T_{max} and have now clarified this accordingly in the manuscript and have highlighted the possibility that species may not occupy the hottest parts of their mapped range, or may persist there only in buffered microhabitats. In particular, we have made the following changes:

1) We have clarified the definition of T_{max} in the introduction. The revised text now reads: *"This represents the upper envelope of regional macroclimatic conditions associated with the mapped range, which provides an estimate of each species' realised warm tolerance limit*[1]*, hereafter referred to as the species' "warm limit" and abbreviated as* T_{max}*"* (lines 84-87).
2) We have added further clarification to lines 462-470 in the Methods: *"we inferred species' realised warm limits from their geographic range maps, which provided a standardised approach that could be generalised across the 2,262 species in our study. These warm limits represent the upper envelope of regional macroclimatic conditions associated with the mapped range. To test the robustness of our results against potential temperature outliers within species' ranges, or potential use of cooler microhabitats by species within the hottest pixel of their geographic range, we also calculated the warm limits using the 95th percentile of the hottest monthly temperature within each species range, rather than the maximum and found that this did not affect our results (Appendix 8).*
3) We have expanded on the justification for the sensitivity test in appendix 8 (95th percentile T_{max}) by acknowledging the possibility that species might dwell only in thermally buffered microclimates when located in the hottest part of their range. For instance, lines 267-271 of the Supplementary Material now read: *"There is also a possibility that organisms could try to avoid exposure to the hottest macroclimatic temperatures within their geographic ranges, for instance by adjusting their distribution within their range and dispersing to cooler parts of their range, or by dwelling only within thermally buffered microhabitats when located within the hottest part of their range*[2,3]*."*

Please clarify how the Tmax-distance interpretation applies to species whose ranges span relatively few ERA5 grid cells or limited climatic variation. In such cases, Tmax may reflect restricted climatic breadth. The manuscript currently emphasises generality across traits, but range breadth is an important separate axis.

Thank you for raising this important point. We agree that for species whose ranges span relatively few ERA5 grid cells or a limited range of climatic conditions, T_{max} *distance* may largely reflect restricted climatic breadth. For such species, populations are likely to be consistently close to their warm limit, and therefore sensitivity to forest cover may be high across most or all of the species' range. In contrast, species with larger geographic and climatic ranges likely experience greater variation in thermal environments, which we expect would allow for more variation in responses to forest cover.

We have now explicitly incorporated species' geographic range size as an additional trait in our analyses. As expected, species with larger ranges were more likely to follow the overall trend of more positive responses to increasing forest cover near their warm limit and more negative responses to increasing forest cover far from their warm limit. These results are shown in Extended Data Figure 4A and Table S7 and discussed on lines 171-176, which are as follows: *"Species' geographic range size was the strongest predictor of variation in how* T_{max} *distance affects responses to forest cover (Extended Data Figure 4; Table S7). Species with larger ranges were more likely to show more positive effects of forest cover on incidence close to* T_{max}*, and more negative effects of forest cover on incidence further from* $T_{max.}$ *Whereas species with small ranges are likely to always be located close to their* $T_{max.}$*, so are likely to be highly sensitive to forest cover throughout their range."*

The microclimate component is central to the mechanistic interpretation, and the results explicitly rely on it to argue that forest cover alters microclimatic thermal exposure and thus mediates incidence responses. I have two suggestions here:
1) The model is validated against four logger datasets. However, I suggest that the authors provide a bit more detail on logger placement (height above ground and forest cover/type) for each validation dataset. This is important because the simulations are conducted at 2 m above ground, and "above-ground in situ" could still encompass a range of placements that affect comparability.

Thanks for the suggestion. We have now provided more detail in the Methods on logger placement, including height above ground and forest type. To clarify, for the validation, we modelled microclimatic temperatures at the same height above ground as the corresponding loggers in each dataset, to enable comparison between observed and modelled values. Loggers were placed at 1 m above ground in the Atlantic Forest and Borneo datasets, and at 1.5 m above ground in the Oregon datasets, and modelled temperatures were generated at

these same heights. For the main analyses presented in the manuscript, we modelled microclimatic temperatures at 2 m above ground to enable comparison between modelled microclimates and species' warm limits (which were from macroclimatic data at 2 m above ground) when we calculated *micro*T_{max} *distance*.

We have clarified this explicitly on lines 568-571*: "For each site, we applied the modelling method described above but modelled the microclimate at the same height above ground at which the loggers were placed for each dataset (1 m for the Atlantic Forest and Borneo datasets, and 1.5 m for the Oregon dataset)."*

2) Because mechanistic microclimate modelling is a major element of the analyses, I recommend introducing it more explicitly in the introduction (even briefly). As written, the reader could form an early impression that the study is purely macroclimate-driven before encountering the valuable mechanistic component.

Thank you for this recommendation. We have now introduced the mechanistic microclimate component more explicitly when introducing the purpose of our study at the start of the third paragraph of the Introduction. This section now reads: *"We first analyse how macroclimate temperature affects responses to forest cover within and across species. Then, we use mechanistic microclimate modelling to propose an explanation for this macroecological variation"* (lines 50-53).

The manuscript justifies using the year 2000 to align with the forest cover data and argues that relative forest/non-forest temperature differences are not expected to change, even if absolute temperatures differ in later survey years. However, because BIO05 captures peak seasonal heat (mean daily maximum in the warmest month), it may be more sensitive to interannual variability than broader metrics like mean annual temperature. I suggest adding a brief statement on whether 2000 was checked against a longer-term climate for BIO05. This point is particularly relevant because the interpretation hinges on "distance to warm limits," which could shift with year-to-year extremes.

This is a good point – thank you for raising it. We have checked the BIO05 temperature in 2000 against the long-term climate average (1979-2018) for the sites in our study and found that these two variables were very closely related (correlation coefficient = 0.97, R^2 = 0.93,

RMSE = 1.16), indicating that the BIO05 temperature in 2000 is a good approximation of the longer-term average at our sites (Appendix 9).

Appendix 9 includes a description of how this comparison was carried out: "*Since the maximum monthly temperature (BIO05) may show inter-annual variations, we tested how closely the BIO05 temperature approximates the longer-term 40-year climate average of this variable. We downloaded the average BIO05 temperature between 1979-2018 at 0.5° resolution from the ERA5 Bioclimatic Indicators dataset*[4]*, and compared this with the BIO05 temperature in 2000 at 0.5° resolution also from the ERA5 Bioclimatic Indicators dataset. We extracted the values of both variables for our study sites and found that the two variables were highly correlated (correlation coefficient = 0.97, Figure S17), indicating that the BIO05 temperature in the year 2000 is a close approximation of the longer-term average.*" (lines 322-330 in Supplementary Material).

The comparison is also illustrated in Figure S17 and stated in the Methods on lines 449-451 which read: *"The BIO05 temperature in the year 2000 at our study sites was closely related to the longer-term 40-year average BIO05 temperature at these sites (Appendix 9)."*

Figure 2C shows substantial interspecific variation in the species-specific effects of forest cover across Tmax distance, with many species exhibiting slopes opposite to the overall mean trend. The manuscript does acknowledge that results identify general trends but do not reveal underlying processes. It would be useful to explicitly quantify this heterogeneity (e.g., proportion of species with positive vs negative forest sensitivity at low vs high Tmax distance; or distribution of species-level interaction terms). This would clarify whether the key inference is best viewed as a shift in the average response across species/populations rather than a rule that applies to most species.

Thank you for this helpful suggestion. We have now explicitly quantified this heterogeneity. For each species, we estimated the direction of the relationship between T_{max} *distance* and sensitivity to forest cover by fitting a linear regression to the species-specific derivatives of incidence with respect to forest cover across T_{max} *distance* (i.e. the lines shown in Figure 2C).

We found that 52% of species exhibited slopes consistent with the average overall trend of more positive responses to increasing forest cover close to T_{max}, as discussed on lines 138-146, which are as follows: "*Out of the 2,262 species in our study, 2,143 could be used to*

assess how T_{max} distance modulates within-species responses to forest cover, as these species exhibited some variation in T_{max} distance either within or across studies. Of these 2,143 species, 1,114 (52%) showed increasingly positive effects of forest cover on incidence when located closer to T_{max} (Extended Data Figure 3). We also calculated the partial effect of forest cover for the fixed-effects component of the model. This showed an overall mean trend in which increasing forest cover had a positive effect on incidence at temperatures close to Tmax, and a shift in the average response to a negative effect of increasing forest cover on incidence at temperatures > 15.8 °C cooler than Tmax, when all species are analysed together (Figure 2C).”

We have described on lines 637-647 in the Methods how this quantification was carried out: *“We visualised both the overall fixed effect derivatives and the species-specific random effect derivatives to characterise how the overall average response to forest cover varies with T_{max} distance, and to illustrate heterogeneity in these patterns among species (Figure 2C, Extended Data Figures 3 & 4). To summarise the direction and relative magnitude of the association between T_{max} distance and the response to forest cover within each species, we fitted a separate linear regression for each species, with T_{max} distance as the predictor and the derivative with respect to forest cover as the response. These regressions were used solely as descriptive summaries of the direction and relative magnitude of species-specific trends. Formal statistical inference was not assessed because T_{max} distance served both as the predictor in these regressions and as an input to the derivative calculations, resulting in no residual variance around the fitted relationships.”*

The manuscript reports that the Tmax distance × forest cover interaction varies among biogeographic realms, being weakest in Australasia, and some realms have notably fewer sites (e.g., Australasia, Indo-Malaya). This makes it difficult to distinguish genuine biological differences from reduced statistical power and uneven sampling. In addition, realms differ substantially in internal climatic and habitat heterogeneity (e.g., Afrotropical encompasses Mediterranean, montane, and tropical systems), which may influence both the distribution of Tmax distances and the strength of forest buffering. Please clarify these limitations and consider summarising how Tmax distance (and its variance) is distributed within and among realms, to support interpretation of realm-level differences.

Thank you for raising these important points. To address some of the climatic and habitat heterogeneity within realms, we have now included additional analyses which test for differences across biomes. These analyses showed that the temperature-mediation of forest cover responses and the microclimate buffering effect of forests were relatively consistent across biomes (Extended Data Figure 2; Table S4; Extended Data Figure 5; Table S16).

To address the concern regarding uneven sampling effort and differences in the distribution of T_{max} *distance*, we have added a new figure (Extended Data Figure 1) illustrating the distribution and variance of T_{max} *distance* within and among both realms and biomes. We also annotate the sample sizes directly on Figure 2B and Extended Data Figure 2. We have also revised the manuscript text to explicitly acknowledge these limitations when interpreting differences among realms and biomes: *"We found this same pattern across all biomes and realms, though the interaction between* T_{max} *distance and forest cover was strongest in the Nearctic and Neotropical realms and weakest in the Australasian realm (Extended Data Figure 2, Figure 2B, Tables S3 & S4). However, we note that sample sizes and spans of* T_{max} *distance differed among realms and among biomes (Extended Data Figure 1)"* (lines 120-125).

The manuscript links results to a latitudinal narrative (low latitudes being closer to warm limits). While plausible, latitude is not directly analysed, and realms contain substantial internal heterogeneity. I recommend either (i) adding an explicit latitude/biome analysis, or (ii) softening the wording so that the latitudinal mechanism is framed more clearly as a hypothesis consistent with the results rather than demonstrated by them.

Thank you for the suggestion. We have added an explicit latitude analysis and a biome analysis.

In response to this comment and the comment above, we analysed how T_{max} *distance* affects responses to forest cover within each biome (Extended Data Figure 2 and Table S4), We found similar patterns across all six biomes analysed, with heightened sensitivity to forest cover close to T_{max}, though there were large differences in sample size across biomes (as discussed in the response to the comment in above).

The role of latitude was also raised by reviewer 2 (main comment #2). We have added an additional analysis where we accounted for the effect of latitude on response to forest cover in our main model which tests how T_{max} *distance*, forest cover and their interaction

affect incidence (Appendix 4). We found that the effect of T_{max} *distance* on response to forest cover was unaffected by accounting for latitude. As shown in Appendix 4, and discussed on lines 288-295: "*the effect of T_{max} distance on response to forest cover remained the same even when we included latitude as an additional predictor in our model. While latitude was significant in isolation, latitude was no longer a significant predictor of response to forest cover having accounted for T_{max} distance (Figure S2; Table S20, Appendix 4). While further studies should investigate the relative contributions of different drivers of the inter- and intraspecific variation in responses to forest cover, these results suggest that proximity to species' warm limits may be an important driver of latitudinal variation in responses to forest cover.*

Nonetheless, while it is indeed plausible that populations at low latitudes are closer to their species' warm limits, latitude and T_{max} *distance* are moderately correlated (correlation coefficient = 0.46, Appendix 4), implying there is some heterogeneity in this relationship. Therefore, when linking our results with those of Deutsch et al 2008, we have softened the wording which suggested that populations in our study which are close to their warm limits would necessarily be at low latitudes.

The revised section now reads: *"The impact of proximity to species' warm limits on terrestrial endotherm responses to changes in forest cover, such as through land-use change, aligns with global efforts to understand terrestrial ectotherm responses to climatic warming* [5]*. Deutsch et al (2008) showed that populations located at low latitudes are likely to be most negatively affected by warming as they are currently located in environments near their warm limits. By contrast, populations at higher latitudes may be positively affected as they are currently located in environments cooler than their warm limits, and potentially cooler than their optimal temperatures* [5]*. This suggests that proximity to species' warm limits mediates variation in how they respond to two major global change drivers"* (lines 296-304).

Finally, because forest buffering depends on canopy structure and forest type, a brief discussion acknowledging that "forest cover proportion" does not capture forest structural variation (and that realms encompass diverse forest types and canopy properties) would strengthen interpretation and help avoid overgeneralising the buffering mechanism across biomes.

Thanks for picking up on this. We have now added an analysis that tests explicitly the thermal

buffering effect of forests within each biome (Extended Data Figure 5). Though the sample sizes differ among biomes, as discussed above, we found that forests buffer hot microclimate temperatures across all biomes.

While the microclimate estimates do incorporate information on canopy height and leaf-area index, which we have made clearer on line 214, you are right to raise the point that the forest cover proportion variable used as a predictor in our statistical models does not capture forest structural variation. We have now acknowledged this on lines 436-439: *"Using this global tree cover dataset provides a valuable opportunity to estimate forest cover worldwide using a standardised approach, thereby enabling global analyses of bird responses to forest cover. However, we note that this measure does not capture variation in forest structure and does not distinguish between native forests and plantation forests."*

Line-specific comments

Figure 2A: If the close/intermediate/far bins are based on global percentiles, I would note that the same Tmax distance does not mean the same "closeness to the warm limit" for all species. Species with small ranges (or little climate variation within their range) may have much less room to vary in Tmax distance than wide-ranging species, so "close" is not directly comparable across species.

Thanks for the suggestion. We deliberately quantified *T_{max} distance* as the raw, unscaled difference in temperature between the warm limit and the site temperature to enable such comparability across species. Using this method, the same *T_{max} distance* does indeed mean the same closeness to the warm limit for all species. For instance, a *T_{max} distance* of 5 (red line in Figure 2A) means the site is 5 °C cooler than the species' warm limit, and this interpretation is the same across all species. A *T_{max} distance* of 20 (blue line in Figure 2A) means the site is 20 °C cooler than the species' warm limit; species with small ranges would not be included in this bin, if they cannot be 20 °C from their warm limit.

For clarity, we have written in the legend the *T_{max} distance* values represented by the global percentiles in Figure 2A (5 °C, 12 °C and 20 °C from the warm limit). Furthermore, we have added annotations to Extended Data Figure 1 (histograms showing distribution of *T_{max} distance* within each realm and biome) which show the *T_{max} distance* values that correspond to these percentiles which are plotted in Figure 2B (realms) and Extended Data Figure 2 (biomes).

Lines 292–293: I suggest authors clarify how abundance/relative abundance were converted to incidence.

We have clarified this on lines 376-379, which are as follows: *"... the Atlantic Birds database reports bird species' relative abundances (i.e., the number of individuals recorded per unit of sampling time) within studies across the Brazilian Atlantic Forest* [8]*. We converted this to incidence for each study; if a species was found at one site within a study, but not at another site within that same study, then we recorded that species as absent at the latter site."*

Lines 317–323: The manuscript appropriately notes occupancy modelling is infeasible at this scale and uses random intercepts to account for among-study differences. However, random intercepts do not correct for detection biases that covary with forest cover. A short sentence clarifying this limitation would be helpful.

Thanks for picking up on this. We have now clarified this in the Methods: *"With this approach, it is not possible to adjust for imperfect detection in the underlying studies using methods such as occupancy models due to the large number of species in the dataset* [9,10]*. We therefore used random intercepts in statistical models to account for variation among studies. However, this approach does not correct for detection biases that may covary with forest cover"* (lines 409-412).

Reviewer #1 (Remarks on code availability):
Code is accessible on github with a clear readme file associated with all analysis steps and figures.

Reviewer #2:

Thank you for the opportunity to review the manuscript titled "Temperature drives variation in avian sensitivity to forest cover". This study is an interesting attempt to test the underlying mechanisms for the observed intraspecific variations in how populations of the same species respond to deforestation, specifically focusing on the hypothesis that populations in climates closer to the upper thermal limit of the species may be more sensitive to deforestation (below I shall simply refer to this hypothesis as pertaining to 'thermal niche', as a shorthand). To do this, the study compiled a large global dataset on the incidence of forest birds from three major existing databases, and assessed how climate conditions of the study sites may have mediated the relationship between species incidence and forest cover. The study found that as forest cover decreases, the incidence of a given species decreases more severely for populations in warmer macroclimates (represented by air temperature, relative to the maximum temperature in the species' distribution range) than in cooler macroclimates, and that this pattern applied across species of different functional traits. Using microclimate data for the study sites generated from a mechanistic microclimate model, the study further found that forest cover impacted the microclimate species experience (local, under-canopy temperature, relative to the maximum air temperature in the species' distribution range), and that species incidence exhibited a unimodal relationship with microclimate, thus concluding that (quoting abstract starting in line 9) "populations in warmer macroclimates decline in incidence in deforested landscapes because hotter microclimates push them closer to their species' realized upper thermal tolerance limits", and that "in contrast, populations in cooler macroclimates are less affected, or even benefit, in deforested landscapes as microclimatic temperatures are pushed closer to more optimal temperatures".

This is an accurate and thorough summary of our study. Thank you so much for taking such time and care in reading and reviewing it. We really appreciate the insights you have shared.

I think the study aimed to address a very important and interesting question – including from an important angle (the position of populations in a given species' thermal niche space) that opens up an exciting scope to link more mechanistic processes with large-scale numerical patterns. The study is also laudable in extending a great amount of effort and care in attempting to answer the question proposed, with the latter especially shown through the extensive sensitivity analyses for various methodological decisions. Overall, I very much enjoyed reading the manuscript and being taken through the journey of addressing the

scientific question proposed. That said, I have a number of concerns about the study in its current form, which I hope will help the authors improve the manuscript.

Thank you for these positive and encouraging comments. We are really pleased to hear that you enjoyed reading the manuscript, and we agree that the study opens up an exciting scope to link more mechanistic processes with large-scale patterns. We have addressed all your concerns in the revised version of the manuscript, and in our point-by-point responses below. We agree that these changes have improved the manuscript and we are very grateful for your help.

1. The first concern is a conceptual mismatch between the conservation issue the study was set up to address (species' incidence response to deforestation and more broadly, land-use change) and the operational translation of this issue (the relationship between species incidence to forest cover). Whereas the former implies an understanding of how species' incidence patterns respond to forest cover change resulting from anthropogenic modifications (i.e., relative to what natural conditions would have been), the understanding to be provided by the latter pertains to how species' incidence patterns respond to forest cover itself – i.e., as the combined outcome of natural conditions and anthropogenic modifications. The problem of this conceptual distinction is that the effect the study found of forest cover on species incidence (and its dependence on climate conditions) cannot provide really inference on possible effects of deforestation.

Thank you for raising this important point. Due to three key aspects of our study design, we believe our analyses can provide inference on possible effects of deforestation: (1) we filtered our dataset to only include studies conducted in forest ecoregions; (2) the studies within our dataset were specifically designed to understand the effects of deforestation, and (3) the studies span comprehensive gradients of forest cover, as shown in Figure 2C. We have highlighted more clearly on lines 58-60 of the Introduction that the studies were conducted in forest ecoregions and were specifically designed to investigate the effects of deforestation. These lines are as follows: *"The data within these databases come from studies designed to understand the responses of ecological communities to land-use change. We restricted our analysis to studies conducted within forest ecoregions* [11] *and species for which forest was listed as one of their habitat affiliations according to the IUCN habitat classification* [12]*."*

To illustrate this problem, imagine a hypothetical situation where a site has 70% forest cover from a baseline of 100% (i.e., 30% deforestation), whereas another site has 40% forest cover from a baseline of 40% (i.e., 0% deforestation); given the large geographical scale of this study, it is conceivable that different sites in the database could differ considerably in their baseline forest cover (i.e., in the absence of deforestation), related to different natural biophysical conditions either at the regional scale or at the more local, landscape scale. By equating the test of 'incidence~forest cover' with the test of 'incidence~deforestation', the analytical framework of this study essentially views the species incidence at the 40%-forest-cover site as representing a more deforested study system than the species incidence of the 70%-forest-cover site, when it is in fact the 'original' incidence that is free from the impacts of deforestation for its study system.

Thank you for the clear and illustrative example. In our dataset, we aimed to minimise this issue through study selection. We restricted analyses to studies conducted within forest ecoregions, which are likely to have been almost entirely forested prior to any anthropogenic disturbance. We deliberately excluded natural non-forest systems, e.g. savannahs, where the baseline forest cover is low. Therefore, we consider it unlikely within our dataset that 40% forest cover would represent a baseline condition. Variation in forest cover within our dataset is likely to reflect anthropogenic forest loss rather than natural baseline differences.

This explanation is really just a lengthy way of saying that for the species incidence patterns in the study's dataset, there is a confounding effect of baseline forest cover that is not accounted for in the current analytical framework, such that any effect found of forest cover is the combined effect of deforestation and the baseline forest cover. I am not sure how the study could make valid inference on the effect of deforestation (and the relevance of climate on this effect), other than to use matched data between deforested sites and their baseline, non-deforested sites – such that it can obtain data on the contrast in incidence and the extent of deforestation for analyses. An alternative is to simply stick to the operational question addressed by the analyses: how does species incidence respond to forest cover itself. Then it would seem to me that the study's framing should probably be revised quite a bit, including the narrative and clarification about its ecological meaning and significance (note that the 'forest cover' here also does not differentiate between native forests and plantations).

We have carefully checked the text, and revised it where needed, to ensure that we use the term "forest cover" consistently when referring to our methods and results. We also note that

the manuscript title uses the term forest cover. For the reasons detailed above, we are confident that from this analysis, we can make inferences on the likely impacts of deforestation.

We have set up the rationale for this inference in the Introduction on lines 66-68, which read: *"Our global analysis employs a space-for-time substitution, comparing bird incidence probabilities across sites spanning gradients in forest cover, located within forest ecoregions, to infer how populations may respond to deforestation"***.**

Then, we consistently use the term forest cover throughout the manuscript when referring to our methods and results. In particular, we have revised the legend for Figure 3 and the paragraph on lines 261-272 to align with this narrative by referring to *changes associated with forest cover* rather than *effects of deforestation*.

Then, in the Discussion on lines 273-276, when discussing potential implications of our results, we include the following rationale for the potential inferences about deforestation: "*By analysing bird responses in landscapes of varying forest cover proportions in forest ecoregions which are assumed to have been almost entirely forested prior to anthropogenic disturbance, our study can provide insight into the impacts of deforestation on bird assemblages, and how these impacts vary with temperature."*

We have also highlighted the limitations of space-for-time studies and emphasised the need for further studies using temporal data to look directly at deforestation over time: *"The use of a space-for-time substitution enabled us to investigate global variation in responses across a wide, comprehensive gradient of forest cover. However, we emphasise the need for future studies that use temporal data to directly analyse the temperature-mediation of how deforestation affects bird assemblages."* (lines 276-280)

Thank you also for noting that the forest cover variable used here does not differentiate between native forests and plantations. We have now clarified this on lines 436-439: *"Using this global tree cover dataset provides a valuable opportunity to estimate forest cover worldwide using a standardised approach, thereby enabling global analyses of bird responses to forest cover. However, we note that this measure does not capture variation in forest structure and does not distinguish between native forests and plantation forests."*

2. My second concern is regarding the extent to which the study could defensibly make inference on the 'thermal niche' hypothesis based on the finding that in the GLMM model, macroclimate was found to influence how species incidence responds to forest cover. I do not doubt that macroclimate (represented by the mean daily maximum temperature of the warmest month at each site) is a valid climate/thermal metric, but this metric could also be collinear with some other factors that can potentially affect how species responds to forest cover and that do not necessarily operate through the thermal niche angle.

One such factor I can think of is climate seasonality, which is largely correlated with temperature and probably affects the seasonality in forest foliage conditions (e.g., deciduous forests in colder climates versus evergreen forests in the tropics): regional differences in this factor can potentially mean different levels of pre-adaptation to lower/higher forest cover even in the same species (this possibility was raised for a different context by a recent paper, Hua et al. 2024 in Nature Ecology & Evolution, titled "Ecological filtering shapes the impacts of agricultural deforestation on biodiversity").

Another potential factor is disturbance history and its related 'extinction filtering' effect highlighted in the 2019 Science paper by Betts et al. and in fact noted by the Introduction of this study; in that paper, disturbance history was considered to largely follow a latitudinal gradient, which again can be highly collinear with temperature. There may be other factors that can similarly render macroecological gradients in how species respond to forest cover, in ways that are not necessarily about thermal ecology. If the above possibility of alternative mechanisms is real (and I think it is, or at least warrants consideration), I think the study should be cautious about interpreting the statistical findings, and should at least discuss alternative interpretations of such findings.

Thank you for raising this insightful point. We agree that it is important to consider potential confounding factors. We have now incorporated these alternative explanations into the Discussion. For instance, lines 284-286: *"It is important to consider that this thermal effect could potentially be confounded because gradients in temperature covary across latitude along with other environmental (e.g. seasonality), ecological (e.g. dispersal ability) and historical (e.g. disturbance) factors that have previously been linked to responses to forest cover* [13, 14, 15]*."*

In addition, we incorporated latitude into our main GLMM to account for its potential confounding effect on responses to forest cover. We found that our results were robust to

accounting for latitude. When including both latitude and T_{max} *distance* together in this model, T_{max} *distance* moderated responses to forest cover more strongly than latitude. This is explained on lines 288-295*: "the effect of T_{max} distance on response to forest cover remained the same even when we included latitude as an additional predictor in our model. While latitude was significant in isolation, latitude was no longer a significant predictor of response to forest cover having accounted for T_{max} distance (Figure S2; Table S20, Appendix 4). While further studies should investigate the relative contributions of different drivers of the inter- and intraspecific variation in responses to forest cover, these results suggest that proximity to species' warm limits may be an important driver of latitudinal variation in responses to forest cover."*

These results are shown in Appendix 4, and we have included the following explanation of this additional analysis in Appendix 4: *"T_{max} distance is correlated with absolute latitude (correlation coefficient = 0.47), and latitude has been shown to mediate responses to forest cover, with species at lower latitudes showing heightened sensitivity to forest cover* [13–15]*. Therefore, we tested whether accounting for the potentially confounding effect of latitude changes the effect of T_{max} distance on responses to forest cover. We found that the effect of T_{max} distance on responses to forest cover was robust to accounting for latitude.*

We found that latitude mediated responses to forest cover (Table S18; Figure S1), consistent with previous studies [13–15]*, but latitude did not have a significant effect when it was included in a model with T_{max} distance (Table S19; Figure S2). The effect of T_{max} distance on response to forest cover, as reported in the main text (e.g. Table S2; Figure 2A) remained the same even when accounting for the effect of latitude on response to forest cover (Table S19; Figure S2)."* (lines 76-89 of Supplementary Material).

Note that in the current manuscript, the study made rather unequivocal conclusions that species response to forest cover (or 'deforestation) is mediated by distance to its upper thermal limit (as one notable example, the statement in line 221-222 says "Our results suggest that many of the impacts of changes in landscape structure on ecological communities are mediated by the effect of landscape structure on temperature").

Thank you for noting this. Our results provide evidence that species responses to forest cover are mediated by distance to their upper thermal limits (e.g. Figure 2A; Figure S2). To avoid unequivocal phrasing, we have revised the sentence in question. This sentence now reads: *"Our results have shown that maximum temperature is an important mediator of variation in*

responses to forest cover, which can be explained by local microclimatic changes." (lines 282-284).

3. My third concern is about a number of analytical decisions of the study.

3-1. Regarding the two-step analytical process of assessing the relevance of macroclimatic conditions and then microclimatic conditions: As the study noted when introducing the second step, macroclimates are hardly the actual climatic conditions a forest organism experiences. In this sense, would it really make sense to use macroclimate in the GLMM models by which the study tested the response of species incidence to forest cover and how this response may be mediated by climate? Why not go straight to using microclimatic conditions – the actual climate conditions that are ecologically relevant to particularly forest species?

This is a good question – thank you for asking it. Our two-step analytical approach reflects both statistical constraints and conceptual aims. We did not use microclimate in the GLMMs in the first step of analysis (i.e. we used incidence ~ forest cover * T_{max} *distance* and not incidence ~ forest cover * *micro*T_{max} *distance*) because forest cover and microclimate are inevitably correlated. The *micro*T_{max} *distance* is calculated using microclimate data that includes vegetation cover. Therefore, when including forest cover and *micro*T_{max} *distance* together as predictors in a GLMM, they are not independent, making it harder to reliably test for an interaction. We therefore used macroclimate in the first stage of the analysis to test for broad-scale patterns in how climate mediates responses to forest cover. In the second stage, we then examined microclimatic conditions to provide a potential mechanistic explanation for why responses to forest cover vary across macroclimatic gradients.

While macroclimates are indeed unlikely to represent actual climatic conditions that organisms experience inside forests, we include them in the first stage of the analysis because they are nonetheless of interest and relevance to scientists [16], especially when investigating large-scale patterns. As we have shown, macroclimatic distance to warm limit has a strong effect on sensitivity to forest cover, which means that future global macroclimatic changes are likely to alter bird responses to forest cover (as discussed on lines 339-342). Our use of macroclimate in the first stage of the analysis provides a necessary foundation for understanding and projecting broad-scale impacts of climate change on response to forest cover, while the microclimate analyses allow us to assess potential underlying mechanisms.

3-2. Another analytical question is why the study did not choose to compile more temporally-relevant forest cover data for each primary study (presumably doable), but instead used a blanket time point of year 2000. It seems to me that not doing the latter has the added drawback of bringing in a potential confounding effect on species response to forest cover: if the surveys of different study sites were conducted in different years, their different lengths of time since 2000 could conceivably confound the incidences being compared interspecifically or intraspecifically?

Thank you for this question. Yearly global forest cover data are not available at sufficiently high spatial resolution (30 m). We chose the Hansen dataset from 2000 because it provides a global map of percentage tree cover at 30 m spatial resolution, which allowed us to calculate landscape-scale forest cover using a consistent method for every site in our global dataset, thereby avoiding potential further confounding effects of compiling forest cover estimates across landcover datasets with different definitions of forest. The 30m spatial resolution is essential for quantifying the proportion of landscape-scale forest cover within a 600 m radius buffer around each site – a spatial scale that has been shown to be optimal for assessing bird responses to forest cover [17–21]. While yearly 30 m resolution forest cover maps are available for certain regions (e.g. MapBiomas for Brazil [22]), we are not aware of global annual forest cover maps at this spatial resolution. While there are other yearly landcover datasets available, their spatial resolution is too coarse for this study. For example, the MODIS landcover dataset is 500 m resolution [23] and the ESA CCI landcover dataset is 300 m resolution [24]. Our approach of using forest cover data from the year 2000 to analyse species responses in the years following 2000 is consistent with other global studies on biodiversity responses to forest change, e.g. [14,25,26].

We agree that this could have the drawback of bringing in the potential confounding effect on species incidence of time since 2000. However, we included random intercepts for each study in our statistical model, which would help mitigate this potential confounding effect. We have also now included a figure, referenced on line 391, which shows the timespan of all studies to make clearer the distribution of lengths of time since 2000 (Figure S19, Appendix 11).

3-3. An additional analytical question is about the choice of functional traits: I was struck by the fact that the list of traits considered does not include what seems to me to be a most relevant trait – i.e., the level to which a given species is dependent on (native) forest habitat. I

understand that data for this trait are not readily available from existing databases, and that is probably a barrier to the study including it, but conceptually, I think it would be a key trait to consider in the analysis here. Given the large number of species included in this study and the rather loose criteria by which these species were selected, I would imagine there to be large variations in this trait among the study species; I also think it is conceivable that this trait can influence how species responds to forest cover and how climate influences this response (note that the study concluded that traits were overall irrelevant to these effects).

Thank you for this thoughtful suggestion. We have now incorporated a measure of species' forest dependence into the analysis. We used species classifications of primary habitat preference from AVONET [27] to categorise species into those for which forest is the primary habitat and those for which forest is not the primary habitat. All species in the latter category still use forest habitats, as inclusion in our study required that forest be listed as one of the species' habitat affiliations (based on IUCN classifications).We found that species with forest as their primary habitat preference were more likely to show the overall trend of heightened sensitivity to forest cover close to their warm limit. This result is described on lines 177-182, and shown in Extended Data Figure 4A and Table S8.

Lines 177-182 are as follows: *"Primary habitat also mediated variation in how T_{max} distance affects responses to forest cover. Species with forest as their primary habitat preference were more likely to show positive responses to forest cover close to T_{max}, and more negative responses to forest cover further from T_{max} (Extended Data Figure 4; Table S8)."*

3-4. Yet another analytical question is about the assessment of intraspecific variations in species response to forest cover and how this response is influenced by climate, presumably the central question of the study. From the manuscript, it seems to me that the analyses in which this question was tested were the same models used to test for interspecific variations. It is not clear to me why this is (why not more directly test for intraspecific variations), or how the interspecific models could inform on the intraspecific variations (the study mentioned derivatives/partial effects from these models, but I found the explanation in this part – in the Results section as well as the later Methods section – unclear with regard to exactly what was done and how it could provide the inference needed).

Thank you for raising this. There is both an inter- and intra- specific component to the pattern we identified, but we can see that this was not analysed clearly enough in the previous

version. In the revised manuscript, we have restructured the text in the Methods and Results to more clearly explain how the effect of *T_{max} distance* on responses to forest cover within populations of the same species was disentangled from this variation among populations of different species. In particular, we have added an analysis that directly tests for the intraspecific variation by quantifying the percentage of species for which populations within the species were more sensitive to forest cover when located close to their species' warm limit. (Extended Data Figure 3).

The revised text in the Results is as follows: *"The proximity of a population to its species' warm limit (T_{max} distance) is an important modulator of the variation in how forest cover affects incidence not just among species (Figure 2), but also among populations of the same species (Extended Data Figure 3). To study this relationship within each species, we calculated the partial effect of the model with respect to forest cover* [28]*, and applied this calculation to the species-level random effects to provide a metric of response to forest cover at any given T_{max} distance for each species. A positive value of this metric indicated an increase in population incidence with increasing forest cover and a negative value indicated a decrease in incidence with increasing forest cover. Out of the 2,262 species in our study, 2,143 could be used to assess how T_{max} distance modulates within-species responses to forest cover, as these species exhibited some variation in T_{max} distance either within or across studies. Of these 2,143 species, 1,114 (52%) showed increasingly positive effects of forest cover on incidence when located closer to T_{max} (Extended Data Figure 3). We also calculated the partial effect of forest cover for the fixed-effects component of the model. This showed an overall mean trend in which increasing forest cover had a positive effect on incidence at temperatures close to T_{max}, and a shift in the average response to a negative effect of increasing forest cover on incidence at temperatures > 15.8 °C cooler than T_{max}, when all species are analysed together (Figure 2C)"* (lines 126-146).

The revised text in the Methods is as follows: *"To analyse variation in responses to forest cover both across all species, and within each species, we took the model derivative with respect to forest cover for the fixed effects, and species-specific random effects, respectively* [28] *(Figure 2C). A positive value of this derivative indicates an increase in incidence with increasing forest cover and a negative value indicates a decrease in incidence with increasing forest cover. Taking the derivative with respect to forest cover for the fixed effect model estimates provides a metric of sensitivity to forest cover at each T_{max} distance, averaged*

across all species. Applying this calculation to the species-specific random provides separate estimates for every species of the sensitivity to forest cover at each T_{max} *distance* [28].

We visualised both the overall fixed effect derivatives and the species-specific random effect derivatives to characterise how the overall average response to forest cover varies with T_{max} *distance, and to illustrate heterogeneity in these patterns among species (Figure 2C, Extended Data Figures 3 & 4). To summarise the direction and relative magnitude of the association between* T_{max} *distance and the response to forest cover within each species, we fitted a separate linear regression for each species, with* T_{max} *distance as the predictor and the derivative with respect to forest cover as the response. These regressions were used solely as descriptive summaries of the direction and relative magnitude of species-specific trends. Formal statistical inference was not assessed because* T_{max} *distance served both as the predictor in these regressions and as an input to the derivative calculations, resulting in no residual variance around the fitted relationships." (lines 628-647).*

3-5. Finally and probably a nuisance question: why did the study not directly use phylogenetic regressions to account for species' phylogenetic non-independence? In its current form, the study simply "tested for phylogenetic correlation in the species-level random effects" (line 483).

Thanks for picking up on this. We did not use phylogenetic regressions as our primary analytical approach because our analyses indicated that phylogenetic non-independence among species was minimal (Table S17). When we tested for phylogenetic signal in species-level intercepts and coefficients using PGLS and found consistently low values of λ (0.029–0.084). Given this weak phylogenetic signal, we chose to retain a non-phylogenetic mixed-effects framework in our main analyses, which allowed greater flexibility in modelling hierarchical structure (study and site) and species-specific random slopes.

Nonetheless, to further address the reviewer's question and to ensure our conclusions were robust, we conducted an additional sensitivity test in which phylogeny was explicitly incorporated as a structured random effect. Accounting for phylogenetic relatedness did not alter the direction, magnitude, or significance of the fixed effects (Appendix 3), confirming that phylogenetic non-independence does not influence our results.

We have now clarified our reasoning and discussed this additional sensitivity test on lines 610-615 in the Methods: *"To test for phylogenetic correlation in the species-level random*

effects, we used PGLS to estimate λ, a measure of phylogenetic signal. Phylogenetic signal was consistently low (λ = 0.029–0.084). Consequently, phylogeny was not included in the main analytical models (Appendix 3). As a sensitivity test, we fitted a phylogenetic GLMM in which phylogeny was incorporated as a structured random effect. The inclusion of phylogeny did not affect our results (Appendix 3)."

4. While the writing is generally clear, I think some important methodological information is missing from the main text (even though it is provided later in Methods). Notable examples include:

the criteria used to select study species (line 54-58)

Thank you for pointing this out. To ensure that the criteria used to select species is described in the main text as well as the Methods, we have added to the main text that "*We restricted our analysis to species for which forest was listed as one of their habitat affiliations according to the IUCN habitat classification* [12]*" (lines 60-61).*

the rationale for selecting a 600-m buffer in quantifying forest cover (line 62; note that the explanation provided in the Methods section on this point – in line 338-341 – is also not very clear),

Thank you for picking up on this. We have now summarised the rationale for the 600-m buffer in the main text: "*This buffer size reflects the sampling design of many of the original studies in the dataset, and was shown to be optimal when assessing bird responses to forest cover* [17–20,29] *"* (lines 71-73).

We have also rewritten the explanation in the Methods to enhance its clarity: "*This buffer distance matches the sampling design used in many of the original studies included in the dataset and therefore minimises spatial overlap among buffers. A 600 m buffer has also been shown to be optimal for assessing bird responses to forest cover* [17–21]*. Sensitivity analyses showed that using alternative buffer sizes of 400 m or 800 m – scales which are also sometimes used in studies of forest cover effects on bird communities* [30] *– did not affect our results (Appendix 7)"* (lines 427-435).

and the model structure used to assess the effects of predictor variables (the first paragraph of the Results and discussion section).

We have now added more detail about the model structure to the first paragraph of the Results and Discussion. Lines 107-109 now read: "*We investigated how T_{max} distance and forest cover affect probability of species' incidence using a binomial GLMM. This model included forest cover proportion, T_{max} distance and their interaction as fixed-effect predictors of incidence.*"

It also seems to me that for a number of important methodological decisions, the manuscript did not explain how they were made or done. Examples include the time stamp of the databases consulted when compiling the dataset used in the study,

We have added on line 366 that we accessed the databases in January 2024.

how the random intercepts were structured that accounted for "variations in sampling methods, detection probabilities and sampling efforts among studies" (line 321-323),

We have revised this sentence: *"We used random intercepts in statistical models to account for variation among studies"* (line 411). We also show the structure of the random effects on lines 605 and 608 in the Methods.

and the rationale for choosing the list of traits considered – either in the main text or Methods sections

We chose traits for which we had *a priori* hypotheses regarding how they would affect sensitivity to forest cover or temperature, based on evidence from previous studies in the literature.

As explained on lines 169-171 of the main text: *"We chose these traits because they have been shown to affect species' sensitivity to forest cover or temperature* [14,31,32]*."*

We have now also added a more detail about each hypothesis and the reasons for selecting each trait in the Methods: *"We investigated whether species' traits could explain how much their T_{max} distance affects their response to forest cover. We chose the following traits because they have been shown to affect species sensitivity to forest cover or temperature: geographic range size, primary habitat preference, nest type, migration, body mass, hand-wing index, primary foraging height and primary diet. Specifically, species with smaller ranges have been shown to be more likely to have higher sensitivity to forest cover* [33]*. Species with forest as their primary habitat preference would be expected to be more sensitive to forest cover* [34]*.*

Nest type can affect responses to temperature as open nests tend to be less thermally buffered than closed nests [32]*. Body mass could also affect responses to temperature as climatic warming tends to favour smaller-bodied species* [35]*. Species with lower dispersal abilities (measured by the hand-wing index) have been shown to be more sensitive to changes in forest cover as they are less able to cross gaps between habitat patches* [14]*. Foraging height and diet can also affect sensitivity, with understorey species and insectivorous species often showing higher sensitivity to forest fragmentation* [31]*. We also included an analysis of migratory vs. non-migratory species to test whether the inclusion of migratory species in our main analyses affects the temperature mediation of responses to forest cover"* (lines 491-506).

References

1. Williams, J. J. & Newbold, T. Vertebrate responses to human land use are influenced by their proximity to climatic tolerance limits. *Divers. Distrib.* **27**, 1308–1323 (2021).
2. Cohen, J. M. & Jetz, W. Geographic redistributions are insufficient to mitigate exposure to climate change in North American birds. *Nat. Ecol. Evol.* **9**, 1234–1244 (2025).
3. Cohen, J. M., Fink, D. & Zuckerberg, B. Spatial and seasonal variation in thermal sensitivity within North American bird species. *Proc. R. Soc. B Biol. Sci.* **290**, 20231398 (2023).
4. Copernicus Climate Change Service. Global bioclimatic indicators from 1979 to 2018 derived from reanalysis. ECMWF https://doi.org/10.24381/CDS.BCE175F0 (2021).
5. Deutsch, C. A. *et al.* Impacts of climate warming on terrestrial ectotherms across latitude. *Proc. Natl. Acad. Sci.* **105**, 6668–6672 (2008).
6. Hudson, L. N. *et al.* The database of the PREDICTS (Projecting Responses of Ecological Diversity In Changing Terrestrial Systems) project. *Ecol. Evol.* **7**, 145–188 (2017).
7. Pfeifer, M. *et al.* BIOFRAG – a new database for analyzing BIOdiversity responses to forest FRAGmentation. *Ecol. Evol.* **4**, 1524–1537 (2014).
8. Hasui, É. *et al.* ATLANTIC BIRDS: a data set of bird species from the Brazilian Atlantic Forest. *Ecology* **99**, 497–497 (2018).
9. Johnson, D. H. In Defense of Indices: The Case of Bird Surveys. *J. Wildl. Manag.* **72**, 857–868 (2008).
10. Banks-Leite, C. *et al.* Assessing the utility of statistical adjustments for imperfect detection in tropical conservation science. *J. Appl. Ecol.* **51**, 849–859 (2014).

11. Olson, D. M. *et al.* Terrestrial Ecoregions of the World: A New Map of Life on Earth: A new global map of terrestrial ecoregions provides an innovative tool for conserving biodiversity. *BioScience* **51**, 933–938 (2001).
12. IUCN. The IUCN Red List of Threatened Species. *IUCN Red List of Threatened Species* https://www.iucnredlist.org/en (2024).
13. Betts, M. G. *et al.* Extinction filters mediate the global effects of habitat fragmentation on animals. *Science* **366**, 1236–1239 (2019).
14. Weeks, T. L. *et al.* Climate-driven variation in dispersal ability predicts responses to forest fragmentation in birds. *Nat. Ecol. Evol.* **7**, 1079–1091 (2023).
15. Hua, F. *et al.* Ecological filtering shapes the impacts of agricultural deforestation on biodiversity. *Nat. Ecol. Evol.* **8**, 251–266 (2024).
16. Kemppinen, J. *et al.* Microclimate, an important part of ecology and biogeography. *Glob. Ecol. Biogeogr.* **33**, e13834 (2024).
17. Banks-Leite, C. *et al.* Using ecological thresholds to evaluate the costs and benefits of set-asides in a biodiversity hotspot. *Science* **345**, 1041–1045 (2014).
18. Boscolo, D. & Metzger, J. P. Is bird incidence in Atlantic forest fragments influenced by landscape patterns at multiple scales? *Landsc. Ecol.* **24**, 907–918 (2009).
19. Morante-Filho, J. C., Arroyo-Rodríguez, V. & Faria, D. Patterns and predictors of β-diversity in the fragmented Brazilian Atlantic forest: a multiscale analysis of forest specialist and generalist birds. *J. Anim. Ecol.* **85**, 240–250 (2016).
20. Hatfield, J. H., Orme, C. D. L. & Banks-Leite, C. Using functional connectivity to predict potential meta-population sizes in the Brazilian Atlantic Forest. *Perspect. Ecol. Conserv.* **16**, 215–220 (2018).
21. Martínez-Penados, A. L., Arroyo-Rodríguez, V., Morante-Filho, J. C., Pinel-Ramos, E. J. & Schondube, J. Old-growth forests are critical to safeguard tropical birds in complex landscape mosaics exposed to slash-and-burn agriculture. *Landsc. Ecol.* **39**, 118 (2024).
22. Souza, C. M. *et al.* Reconstructing Three Decades of Land Use and Land Cover Changes in Brazilian Biomes with Landsat Archive and Earth Engine. *Remote Sens.* **12**, 2735 (2020).
23. Friedl, M. & Sulla-Menashe, D. MODIS/Terra+Aqua Land Cover Type Yearly L3 Global 500m SIN Grid V061. NASA EOSDIS Land Processes Distributed Active Archive Center https://doi.org/10.5067/MODIS/MCD12Q1.061 (2022).
24. ESA Land Cover CCI project team & Defourny, P. ESA Land Cover Climate Change Initiative: Global Land Cover Maps.

25. Betts, M. G. *et al.* Extinction filters mediate the global effects of habitat fragmentation on animals. *Science* **366**, 1236–1239 (2019).
26. Pfeifer, M. *et al.* Creation of forest edges has a global impact on forest vertebrates. *Nature* **551**, 187–191 (2017).
27. Tobias, J. A. *et al.* AVONET: morphological, ecological and geographical data for all birds. *Ecol. Lett.* **25**, 581–597 (2022).
28. Bellotto-Trigo, F. C. *et al.* Intraspecific variation in sensitivity to habitat fragmentation is influenced by forest cover and distance to the range edge. *Biol. Conserv.* **284**, 110167 (2023).
29. Martínez-Penados, A. L., Arroyo-Rodríguez, V., Morante-Filho, J. C., Pinel-Ramos, E. J. & Schondube, J. Old-growth forests are critical to safeguard tropical birds in complex landscape mosaics exposed to slash-and-burn agriculture. *Landsc. Ecol.* **39**, 118 (2024).
30. Arroyo-Rodríguez, V. *et al.* Does a Species' Mobility Determine the Scale at Which It Is Influenced by the Surrounding Landscape Pattern? *Curr. Landsc. Ecol. Rep.* **8**, 23–33 (2023).
31. Lees, A. C. & Peres, C. A. Avian life-history determinants of local extinction risk in a hyper-fragmented neotropical forest landscape. *Anim. Conserv.* **11**, 128–137 (2008).
32. Lauck, K. S. *et al.* Agriculture and hot temperatures interactively erode the nest success of habitat generalist birds across the United States. *Science* **382**, 290–294 (2023).
33. Ausprey, I. J., Newell, F. L. & Robinson, S. K. Sensitivity of tropical montane birds to anthropogenic disturbance and management strategies for their conservation in agricultural landscapes. *Conserv. Biol.* **37**, e14136 (2023).
34. Keinath, D. A. *et al.* A global analysis of traits predicting species sensitivity to habitat fragmentation. *Glob. Ecol. Biogeogr.* **26**, 115–127 (2017).
35. Prokosch, J., Bernitz, Z., Bernitz, H., Erni, B. & Altwegg, R. Are animals shrinking due to climate change? Temperature-mediated selection on body mass in mountain wagtails. *Oecologia* **189**, 841–849 (2019).

Reviewer 1

I thank the authors for their careful and thorough responses to my previous comments. I have reviewed the revised manuscript and response letter, and I am satisfied that my concerns have been addressed.

Thank you for taking the time to review our manuscript and for the valuable feedback you have shared.

In particular, the authors have clarified the interpretation of Tmax as a realised macroclimatic warm limit rather than a physiological threshold, added appropriate caveats regarding habitat-specific exposure and thermally buffered microhabitats, incorporated range size and primary habitat affiliation into the trait analyses, provided additional detail on the microclimate validation datasets, and added useful analyses addressing concerns related to interannual variation in BIO05, biome-level patterns, latitude, and among-species variation in responses.

Thank you again for these helpful suggestions which improved the manuscript.

I have a minor comment regarding the new analyses whereby 52% of species showed the predicted within-species pattern (populations closer to the species' warm limits exhibit greater sensitivity to forest cover). Simply, this is only just over half, and I encourage the authors to ensure that broad claims about intraspecific responses are framed as average tendencies rather than general rules. More specifically, the wording in the abstract, fig 2 caption (eg. The location of a population relative to its species' realised warm tolerance limit (Tmax distance) mediates both inter- and intraspecific variation in how forest cover affects bird incidence), as well as the section heading for the corresponding results (eg. Thermal exposure explains inter- and intraspecific variation) could be toned down a bit more to emphasise that Tmax distance explains an important component of variation, while substantial species-level heterogeneity remains.

Thank you for raising this. We agree that there is substantial species-level heterogeneity. We have toned down the wording in the following areas:

1) Abstract: we have removed the word "*strongly*" in *"… strongly mediated by temperature*" (line 8) and have added the words "*tend to*" on lines 9 and 12, e.g. "*populations in warmer macroclimates tend to decline …* "

2) Fig 2 caption: this now reads "*The location of a population relative to its species' realised warm tolerance limit (T_{max} distance) can mediate variation in how forest cover affects bird incidence.*"
3) Section heading: this now reads *"Thermal exposure can explain variation in sensitivity to forest cover"*
4) Line 137 – when reporting the percentage of species that showed the predicted within-species pattern, we have added *"... indicating substantial species-level heterogeneity*"

Other than the above, I have no further substantive concerns and would support publication.

Reviewer 2

Thank you to the authors for their thoughtful revision of the manuscript. I think overall, this revision has successfully addressed the previous concerns, and is shaping up to be a strong contribution to the journal. I have the following, mostly small, comments for the authors to consider.

Thank you for taking the time to review our manuscript and for the valuable feedback you have shared.

1. Regarding the current framing of the study as a direct test of species response to forest cover that can inform the bigger question of species response to deforestation: I think this is a sensible way to frame the study, and the authors did a good job making the case for the linkage between what the study did exactly and its 'bigger-question' interpretations in their response letter. But I think the entry of the framing in the manuscript should be improved a bit more. Currently, the specific topic of 'bird response to forest cover' enters abruptly right after the manuscript opens very broadly with land use change, without any transition to link it with the preceding content meant essentially to set the scene for deforestation. The argument needed for this linkage enters only later in the next paragraph (lines 66-68) – after **the** paragraph on what the study did and why it is relevant to the broader question. I think when the manuscript brings up the study aim, which is about specie response to forest cover, it should explain and clarify right there the rationale of this aim – why it is a fitting way to respond to the broader issue and its gaps. In its current form, when the study enters the picture, there remains a disconnect between what the study did and what it is apparently set up to do.

Thank you for the feedback and for your careful consideration of the clarity of the study's framing. Since there are so few large-scale studies directly testing how temporal changes in land-use affect temporal changes in biodiversity, it is generally accepted to use the term "land-use change" broadly to refer to the environmental predictor variables in a study that relies on a space-for-time substitution. Nonetheless, we have changed "land-use change" to "land-use" when discussing previous studies on lines 35 and 40. This implicitly sets up the rationale for the aim of our study (lines 50-51 – "Here, we investigate how and why temperature drives inter- and intraspecific variability in how forest birds respond to forest cover …"). We opted to keep the rationale and clarification for the space-for-time proxy on lines 66-68 in the paragraph that explains how the forest cover data was used as we think this is a cleaner structure for the Introduction.

2. Regarding the key finding of negative relationships between species incidence and forest cover in macroclimates further from species' warm limits (lines 116-117), one question the reader would naturally have is how so. Do you have any ecological interpretations for this counterintuitive finding, or could it rather be a statistical artefact? I think this counterintuitive finding should probably be given some discussion.

Thank you for raising this. We agree this is an interesting finding that readers may wonder about. Though it may seem counterintuitive, a combination of positive and negative biodiversity changes following forest loss have been documented globally in a variety of taxa (Daskalova et al. 2020 *Science*).

The latter half of our paper is entirely dedicated to explaining the ecological interpretation of the key finding of negative relationships between species incidence and forest cover in macroclimates further from species' warm limits (and positive relationships closer to species' warm limits). Figure 3B and lines 222-264 give our proposed ecological interpretation of this finding. To summarise, populations located in regions where they experience cooler-than-optimal temperatures, far from their warm limits, are pushed closer to their optimal temperatures at low forest cover. These optimal temperatures could have a variety of physiological or indirect environmental causes, such as via food availability. We encourage future studies to investigate the mechanistic processes underlying this pattern.

3. For the analysis on traits, the study essentially used multiple single regressions on

individual traits. But how reasonable is this, given that if multiple traits are relevant to species response, then assessing one trait at a time will inevitably generate biased estimates of the effect of individual traits? (This question pertains to the general rationale for running multiple regressions rather than multiple single regressions.)

This is a good question, thank you for raising it. The problem with combining all traits into one model is that many of these traits are highly correlated. We opted to fit a single mixed effects model for each trait so that we could directly quantify the role of each trait.

4. Regarding the presentation of methods information, I think this version is much improved. But there remain some small issues:

a. The sentence in lines 129-133 needs improved clarity in terms of its meaning.

Thank you. We have revised this sentence. It now reads: "*To study this relationship within each species, we calculated the derivative of the species-level random effects*[20] *to provide a metric of response to forest cover at any given* T_{max} *distance for each species.*"

b. For the analysis on functional traits, the sentence in lines 187-188 noted the traits that are not (strongly) relevant as 'morphological or functional traits'. Given the study's findings, this term should not include range size and species' dependence on forest habitat (these two traits were indeed found to be relevant), which can be at odds with the typical interpretation of this term. I think rephrasing of this statement is probably warranted.

Thank you for picking up on this, we have changed *"functional traits"* to *"dietary traits"* (line 185).

c. Does the word 'derivative' when discussing model interpretation and results mean slope (coefficient of predictor variables; e.g. as used in line 629)? If so, slope / coefficient is a much more intuitive and familiar term than 'derivative'.

This refers to the derivative calculated on the slope coefficient.

5. Some small writing issues:

a. Grammar of the sentence in lines 175-176 needs checking.

Thanks. We have changed "*Whereas*" to "*By contrast*"

b. The word 'deforestation' in line 265 should be changed to 'forest cover'.

Thanks. We have revised this on lines 257 and 262.

c. The sentence in lines 634-635 should be checked for sentence completion and clarity.

Good catch! Thank you. We have revised this sentence. It now reads: *"Applying this calculation to the species-specific random effects provides separate estimates for every species of their sensitivity to forest cover at each* T_{max} *distance* [20]*."*
